# Muscle mitochondria, function, mass, and quality of life in prostate cancer during androgen deprivation therapy

**DOI:** 10.1038/s41467-026-73542-x

**Published:** 2026-05-27

**Authors:** L. Caeiro, L. J. Anderson, A. Dash, D. J. Marcinek, H. L. Kerr, L. Paulsen, G. Miranda, S. Jaramillo Quiroz, T. Vaisar, M. A. Krueger, N. L. Acosta-Vega, S. A. Gharib, J. M. Garcia

**Affiliations:** 1 Geriatric Research, Education and Clinical Center, Veterans Affairs Puget Sound Health Care System, Seattle, WA USA; 2 Gerontology and Geriatric Medicine, Department of Medicine, University of Washington, Seattle, WA USA; 3 Department of Urology, University of Washington, Seattle, WA USA; 4 Department of Radiology and Laboratory Medicine and Pathology, University of Washington, Seattle, WA USA; 5 Metabolism, Endocrinology, and Nutrition, Department of Medicine, University of Washington, Seattle, WA USA; 6 Computational Medicine Core, Center for Lung Biology, Division of Pulmonary, Critical Care and Sleep Medicine, Department of Medicine, University of Washington, Seattle, WA USA

**Keywords:** Prostate cancer, Fatigue

## Abstract

Prostate cancer (PCa) negatively impacts muscle mass, physical function, and patient-reported outcomes (PROs), while androgen deprivation therapy (ADT) exacerbates these effects. Mitochondria are important for muscle function but their role in PCa patients undergoing ADT is not well-established. Our study characterizes the relationship between muscle mass, strength, endurance, PROs, and mitochondria in PCa patients over six months of ADT. Prior to ADT, higher mitochondrial function and endurance are associated with better PROs; whereas higher appendicular lean mass (ALM) correlate with worse PROs. Greater baseline VO_2_ peak and mitochondrial function predict smaller declines in ALM, muscle function, and PROs. Proteomics analysis indicates mitochondrial dysfunction and upregulation of extracellular matrix organization, inflammation and coagulation-related pathways with ADT. Our findings suggest that mitochondrial function plays a role in muscle endurance and PROs in PCa. These data may help select patients and outcomes for clinical trials. Future studies should test whether targeting mitochondria can improve physical function and PROs in PCa.

## Abstract (web summary)

Prostate cancer and androgen deprivation therapy (ADT) impair muscle function and quality of life, yet mitochondrial involvement remains unclear. The authors show that greater mitochondrial function correlates with better endurance, quality of life, and smaller declines in these parameters post-ADT

## Introduction

Prostate cancer (PCa) is the most prevalent cancer among men after skin cancer and the second leading cause of cancer deaths^1^. PCa often results in decreased muscle mass, reduced physical function, increased fatigue, and decreased quality of life (QOL)^2–4^, all of which are linked to poor overall survival^5–8^. Androgen deprivation therapy (ADT) is the primary treatment for advanced PCa, but it paradoxically exacerbates these complications, further reducing muscle mass and function and worsening QOL^9,10^. Emerging aggressive therapies for PCa intensify targeting the androgen-driven mechanism^11,12^ and are likely to worsen these side effects. Given the significance of these complications, they represent a critical unmet need in PCa care.

In older adults, declines in muscle mass correlate with lower muscle strength but not necessarily with reductions in physical performance^13^. In clinical trials targeting cancer-related decreases in muscle mass and function, interventions that increased muscle mass did not improve physical function^14^. This disconnect between muscle mass and function is a critical barrier to therapeutic development for these complications, and in PCa patients undergoing ADT, it remains unexplored. A more thorough understanding of the relationship between muscle mass, functional performance, and patient-reported outcomes (PROs) before and during ADT could help establish effective tools for measuring changes in these outcomes, aiding in the development of novel therapies.

The effect of ADT on physical function, muscle mass, and QOL is variable, and the factors that predict the severity to which these outcomes are impacted have not been fully elucidated. Higher Gleason score, more comorbidities, and sedentarism, but not hand grip strength (HGS) or chair rise time prior to ADT initiation, are reportedly associated with greater declines in self-reported physical function after twelve months of ADT^10^. Identification of predictors for worse ADT-induced impact on objective physical function, muscle mass, or other PROs could guide clinicians to anticipate which patients are at higher risk for these side effects. This may also allow earlier interventions and/or tailored treatment strategies to mitigate PCa and ADT side effects.

The underlying molecular pathways contributing to the loss of muscle mass and function and poor QOL caused by PCa and worsened by ADT are also incompletely characterized. Mitochondria are thought to play a central role in age-induced sarcopenia^15^, but their role in PCa and the effects that ADT has on mitochondrial function are not well understood. The goals of this study are to characterize the relationship between muscle mass, strength, endurance, PROs, and mitochondrial function in men with PCa before and during a 6-month course of ADT, and to establish predictors for ADT-induced side effects.

Here, we show that mitochondrial function and endurance are associated with better PROs, whereas appendicular lean mass correlates with worse PROs in patients with PCa before ADT. We further show that higher pre-ADT VO_2_ peak and mitochondria function predict smaller decreases in muscle mass, physical performance, and PROs during six months of ADT. Proteomic analysis revealed mitochondrial dysfunction and enrichment of extracellular matrix, inflammatory, and coagulation pathways after ADT. These results suggest that mitochondrial function relates to better endurance and patient-reported outcomes and may help identify individuals most vulnerable to ADT-related side effects.

## Results

### Study participants

Patients with PCa about to undergo ADT were recruited and evaluated for six months. Of the 60 patients enrolled, 59 completed the baseline visit, 57 the three-month visit, and 55 the six-month visit (Supplementary Fig. 1, consort diagram). Nineteen (32.2%) patients displayed lymph node or distant metastasis, 48 (81.3%) presented tumor grade group 3 or higher, 36 (61%) received concomitant radiotherapy, and 55 (93.2%) were prescribed ADT in the form of a GnRH agonist (Table 1). There was no significant tumor progression during the six months that the patients were evaluated.

**Table 1:** Baseline characteristics

Mean (SD) or *N* (%)	*N* = *59*
Age (yrs.)	69.3 (6.9)
Body weight (kg)	87.2 (16.0)
Body mass index (kg/m^2^)	27.8 (4.1)
Tumor stage	
2	16 (27.1)
3	24 (40.7)
4	19 (32.2)
Total Gleason	
7	30 (50.8)
8	9 (15.3)
9	19 (32.2)
Undetermined	1 (1.7)
Grade group	
2	10 (16.9)
3/4	29 (49.1)
5	19 (32.2)
Undetermined	1 (1.7)
Radiation (yes)	36 (61)
ADT type	
GnRH agonist	55 (93.2)
GnRH antagonist/Orchiectomy	3 (5.1)
Unspecified	1 (1.7)
Race	
White	44 (74.6)
Black/African American	10 (16.9)
Asian/Nat. Hawaiian/Pac. Islander/Nat. American	4 (6.8)
Unknown	1 (1.7)
Body composition (DEXA)	*N* = *59*
Total mass (kg)	87.5 (16.0)
Total fat (kg)	25.9 (8.5)
Fat percent (%)	29.0 (5.4)
Lean mass (kg)	58.9 (9.0)
ALM (kg)	25.2 (4.0)
Physical function	*N* = *56–59*
VO_2_ peak (mL/kg/min) (*N* = 56)	19.2 (7.5)
Hand grip strength (kg) (*N* = 59)	40.3 (8.0)
6MWT (m) (*N* = 59)	510.3 (103.2)
Stair climb power (W) (*N* = 56)	
Stair climb power (W)	365.4 (113.3)
Non-sedentary physical activity (min/day) (*N* = 56)	79.4 (71.9)
Magnetic resonance spectroscopy	*N* = *30*
Maximum ATP synthesis (nM/s)	0.65 (0.2)
Ex vivo respirometry	*N* = *40–41*
State 2 (pmol/min)	70.9 (44.5)
State 3 (pmol/min)	483.4 (301.3)
State 4 (pmol/min)	86.7 (68.8)
State 3u (pmol/min)	546.7 (359.9)
FACT-P	*N* = *59*
Physical well-being (0–28)	24.2 (4.8)
Social family well-being (0–28)	21.0 (5.6)
Emotional well-being (0–24)	18.9 (4.0)
Functional well-being (0–28)	20.0 (6.7)
Prostate cancer additional (0–48)	32.3 (8.0)
Total FACT-P (0–156)	116.3 (21.9)
EORTC QLQ-C30	*N* = *58–59*
Quality of life	72.9 (21.8)
Physical functioning	85.1 (18.0)
Role functioning	84.6 (24.6)
Emotional functioning	79.4 (21.2)
Cognitive functioning	84.8 (21.9)
Social functioning	82.8 (24.4)
Fatigue	23.9 (22.1)
Nausea	3.4 (9.2)
Pain	26.0 (24.6)
Dyspnea	20.9 (26.2)
Insomnia	33.3 (32.8)
Appetite loss	14.1 (20.7)
Constipation	9.6 (22.4)
Diarrhea	7.9 (16.8)
Financial difficulties	15.3 (28.6)

Values are reported in either mean and standard deviation or number and percentage, depending on whether the variable is continuous or categorical, respectively. For the EORTC QLQ C30, scales range from 0 to 100. A high score for functional or quality of life status represents a high level of functioning and QOL, while a high score for a symptom scale represents worse symptoms.

*ADT* androgen deprivation therapy, *GnRH* gonadotropin-releasing hormone, *Nat* native, *Pac.* Pacific, *DEXA* dual-energy X-ray absorption, *ALM* appendicular lean mass, *6MWT* six-minute walk test, *W* watts, *min* minutes, *N* newtons, *AU* arbitrary units, *FACT-P* Functional Assessment of Cancer Therapy-Prostate, *EORTC QLQ-C30* European Organization for Research and Treatment of Cancer Quality of Life Questionnaire Core 30.

### Muscle endurance and mitochondrial function are positively associated with PROs in prostate cancer patients before starting ADT

Supplementary Figs. 2 and 3 illustrate baseline univariate correlations between measures of body composition, physical performance, PROs, and mitochondrial function. Body composition was assessed by dual-energy X-ray absorptiometry (DEXA) to measure total lean mass, appendicular lean mass (ALM; sum of lean mass in arms and legs), and total fat mass. Body mass index (BMI), ALM, and fat mass were all positively correlated with each other. Muscle strength, assessed by stair climb power (SCP) and HGS, was positively correlated with muscle endurance, measured by the six-minute walk test (6MWT), VO_2_ peak, and non-sedentary physical activity. ALM was positively correlated with HGS and SCP but not with 6MWT, VO_2_ peak, or physical activity. Finally, fat mass and BMI were negatively associated with VO_2_ peak.

PROs were assessed via two validated questionnaires, Functional Assessment of Cancer Therapy-Prostate (FACT-P) and European Organization for the Research and Treatment of Cancer Quality of Life (EORTC QLQ-C30). Greater BMI and ALM were associated with worse FACT-P physical well-being (PWB), PCa additional concerns (PCa AC), and QLQ-C30 functioning and symptom scores. Endurance measures were correlated with better FACT-P outcomes and QLQ-C30 QOL and functioning scores, and with lower QLQ-C30 fatigue. HGS presented no associations with PROs. To better understand the relationship between ALM, fat mass, and PROs, we performed stepwise multivariable linear regression analyses with age, ALM, fat mass, and metastasis as predictors (Table 2). ALM was a negative predictor of FACT-P PWB (β = –0.13, *p* < 0.001) and PCa-specific concerns (β = –0.011, *p* = 0.005), as well as EORTC QLQ-C30 scores for SF (β = –0.02, *p* = 0.023), and associated with worse fatigue (β = 0.05, *p* = 0.014). Fat mass was not a significant predictor for any of the PROs.

**Table 2:** Predictors of patient-reported outcomes in prostate cancer patients before undergoing ADT

Dependent variable	*N*	*R*^2^	Significant predictors	Unstd. B (95%)	*P*-value
FACT-P physical WB	59	0.21	ALM	−0.13 (−0.20, −0.006)	<0.001
FACT-P Pca AC	59	0.13	ALM	−0.011 (−0.02, −0.004)	0.005
EORTC QLQ C30 SF	59	0.08	ALM	−0.02 (−0.04, −0.003)	0.023
EORTC QLQ C30 fatigue	59	0.10	ALM	0.05 (0.01, 0.09)	0.014

Stepwise multivariable linear regression with baseline assessments of patient-reported outcomes as the dependent variable and age, ALM, fat mass, and metastasis as independent variables (predictors). Patient-reported outcome variables were log-transformed due to their non-normal distribution. *N* = 59.

*Unstd. B* unstandardized beta, *FACT-P* Functional Assessment of Cancer Therapy in Prostate Cancer, *WB* well-being, *Pca AC* Prostate Cancer Additional Concerns, *EORTC QLQ-C30* European Organization for Research and Treatment of Cancer Quality of Life Questionnaire Core 30, *SF* social functioning, *ALM* appendicular lean mass.

To identify independent predictors of physical function accounting for differences in fat and lean mass and other important variables, we used stepwise multivariable linear regression models with age, ALM, fat mass, and metastasis as predictors (Table 3). VO_2_ peak was included only in models for muscle strength (SCP, HGS), while HGS was included only for measures of endurance (VO_2_ peak, 6MWT). SCP was independently predicted by age (β = –4.3, *p* = 0.029), ALM (β = 10.1, *p* = 0.003), and VO_2_ peak (β = 245.9, *p* = 0.005). VO_2_ peak was associated with fat mass (β = –0.01, *p* < 0.001) and HGS (β = 0.01, *p* < 0.001). For the 6MWT, fat mass (β = –3.0, *p* = 0.045) and HGS (β = 0.006, *p* < 0.001) were significant predictors. HGS was independently associated with ALM (β = 0.80, *p* < 0.001), VO_2_ peak (β = 20.5, *p* < 0.001), and metastasis (β = –5.09, *p* = 0.006).

**Table 3:** Predictors of physical function in prostate cancer patients before undergoing ADT

Dependent variable	*N*	*R*^2^	Significant predictors	Unstd. B (95%)	*P*-value
Stair climb power	53	0.39	Age	−4.3 (−8.2, −0.46)	0.029
			ALM	10.1 (3.6, 16.5)	0.003
			VO_2_ peak	245.9 (78.3, 413.5)	0.005
VO_2_ peak^A^	56	0.37	Fat mass	−0.01 (−0.02, −0.006)	<0.001
			HGS	0.01 (0.005, 0.015)	<0.001
6MWT^A^	59	0.20	HGS	0.006 (0.003, 0.009)	<0.001
			Fat mass	−3.0 (−5.9, −0.07)	0.045
HGS	56	0.42	ALM	0.80 (0.38, 1.2)	<0.001
			VO_2_ peak	20.5 (10.32, 30.69)	<0.001
			Metastasis	−5.09 (−8.7, −1.53)	0.006

Stepwise multivariable linear regression with baseline assessments of physical function as the dependent variable and with age, ALM, fat mass, and metastasis as independent variables (predictors). VO_2_ peak was included only in models for muscle strength (SCP, HGS), while HGS was included only for endurance (VO_2_ peak, 6MWT). *N* = 53–59.

*Unstd. B* unstandardized beta, *ALM* appendicular lean mass, *HGS* hand grip strength, *6MWT* 6-Minute Walk Test.

^A^Log transformed.

Skeletal muscle mitochondrial function was evaluated both ex vivo in isolated mitochondria obtained from muscle biopsies of the quadriceps and in vivo through magnetic resonance spectroscopy (MRS), which assessed mitochondrial oxidative phosphorylation capacity (ATP Max) of the tibialis anterior. Ex vivo state 3 u mitochondria respiration, which is the equivalent to maximal uncoupled respiration, was positively correlated with in vivo ATP Max (*r* = 0.42, *p* = 0.048, *n* = 23) and QLQ-C30 physical functioning. ATP Max was negatively associated with BMI, ALM, fat mass, HGS, and QLQ-C30 fatigue and positively associated with VO_2_ peak, 6MWT, physical activity, FACT-P PWB, and QLQ-C30 QOL and functioning scores.

To better understand these findings, individuals were stratified based on the median values of VO_2_ peak, ATP Max, and State 3 u mitochondrial respiration (Supplementary Tables 1–3). Patients with a VO_2_ peak greater than the median had lower BMI and fat mass, and presented better performance on the 6MWT, SCP, and HGS, along with higher ATP Max and more favorable PROs. Individuals with ATP Max greater than the median had lower BMI, fat mass, ALM, and HGS, and demonstrated better performance on the 6MWT and higher VO_2_ peak. There was a trend toward better PROs in individuals with higher ATP Max. Moreover, individuals with greater State 3u mitochondrial respiration had higher ATP Max and more favorable PROs, with a trend toward better 6MWT performance.

### Changes after six months of ADT

**Fig. 1:**
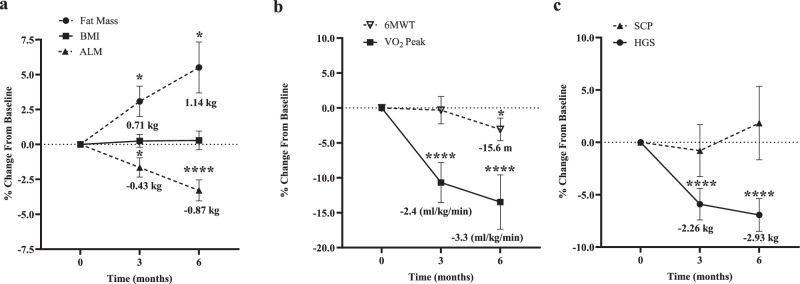
Changes induced by ADT. Changes in body composition (**a**) and physical function, including endurance (**b**) and strength (**c**) outcomes. Data are presented as mean values with SEM. Two-sided *p*-value corresponds to the beta-intercept from generalized estimated equations adjusted for tumor stage and age with no adjustments for multiple comparisons. The mean percentage changes are calculated relative to baseline: *p < 0.05; **p < 0.01; ****p < 0.001. Fat Mass: 3-month *p* = 0.003; 6-month *p* = 0.007. ALM: 3-month *p* = 0.014; 6-month *p* = <0.001. 6MWT: 6-month *p* = 0.011. VO_2_ Peak: 3-month *p* = <0.001; 6-month *p* = <0.001. HGS: 3-month *p* = <0.001; 6-month *p* = <0.001. Sample sizes at 3 and 6 months, respectively, were: BMI (58, 55), Fat mass (57, 55), ALM (57, 55), VO2 peak (54, 43), HGS (57, 55), 6MWT (53, 52), and SCP (52, 52). BMI body mass index, ALM appendicular lean mass, 6MWT six-minute walk test, SCP stair climb power, HGS hand grip strength. GraphPad Prism version 10.1.2 (GraphPad Software, Boston, MA) was used to create this figure.

Fat mass increased, and ALM decreased after three and six months of ADT, while BMI did not change (Fig. 1a). HGS and VO_2_ peak also decreased at both timepoints. 6MWT worsened after six months only, and SCP did not change (Fig. 1b, c). Non-sedentary physical activity showed a downward trend after six months (*p* = 0.051) (Supplementary Table 4).

The FACT-P subdomains for PWB, PCa AC, and total score declined at three and six months (Supplementary Table 5). FACT-P functional well-being decreased after three months but was only a trend at six months. For the QLQ-C30 (Supplementary Table 6), physical (PF) and social (SF) functioning decreased after six months, while fatigue increased after three and six months. Nausea, insomnia, and constipation increased after six months, while diarrhea increased at the three-month mark but was a trend at six months.

There was no change in mitochondrial function measured ex vivo or in vivo after six months of ADT (Supplementary Table 7).

### Baseline VO_2_ peak and mitochondrial function predict ADT-induced changes in ALM, function, and PROs

Univariate analysis of baseline variables and six-month changes in outcomes showed that baseline VO_2_ peak positively correlated with six-month relative change (%) in ALM and 6MWT and with six-month absolute change in QLQ-C30 PF and CF (Supplementary Figs. 4 and 5). Baseline mitochondrial state 3 respiration positively correlated with six-month change in ALM, SCP, FACT-P, PCa AC, and QLQ-C30 RF. ATP Max positively correlated with changes in FACT-P PCa AC. Baseline BMI negatively correlated with six-month change (%) in BMI, ALM, and 6MWT.

### Changes in ALM are positively linked to changes in muscle function and PROs

Six-month change in ALM positively correlated with change in 6MWT, SCP, total FACT-P, QLQ-C30 PF and CF, and negatively with appetite loss (Supplementary Figs. 6 and 7). Six-month change in VO_2_ peak negatively correlated with change in fat mass and positively with change in QLQ-C30 RF and SF. Appetite loss correlated with greater decreases in ALM, BMI, 6MWT, and SCP, and the six-month change in BMI positively correlated with the change in fat mass, ALM, 6MWT, and SCP. To further evaluate how changes in ALM, fat mass, and VO_2_ peak relate to changes in PROs and measures of function, we performed stepwise multivariable regression using 6-month percent change in ALM, fat mass, VO_2_ peak, age, and metastasis as predictors (Supplementary Table 8). Six-month change in ALM was a consistent independent predictor of less decreases in 6MWT, HGS, FACT-P, and several QLQ-C30 domains, including physical, cognitive, and social functioning, as well as reduced appetite loss. VO_2_ peak was independently associated with role functioning, and age also contributed to that model. No significant predictors for VO_2_ peak and SCP were found.

### Greater mitochondrial-related gene expression is associated with better muscle endurance

We also assessed skeletal muscle mRNA expression of genes involved in mitochondrial biogenesis and mitophagy after six months of ADT. Peroxisome proliferator-activated receptor gamma coactivator 1-alpha (PGC1-α) and mitochondrial transcription factor A (TFAM) are central regulators of mitochondrial biogenesis^16,17^, while nuclear respiratory factor 1 (NRF1) is a transcription factor downstream of PGC1-α^18^. Removal of damaged mitochondria by mitophagy is mediated by genes such as BCL2 protein-interacting protein 3 (BNIP3), PTEN-induced kinase 1 (PINK1), and PARK2, the gene that codes for Parkin^19–21^. No significant changes were observed in the expression after six months of ADT (Supplementary Fig. 8A). However, correlations showed that higher baseline PGC1-α expression was associated with greater VO_2_ peak and 6MWT (Supplementary Fig. 8B). Greater BNIP3, PINK1, and TFAM expression was associated with greater VO_2_ peak. Greater NRF1 expression at baseline correlated with higher state 3 and state 3u respiration. Moreover, greater baseline expressions of PGC1-α, BNIP3, PINK1, and TFAM were associated with a slight decrease in 6MWT after six months of ADT.

### Skeletal muscle proteomics before and after 6 months of ADT

**Fig. 2:**
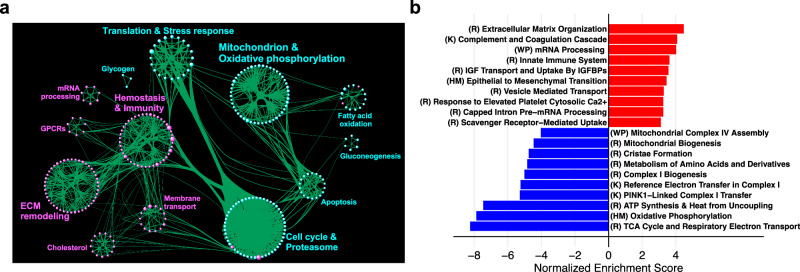
Gene Set Enrichment Analysis (GSEA) of skeletal muscle proteome after six months of ADT. **a** Network depiction of enriched pathways identified by GSEA (FDR < 0.05). Each node represents a gene set, and edges connect node pairs with >50% overlapping protein members. Note the emergence of labeled biological modules comprised of densely connected gene sets. Magenta represents upregulated, and cyan denotes downregulated gene sets. **b** Highly upregulated (red) and downregulated (blue) pathways after six months of ADT, ranked by normalized enrichment score (NES). R Reactome, K Kyoto Encyclopedia of Genes and Genomes, WP wiki pathways, HM Hallmark, IGF insulin-like growth factor, IGFBPs IGF binding proteins, ECM extracellular matrix, UCP1 uncoupling protein 1, OXPHOS oxidative phosphorylation, TCA tricarboxylic acid cycle, ALM appendicular lean mass, HGS hand grip strength, PWB physical well-being, GPCRs G-protein coupled receptors.

**Fig. 3:**
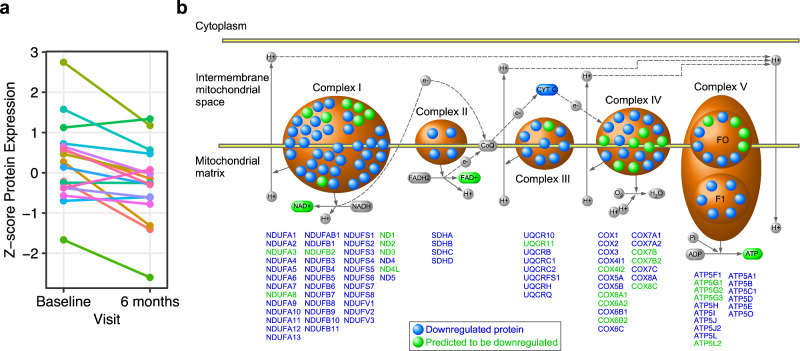
Mitochondrion-associated protein changes in skeletal muscle following six months of ADT. **a** Z-scores of abundance values of leading-edge proteins involved in mitochondrial processes as identified by GSEA at baseline and after 6 months of ADT. Changes were assessed using a two-sided paired *t*-test, which showed significant downregulation (*p* < 0.01). Each line represents a single individual. **b** Functional mapping of leading-edge proteins to mitochondria complexes using Ingenuity Pathway Analysis (IPA). Downregulated proteins are shown in blue, and proteins predicted to be downregulated by IPA are shown in green. This figure was created with Ingenuity Pathway Analysis (IPA) Analysis Match Explore from Qiagen^88^. NAD nicotinamide adenine dinucleotide, FAD flavin adenine dinucleotide, ATP adenosine triphosphate.

Proteomic analyses in skeletal muscle were performed in a subset of patients (*N* = 19) using data-independent acquisition liquid chromatography-tandem mass spectrometry (DIA LC-MS). After six months of ADT, Gene Set Enrichment Analysis (GSEA) revealed widespread perturbations in the proteomic landscape of skeletal muscle of PCa patients (Fig. 2a). We found that the most upregulated pathways were related to extracellular matrix (ECM) organization, inflammation, and coagulation processes while the most downregulated were related to mitochondrial function (Fig. 2b). To further elucidate the changes related to mitochondrial function, we focused on the leading-edge proteins from GSEA, which are primarily responsible for the enrichment of mitochondrial-related pathways. The average abundance of these proteins significantly decreased after six months of ADT (*p* = 0.0014) (Fig. 3a). To gain a more granular perspective, we leveraged the Ingenuity Pathway Analysis (IPA) Knowledge Base (Qiagen, Redwood City, CA) to overlay mitochondrial leading-edge proteomic profiles and predict differential regulation of specific mitochondrial components. Figure 3b illustrates how major complexes of the electron transport chain are downregulated after ADT. Nicotinamide adenine dinucleotide (NAD) was predicted to be downregulated, although our mass spectrometry measurements indicate that NAD levels did not change (Supplementary Fig. 9).

**Fig. 4:**
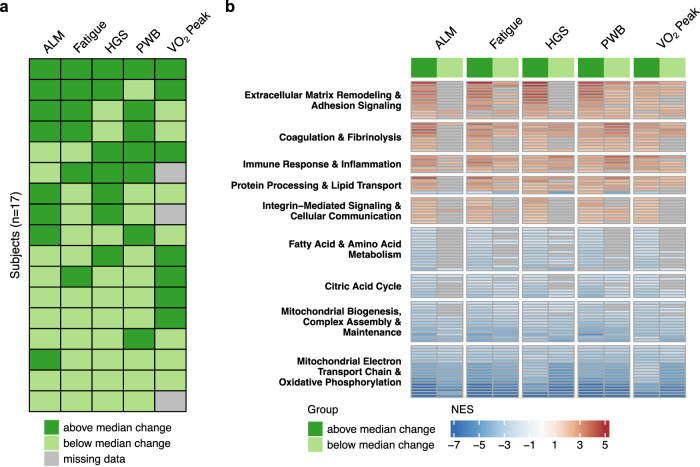
Pathway enrichment differences between the groups below and above the median change following ADT. Above the median change indicates more severe functional decline. **a** Categorization of individuals as above (dark green) or below (light green) the median change based on the group median six-month change after ADT in appendicular lean mass (ALM), fatigue, hand grip strength (HGS), physical well-being (PWB), or VO₂ peak. **b** Comparative heatmaps of differentially enriched pathways in below and above the median change groups (FDR < 0.05). Each cell in the heatmap represents an individual pathway, with the normalized enrichment scores (NES) highlighting the degree and direction of enrichment. Non-significant gene sets are shown in gray. ALM appendicular lean mass, HGS hand grip strength, PWB physical well-being.

To assess the differential impact of ADT, individuals were categorized based on their median six-month change in ALM, fatigue, HGS, PWB, or VO_2_ peak into above and below the median change, with above the median change indicating a more severe decline in functional performance. Although there was incomplete overlap between these domains across patients (Fig. 4a), the above median change group exhibited more pronounced downregulation of pathways involved in mitochondrial biogenesis, fatty acid and amino acid metabolism, and citric acid cycle compared to the below median change group across a range of measures. Interestingly, only the above median change group showed consistent upregulation of pathways related to immune response and inflammation, ECM remodeling, protein processing, and coagulation (Fig. 4b). Taken together, these findings indicate that individuals more severely impacted by ADT elicit a more profound perturbation in their skeletal muscle proteomic programs.

## Discussion

The negative impact of PCa and ADT on physical function and quality of life poses a substantial burden on patients. Barriers to developing effective treatments include our incomplete understanding of the underlying mechanisms, the absence of reliable predictors of the extent to which individual patients will be affected, and the lack of validated assessment tools. In men with PCa starting ADT, we observed that greater endurance and mitochondrial function, but not HGS or ALM, are associated with better PROs and lower symptom burden. Higher baseline VO_2_ peak and mitochondrial respiration were linked to preservation of ALM, muscle function, and PROs after six months of ADT. Patients with smaller ALM declines maintained 6MWT, SCP, and PROs, and those with smaller VO_2_ peak and 6MWT declines also displayed PRO preservation. While mitochondrial respiration remained unchanged after six months of ADT, skeletal muscle proteomics revealed downregulation of mitochondrial-related pathways, and upregulation of ECM remodeling, immune and inflammatory response, and coagulation pathways. Our findings imply that muscle endurance and mitochondrial function are key indicators of better PROs in patients with advanced PCa starting and during the initial six months of ADT, highlighting mitochondria as a potential therapeutic target.

ALM assessed by DEXA is a validated surrogate measure for muscle mass^22^ and is decreased in PCa patients and with ADT^23^. However, its relationship with different domains of objective physical function and PROs in cancer is not well-established^24,25^. In our current study, ALM correlated with strength but not with endurance before ADT, in agreement with prior reports in older adults without cancer^13^. Regression analysis further supported these findings, showing that only SCP and HGS were positively predicted by ALM, while 6MWT and VO_2_ peak were negatively predicted by fat mass, not ALM. These findings could be explained by differences in fiber type composition, as fatigue-resistant oxidative fibers are related to endurance due to their higher mitochondrial content, while the large fast-twitch fibers contain fewer mitochondria and are associated with strength^26,27^. This will need to be tested in follow-up studies. Interestingly, greater ALM was related to worse patient-reported PF and greater symptom burden, and this remained significant after adjusting for covariates, whereas muscle endurance was positively linked to better PROs. This suggests that muscle endurance may be more clinically relevant than ALM in advanced PCa, as it is associated with better physical function and lower symptom burden. Moreover, this is important to consider when developing therapeutic interventions and selecting primary outcomes in patients with PCa.

Mitochondrial function declines with aging^28^ and is negatively impacted in many diseases, including breast cancer^29^. However, the role of mitochondria in PCa and the effects of ADT on its function remain unclear. Testosterone modulates mitochondrial function, biogenesis, and mitophagy in animals^30^, and circulating testosterone levels in older men without cancer positively correlate with VO_2_ peak and skeletal muscle expression of oxidative phosphorylation genes^31^. While skeletal muscle mitochondrial protein content has been reported in PCa^32^, to our knowledge, this is the first study to investigate skeletal muscle mitochondrial function before and/or during ADT. Here, we report that prior to ADT, ATP Max was negatively associated with BMI, ALM, and fat mass. Similar inverse associations with body weight and fat mass have been reported in older adults without cancer^33,34^. Baseline ATP Max also correlated positively with muscle endurance and PROs. The relationship between endurance and mitochondrial function has been reported in healthy adults^28^ and in patients with heart failure^35^, as endurance relies heavily on oxidative phosphorylation. In contrast, muscle strength is primarily driven by anaerobic respiration, which is mainly independent of mitochondria^36^, and may explain the lack of association between ATP Max and SCP observed here. Ex vivo mitochondrial respiration displayed a moderate correlation with patient-reported PF and ATP Max. Consistent with these findings, when individuals were stratified by median ATP Max, those above the median exhibited lower BMI, fat mass, and HGS, and better performance on the 6MWT and VO_2_ peak, and a trend for better PROs. Interestingly, although state 3 u ex vivo respiration showed limited correlations, categorical analysis revealed that individuals with above the median state 3 u ex vivo had significantly higher ATP Max and more favorable PROs, with a trend toward improved 6MWT performance. These differences may be explained by the reduction in data variability when using categorical analyses, along with the fact that MRS evaluates whole-muscle mitochondrial capacity in vivo^37^, while ex vivo respirometry focuses on oxygen consumption in isolated mitochondria, reflecting individual mitochondrial quality^38,39^. Additionally, MRS examined mitochondrial function in the tibialis anterior, while ex vivo respirometry was performed on the vastus lateralis. These results suggest that mitochondria may play a key role in physical function and PROs for patients with PCa and could serve as a target for improving these PCa-related symptoms. MRS may also act as a non-invasive tool to assess mitochondrial function if the present results are validated in larger studies.

We also explored changes induced by ADT on body composition, physical function (strength and endurance), and PROs across various QOL domains and symptoms. Following six months, we observed a decrease in ALM, an increase in fat mass, and no change in BMI, which aligns with previous reports^40–42^. VO_2_ peak and HGS declined after three months, but showed no further declines at six months, consistent with other studies^23,43^, whereas 6MWT decreased only after six months. This delay may be due to a learning effect^44^ or to the fact that 6MWT is less sensitive to the effects of ADT^45^. Here, we report a trend toward reduced physical activity after ADT, similar to a previous report^23^. SCP remained unchanged, in line with a previous study, which observed that gluteus maximus activation during stair ascent increased during ADT, likely as compensation for reduced quadriceps force, which resulted in SCP maintenance^46^. Consistent with prior findings, changes in FACT-P and QLQ-C30 observed here reflected declines in physical functioning, well-being, and total scores, and increases in fatigue^23,47^. Our results highlight the differential impact of ADT on a range of physical function outcomes, emphasizing the need for selecting appropriate tools in future clinical trials involving PCa patients undergoing ADT.

Key regulators of mitochondrial health include PGC1α and TFAM, which promote mitochondrial biogenesis^17^, NRF1, which mediates nuclear–mitochondrial communication^48^, and BNIP3, PINK1, and PARK2, which play a role in mitophagy and mitochondrial quality control^19,20^. We found that expression of these genes did not significantly change after six months of ADT in skeletal muscle. However, higher baseline expressions of PGC1α, TFAM, BNIP3, and PINK1 were associated with greater VO_2_ peak at baseline and maintenance in 6MWT after ADT, while greater NRF1 was associated with better mitochondrial respiration. These findings further support that mitochondrial function is more closely linked to measures of muscle endurance than to muscle strength or mass in prostate cancer patients.

Although mitochondrial respiration remained unchanged, skeletal muscle proteomic analysis revealed widespread dysregulation of mitochondrial-related pathways after six months of ADT. Proteins most responsible for the enrichment of mitochondrial function pathways were significantly downregulated after 6 months of ADT. We focused mainly on these downregulated genes, as these were directly related to the study’s a priori main goal to characterize the relationship between muscle mass, strength, endurance, PROs, and mitochondrial function. However, GSEA also highlighted upregulation of ECM remodeling, potentially indicating increased muscle fibrosis and activation of inflammation-related pathways. This is consistent with prior findings in hypogonadal men, where testosterone administration reduced serum inflammatory cytokines^49^, and suggests that ADT may promote a pro-inflammatory state. Similar molecular signatures have also been reported recently in a mixed group of pancreatic and advanced colon cancer patients with muscle and weight loss, including activation of immune, extracellular matrix, and coagulation pathways^50^. This pro-inflammatory state and extracellular matrix remodeling phenotype has been further studied in skeletal muscle biopsies of female patients with pancreatic cancer cachexia, where complement activation, immune cell infiltration, and pathological collagen remodeling were observed when compared to non-cancer controls^51^. This suggests that ADT may potentiate cancer-related inflammation and fibrosis in skeletal muscle. When we compared individuals based on their functional performance status during ADT, we observed a more profound downregulation of mitochondrial biogenesis and fatty acid metabolism, combined with upregulation in ECM remodeling, immune response, and coagulation pathways in those with more severe decline, suggesting a greater physiological impact compared to those with better PWB, HGS, VO_2_ peak, higher ALM, and lower fatigue scores. These findings highlight the complex and heterogeneous interplay between clinical outcomes and mitochondria, inflammation, and tissue remodeling in skeletal muscle after ADT.

More granular pathway analysis using IPA revealed broad suppression of multiple subunits within the electron transport chain, suggesting widespread impairment in mitochondrial respiratory capacity following ADT. Although NAD was predicted to be downregulated, mass spectrometry-based quantification showed no significant changes, indicating that impairment of oxidative phosphorylation may occur independently of NAD availability, unlike what is typically seen with aging^52^. These findings suggest that ADT induces mitochondrial dysfunction, highlighting it as a potential therapeutic target. While non-pharmacological interventions such as exercise have demonstrated benefits in preserving mitochondrial health^28^, including in patients undergoing chemotherapy for breast cancer^53^, the type of intervention is crucial, as neuromuscular electrical stimulation, despite inducing fiber hypertrophy, did not preserve contractile function and reduced subsarcolemmal mitochondrial content^54^. This highlights the need for pharmacological interventions, which remain limited. For instance, Elamipretide, a mitochondria-targeted peptide, has demonstrated efficacy in enhancing ADP sensitivity, improving mitochondrial function and muscle performance in models of aging^55^ and cancer-associated muscle wasting^56,57^, supporting its potential to mitigate ADT-induced mitochondrial dysfunction.

Our longitudinal study design also allowed us to identify predictors of increased susceptibility to these side effects of ADT, which may serve as prognostic biomarkers for selecting patients at greater risk for sarcopenia for closer monitoring, tailored exercise prescription, and/or for future therapeutic clinical trials. Baseline VO_2_ peak and ex vivo mitochondrial respiration predicted declines in ALM, muscle function, and PROs. Also, individuals with higher baseline ATP Max showed maintenance in PROs. Previous studies reported that greater comorbidity burden and higher Gleason score are associated with increased fatigue^58^ and declines in self-reported PF^10^ after ADT. In the current study, baseline ALM did not predict any outcomes after ADT. However, higher mitochondrial function at baseline was predictive of maintenance of muscle mass, strength, and patient-reported outcomes during ADT. Assessing mitochondrial function before ADT initiation may help identify individuals at higher risk for muscle mass and function decline, and could guide personalized interventions, such as targeted exercise programs, to preserve muscle health and improve outcomes as those used to counteract functional decline during aging^28^. Future studies should validate these findings in larger cohorts to confirm whether mitochondrial function and endurance can reliably identify PCa patients more susceptible to the side effects of ADT.

When observing the relationship between six-month changes in outcomes of interest, patients with greater appetite loss experienced greater declines in BMI, ALM, and muscle function. While baseline ALM was negatively associated with PROs, its preservation during ADT was associated with maintenance of PROs and muscle function. Maintenance of VO_2_ peak and 6MWT was also associated with smaller declines in PROs. This highlights the complex relationship that exists between muscle mass and function, which will need to be studied further. Our data suggests that a multimodal approach aimed at preserving muscle function and mass might be an effective strategy to mitigate the side effects of ADT, but this will require testing in a randomized controlled trial.

There are several limitations in this study, including a small sample size for mitochondrial function measurements and a limited study timeline of 6 months. While DEXA measures lean mass rather than muscle mass, ALM serves as a reliable surrogate marker, focusing on extremities where muscle tissue predominates. Although correlations cannot establish causation, the longitudinal design allows us to determine the predictive value of these parameters, despite most correlation coefficients being moderate, with few exceeding 0.6. Variability in the mitochondrial measures may have limited our ability to detect a difference. MRS in vivo measurements may have been influenced by incomplete muscle fiber recruitment due to lower exercise tolerance in our older prostate cancer population^59^. Ex vivo respiration measures are known to exhibit variability, even under standardized conditions, due to factors such as oxygen or substrate depletion at high respiratory rates, differences in mitochondrial yield, and inter-assay variability^60^. Although permeabilized myofibers preserve mitochondrial structure and may be more sensitive^61^, measurements in isolated mitochondria allow control of assay conditions and remain a practical, informative approach to study mitochondrial function^60^. We acknowledge that some participants improved after ADT, while others worsened; however, the design of our study does not allow us to establish the reasons for the variability, and future studies are needed to further elucidate the heterogeneous response to ADT. This will be the focus of future studies. Our study is strengthened by the inclusion of a comprehensive phenotyping of muscle strength, endurance, and mass, PROs using validated questionnaires with a focus on those related to muscle mass and function, and an array of mechanistic assessments, including skeletal muscle mitochondrial function (in vivo and ex vivo) and skeletal muscle proteomics at baseline and after six months of ADT. Furthermore, the homogeneous population and state-of-the-art, validated methods enhance the reliability of our findings.

Collectively, the findings from our study implicate mitochondria as key orchestrators of muscle endurance and patient-perceived function in men with advanced PCa about to undergo ADT. Moreover, greater baseline endurance (VO_2_ peak) and mitochondrial function predict smaller declines in ALM and various objective and subjective functional outcomes after six months of ADT and may help select patients and outcomes for clinical trials targeting these outcomes. Future studies should test whether mitochondria may be a suitable target for improving physical function and PROs in PCa.

## Methods

### Ethical approval

This protocol was approved by the Veterans Affairs Puget Sound Health Care System (VAPSHCS) and the University of Washington Institutional Review Boards and their Research and Development Committees and was conducted in compliance with the Declaration of Helsinki and its amendments and the International Conference on Harmonization Guideline for Good Clinical Practices. Informed consent was obtained from all participants prior to their enrollment in the study.

### Study design and subjects

Men with confirmed advanced or metastatic PCa initiating primary ADT for at least six months were recruited from the VAPSHCS or University of Washington Urology Clinics. Exclusion criteria included abnormal liver function (AST or ALT >3× upper limit of normal), renal failure (creatinine >2.5 mg/dL), untreated thyroid disease, class III-IV congestive heart failure, AIDS, other cancer diagnosed within the past five years other than non-melanoma skin cancer, severe chronic obstructive pulmonary disorder requiring home O_2_, chronic uncontrolled hypertension, active/uncontrolled infection, recent cardiovascular event (myocardial infarction, cerebrovascular accident, arrhythmias or unstable angina), uncontrolled diabetes (HbA1c > 9%), underlying muscular or neuromuscular disorder or neurological deficit contributing to sarcopenia, prior ADT, current use of an investigational agent or testosterone, high dose steroids (equivalent to ≥20 mg prednisone/day), or recent megestrol for anorexia. All participants reported to the VAPSHCS prior to initiation of ADT (baseline), and then three months and six months after starting ADT for study assessments. Tumor response was evaluated through review of clinical records, including but not limited to urology and oncology notes, imaging and laboratory values, subject interviews, and, when clinically indicated, a physical exam at the three- and six-month follow-up visit. Specifically, when available, specific markers such as prostate-specific antigen or alkaline phosphatase were extracted from the medical record to evaluate tumor progression, lack of response to therapy, or metastatic disease. Other laboratory values, such as hepatic and renal function, were collected as well. Radiological reports (including CT, MRI) were reviewed for documentation of new or progressing metastatic disease, such as to the bones or lymph nodes, interval growth of known lesions, or radiographic progression. Moreover, clinical notes from oncology and/or urology visits were reviewed for documentation of disease-related symptom progression, changes in treatment strategy due to suspected progression, or physician-assessed treatment response.

### Study measures

For this longitudinal observational study, participants reported to the VAPSHCS in the morning after fasting overnight. Assessments of body composition, physical function, PROs, and muscle biopsies from the vastus lateralis muscle were performed to measure mitochondrial respiration and for proteomic analysis. All outcomes were measured at the baseline, 3- and 6-month visits except muscle biopsies, which were obtained only at baseline and 6 months.

### Body composition

DEXA was used to measure total lean mass, ALM, total fat mass, and total percent fat. Whole-body scans were performed and analyzed using standard procedures as previously performed^62,63^.

### Muscle function

Hand grip strength (HGS) was evaluated via handheld dynamometer (Jamar Hydraulic Dynamometer, J.A. Preston Corp., Clifton, NJ)^64^. To measure stair climb power (SCP), participants climbed a flight of standard stairs (13 steps, 15.3 cm each) at the highest possible speed^63^. The shortest time between 2 and 3 trials was used to calculate power (Watts): body mass (kg) × acceleration of gravity (9.81 m/s^2^) × vertical distance (1.99 m)/time (seconds). For the six-minute walk test (6MWT), participants walked back and forth down a hallway (30-m) for six minutes, and the total distance was recorded^64^. Peak aerobic capacity (VO_2_ peak) was measured by collecting expired gases while the participant pedaled a cycle ergometer at increasing workloads until they stopped due to fatigue or could not maintain a speed of 55 rpm^65^. VO_2_ peak has shown to have excellent test-retest variability in patients with cardiac or respiratory disease^66^, with coronary artery disease^67^, and in men with prostate cancer with moderate within-subject variability^68^. Similarly, 6MWT^69^, HGS^70^, and SCP^71^ have also shown high test-retest reliability, supporting the reliability of these functional measures. Accelerometry was captured over seven days following each study visit, during which participants wore an Actical activity monitor (Philips Respironics, Murrysville, PA) on the wrist, even during sleep. Data from wear periods of less than three days or under ten hours per day of recorded activity were excluded, as in prior studies^72,73^. Average daily time spent in moderate and vigorous activity (non-sedentary) was analyzed.

### Patient-reported outcomes

The FACT-P^74^ and EORTC QLQ-C30^75^ are both well-validated questionnaires in PCa. The FACT-P measures PWB, FWB, Emotional (EWB), Social/Family (SFWB) Well-Being, and PCa AC^76^, with higher scores indicating better QOL. EORTC QLQ-C30 assesses five functional scales (PF, Role Limitations (RF), Cognitive (CF), Emotional (EF), and Social (SF)), nine symptom/problem scales, and a global health status/QOL scale, where higher functional and QOL scores indicate better status, and higher symptom scores indicate worse symptoms.

### Ex vivo mitochondrial respiration

Muscle biopsies were performed on the vastus lateralis under sterile conditions using a sterilized biopsy needle. Following local anesthesia with lidocaine and a small incision, approximately four sections of muscle tissue were obtained, yielding enough for mitochondrial respiration measurement assessed in isolated mitochondria and for proteomic analysis. Muscle biopsies were immediately immersed in mitochondrial isolation buffer (MIB) (210 mM sucrose, 2 mM EGTA, 40 mM NaCl, 30 mM HEPES, pH 7.4) on ice. The muscle was then homogenized in MIB with a Kimble homogenizer and centrifuged at 900 × *g* and 4 °C for 10 min. The supernatant was collected and centrifuged at 10,000 × *g* and 4 °C for 10 min. The supernatant was then discarded, and the pellet was resuspended in MIB. Protein concentration was identified by Pierce Rapid Gold BCA Protein Assay (Thermo Fisher Scientific). After centrifuging the sample one more time at 10,000 × *g* and 4 °C for 10 min, isolated mitochondria were resuspended in mitochondrial assay solution (MAS) (70 mM sucrose, 220 mM d-mannitol, 10 mM KH2PO4, 5 mM MgCl2, 2 mM HEPES, 1 mM EGTA, 0.2% fatty acid-free BSA, pH 7.4; Sigma-Aldrich, Carlsbad, CA, USA) with substrates (5 mM malate and 5 mM pyruvate).

Mitochondrial respiration was measured with the Agilent Seahorse XFe24 Analyzer (Agilent Technologies, Santa Clara, CA) as previously described with a few modifications^39,77,78^. The isolated mitochondria in MAS with pyruvate and malate were plated in Agilent Seahorse XF24 Cell Culture Microplates (7.5 µg/well in triplicate). To assess state 2, state 3, state 4, and state 3u respiration^60^, 50 μl of adenosine diphosphate (ADP, 2 mM), 55 μL oligomycin (2 μM), 60 μL carbonyl cyanide-p-trifluoromethoxy-phenylhydrazone (FCCP, 4 μM), and 65 μL of antimycin A (2 μM) were sequentially loaded into the cartridge plate and injected into the cell plate. Oxygen consumption rate (OCR; pmol/min) of each respiration state was measured using an XFe24 Seahorse Analyzer (Agilent Technologies) in real-time. OCR reflects mitochondrial oxidative phosphorylation capacity of isolated mitochondria, providing insight into mitochondrial quality rather than whole-muscle oxidative capacity. A decline in OCR indicates impaired mitochondrial respiration, which is associated with diminished muscle energy production and is linked to lower exercise capacity and muscle function. This has been demonstrated in aging populations where reduced mitochondrial function is associated with poor gait stability, reduced muscle strength, and diminished physical performance^28^.

### In vivo mitochondrial respiration

Magnetic resonance spectroscopy was used to measure mitochondrial oxidative phosphorylation capacity (ATP Max) of the tibialis anterior as described previously (TA)^37,79^. Briefly, ^31^P spectra were acquired on a 4.7 T Bruker magnet using a surface coil placed over the belly of the tibialis anterior. A short exercise (20–30 s) involving dorsiflexion of the foot was used to reduce the concentration of phosphocreatine (PCr) in the muscle by approximately 30–50% from the resting state while maintaining muscle pH above 6.8. The PCr recovery was measured for six minutes. The PCr recovery was fit with a monoexponential function to determine a time constant for recovery (inverse of the rate constant). The maximal ATP production (ATP Max) was calculated by dividing the resting concentration of PCr in the muscle by the time constant of recovery, where the resting level of PCr was 24.5 mM. ATP Max provides a non-invasive estimate of whole-muscle mitochondrial oxidative capacity. This recovery reflects the efficiency of ATP production through oxidative phosphorylation. Clinically, a reduced ATP Max indicates mitochondrial dysfunction and has been observed in age-related conditions such as sarcopenia^28^ and frailty^80^.

### Real-time reverse transcription-quantitative polymerase chain reaction (RT-PCR)

Total RNA was isolated from 10–20 mg of muscle biopsies from 10 subjects at baseline and six months using the Qiagen RNeasy fibrous tissue mini kit (Qiagen, Hilden, Germany). Transcription levels of the isolated RNA were identified by BioTek Cytation 5. Total RNA was reverse transcribed to cDNA by QuantiTect Reverse Transcription Kit (Qiagen, Hilden, Germany). RT-PCR was detected by an ABI 7500 instrument (Applied Biosystems, Foster City, CA) by using predesigned Taqman Expression Assays (Thermo Fisher Scientific, Waltham, MA). The quantification of genes of interest was normalized to a reference gene glyceraldehyde-3-phosphate dehydrogenase (GAPDH, Hs02786624_g1) and expressed as relative fold-change of the baseline group by a standard 2-ΔCT method. One subject was excluded from the analysis because the CT value at the six-month visit was outside the acceptable range. The following Taqman primers from Thermo Fisher Scientific were used in this study: PPARGC1A (Hs00173304_m1), TFAM (Hs00273372_s1), NRF1 (Hs00602161_m1), BNIP3 (Hs00969291_m1), PINK1 (Hs00260868_m1), and PARK2 (Hs01038322_m1).

### Proteomics of skeletal muscle

Muscle biopsies were resuspended in 1% sodium deoxycholate (SDC) in 100 mM ammonium bicarbonate buffer (SDC buffer) and incubated for 15 min on ice. The sample was quickly centrifuged (3200 rcf (relative centrifugal force) for 15 min) and was transferred to a 96-well round-bottom microtiter plate. An additional 100 µL of SDC buffer was added to each sample, and the tissue was extracted by sonication on PIXUL (Matchstick Technologies, Seattle, WA). The plate was centrifuged at 3200 rcf for 30 min at 4 °C, and 200 µL of the supernatant was transferred to 1.5 mL low-binding tubes. The tubes were further centrifuged at 21,000 rcf for 20 min at 4 °C, and 150 µL was transferred to a PCR plate. Protein concentration was determined using the BCA assay.

For trypsin digestion, 10 µg of the protein was diluted in the SDC buffer containing 5% acetonitrile, reduced with DTT (Cf = 5 mM), and incubated for 1 h at 90 °C. The sample was then alkylated with iodoacetamide (Cf = 20 mM) for 45 min in the dark, and excess reagent was quenched with an additional aliquot of DTT. The sample was digested with 1 µg of trypsin/Lys-C (Promega, WI) overnight at 37 °C with mixing. SDC was precipitated with 4 µL of trifluoroacetic acid, and the supernatant was collected and desalted on the Oasis HLB Elute plate, dried down, and reconstituted in 33 µL of 1%acetonitrile/0.1% formic acid (0.2 µg/µL).

The abundance of selected proteins was quantified by mass spectrometry using data-independent analysis (DIA) LC-MS/MS. A total of 1 µg of digested proteins was injected on an LC-MSMS consisting of a neoVanquish (Thermo Scientific) and a Thermo Orbitrap Exploris480 (Thermo Fisher, San Jose, CA) mass spectrometer with electrospray ionization. After desalting on a PepMap Neo Trap Cartridge (Thermo Scientific) (flow rate 60 μL/min), the digested peptides were separated on an analytical column (Reprosil-Pur 120 C18-AQ, 5 µm, 250 × 0.075 mm, Dr. Maisch HPLC GmbH). The following multi-step linear gradient was used: 1–5%B in 2 min, 5–25% in 50 min, 25–35% in 10 min. At the end of the gradient column was washed with a ramp to 80%B and re-equilibrated (A - 0.1% formic acid in water, B - acetonitrile, 0.1% formic acid, flow rate of 0.4 µL/min).

Data-independent analysis parameters were as follows: MS1 scan (400–700 Da, resolution 120,000, maximum injection time 50 ms) followed by 60 MS/MS scans across a 430–670 Da range with 4 Da mass selection window each (resolution 15,000, maximum injection time 22 ms, loop time 3 s). Fragmentation was induced by HCD activation at a normalized collision energy 30%. Further data processing was accomplished using Skyline^81^ to extract fragment ion chromatograms of the MS2 scans with 10 ppm accuracy windows.

Proteins were identified and quantified through DIA-GPF analysis of a pooled sample. DIA data was acquired by repeated injection of the sample with overlapped 4 m/z selection windows (offset 2 m/z) over the m/z ranges 400–500, 500–600, 600–700. Peptide and protein identification was accomplished using MS-Fragger within FragPipe (v 21.1)^82^ database search (Human Uniprot Reference database supplemented with common contaminants, downloaded Dec 12, 2022) using DIA_Speclib_Quant workflow with strict tryptic specificity allowing for 1 missed cleavage, fixed alkylation on Cys residue, and variable Met oxidation and acetylation at the protein N-termini, with 20 ppm accuracy for both precursors and fragments, isotope error set to 0/1/2, and peptide and protein validation at 0.01 FDR. Proteins were then quantified across all samples using DIA-NN (version 1.8.2 beta 27)^83^ with mass accuracy and scan windows optimized and inferred separately for different runs and FDR 0.01.

Prior to analysis, two outlier subjects were removed along with all proteins lacking valid gene symbols, and any proteins missing more than 50% of the values in both the baseline and 6-month timepoints were removed. We imputed the remaining missing values in the log_2_ domain as a random value between 50 and 100% of the minimum.

Following a validated methodology^84^, a paired DESeq2^85^ analysis was performed in the R environment after appropriate scaling (log2[protein abundance] × 1000) to compare differential protein abundances between baseline and 6 months. The DESeq2 statistic was used to pre-rank the proteins for Gene Set Enrichment Analysis (GSEA)^86^. Using Hallmark and Canonical Pathways databases from Molecular Signature Databases, we determined pathways with significant enrichment using an FDR < 0.05 threshold. We used Cytoscape (v3.8.0)^87^ to link enriched GSEA gene sets based on overlapping proteins (>50%) and create a modular network of the proteomic response to ADT. We extracted leading-edge proteins from significantly enriched gene sets involved in mitochondrial processes and normalized their scaled abundances via z-score to create baseline and 6-month visit mitochondrial protein expression scores. Baseline and 6-month protein abundances were compared using a paired *t*-test. We leveraged Ingenuity Pathway Analysis’s (IPA) knowledge base to map these proteins to functional mitochondrial units (Complexes I–V).

For each of 5 clinical outcomes (ALM, fatigue, HGS, PWB, and VO_2_ Peak), participants were grouped as below or above the median change based on absolute outcome changes, with those above the median implying a worse physiological change. Paired DESeq2 and GSEA analyses were run as previously described to investigate enriched pathways for each group.

### Statistical analysis

Statistical analysis was performed with SPSS version 29.0.1.0 (SPSS, Inc., Chicago, IL) except for proteomic analyses. The primary analysis set a priori assessed changes over time in body composition, physical function, PROs, and mitochondrial function using generalized estimating equations (GEE) with an exchangeable correlation matrix, adjusting for baseline values (reference), tumor stage, and age. Regression coefficients (β) and 95% confidence intervals reflect within-subject changes across timepoints (baseline, 3 months, and 6 months). This method accounts for the repeated measures structure of the data and accommodates partially missing data without requiring imputation. The Kolmogorov–Smirnov test assessed normality, and non-normally distributed variables were log-transformed. For QLQ-C30 variables, the transformation log (x + 1) was applied to account for symptom scores equal to zero, which otherwise could not be log-transformed. Secondary analyses, also set a priori, included Spearman and Pearson correlations for non-normally and normally distributed data, respectively, to assess baseline associations. Correlations with change variables (6-month minus baseline) were analyzed using partial correlations, adjusting for baseline values when significant associations were present at baseline. Stepwise multivariable regression to identify independent predictors of outcome measures and non-parametric *t*-tests (Mann–Whitney U) for between-group comparisons when normality assumptions were not met were also used. Statistical significance was defined as two-sided, α ≤ 0.05. No data was excluded from analysis, except where specifically noted.

### Reporting summary

Further information on research design is available in the Nature Portfolio Reporting Summary linked to this article.

## Supplementary information

Supplementary Information

Description of Additional Supplementary Files

Supplementary Data 1

Reporting Summary

Transparent Peer Review file

## Source data

Source Data File

## Data Availability

The proteomics data generated during the study has been deposited in the PRIDE partner repository of the ProteomeXchange Consortium under the accession code PXD061070. A Source Data File containing the raw data of the figures and tables in this manuscript and supplementary data is provided with the paper. Source data are provided with this paper.
