# Supplementary material for: Muscle mitochondria, function, mass, and quality of life in prostate cancer during androgen deprivation therapy
Source: Nat Commun. 2026 May 27;17:6884. doi: 10.1038/s41467-026-73542-x (PMC13389078; doi:10.1038/s41467-026-73542-x)
Supplement: Supplementary file 1 — Supplementary Information [file 41467_2026_73542_MOESM1_ESM.pdf]

Supplemental Figures and Tables

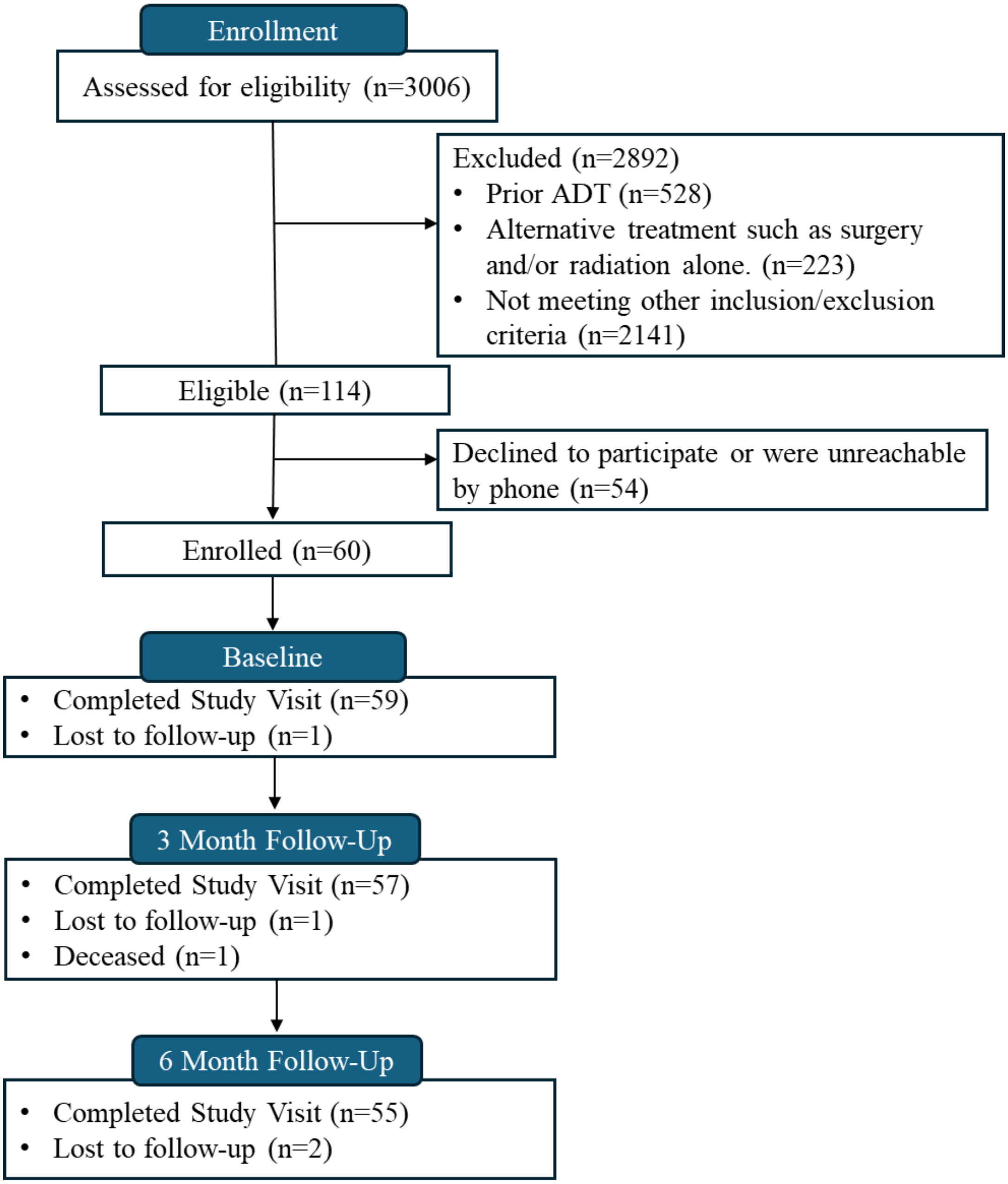

Supplemental Figure 1. Consort Diagram. ADT, androgen deprivation therapy.

				Muscle Endurance			Muscle Strength		FACT-P				EORTC QLQ C-30					
	BMI	ALM	Fat mass	VO_2 Peak	6MWT	Physical Activity	SCP	HGS	PWB	FWB	PCa AC	Total	QOL	PF	RF	SF	Fatigue	Dyspnea
BMI		.64**	.87**	-.33*				.29*	-.27*		-.26*			-.28*	-.32*	-.29*		.30*
ALM			.48**				.34*	.36**	-.42**		-.28*			-.26*	-.27*	-.44**	.33*	.36**
Fat Mass				-.39**											-.26*			
VO_2 Peak					.69**	.43**	.38**	.31*	.42**	.28*	.36**	.28*	.44**	.57**	.43**	.43**	-.38**	-.39**
6MWT						.59**	.59**	.38**	.47**	.26*	.36**	.26*	.43**	.66**	.47**	.42**	-.48**	-.47**
Physical Activity							.49**	.37**	.29*		.29*			.38**			-.36**	
SCP								.53**						.36**				
HGS																		
State 3u[A]														.32*				
Maximum ATP[B]	-.39*	-.41*	-.53**	.57**	.57**	.40*		-.37*	.44*				.42*	.42*	.54**	.42*	-.41*	

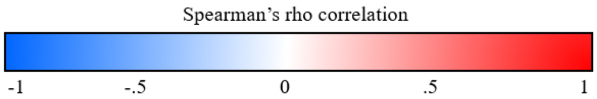

Supplemental Figure 2. Baseline correlations between body composition, physical function, patient reported outcomes, and mitochondrial function. [A] *Ex vivo* respirometry [B] Magnetic resonance spectroscopy. Associations were evaluated using two-sided Spearman correlations. No multiple-comparison adjustments were applied. Abbreviations: BMI, body mass index; ALM, appendicular lean mass; 6MWT, six-minute walk test; SCP, stair climb power; HGS, hand grip strength; FACT-P, Functional Assessment of Cancer Therapy-Prostate; PWB, physical well-being; FWB, functional well-being; PCa AC, prostate cancer additional concerns; EORTC QLQ-C30 , European Organization for Research and Treatment of Cancer Quality of Life Questionnaire Core 30; QOL, quality of life; PF, physical functioning; RF, role functioning; SF, social functioning. * $p < 0.05$, ** $p< 0.01$.

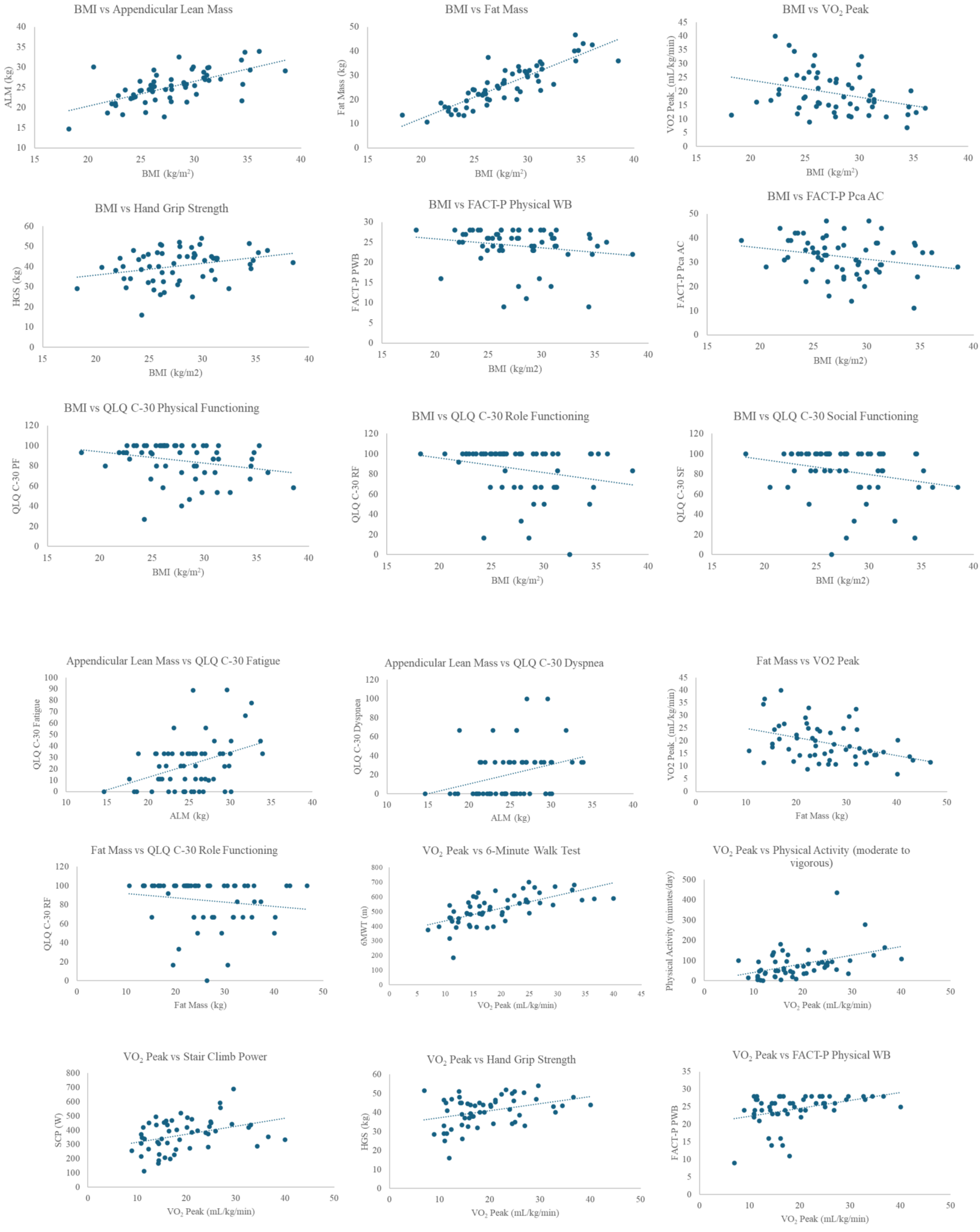

BMI vs Appendicular Lean Mass
ALM (kg)
BMI (kg/m²)
BMI vs Fat Mass
Fat Mass (kg)
BMI (kg/m²)
BMI vs VO₂ Peak
VO2 Peak_(mL/kg/min)
BMI (kg/m²)
BMI vs Hand Grip Strength
HGS (kg)
BMI (kg/m2)
BMI vs FACT-P Physical WB
FACT-P PWB
BMI (kg/m2)
BMI vs FACT-P Pca AC
FACT-P Pca AC
BMI (kg/m2)
BMI vs QLQ C-30 Physical Functioning
QLQ C-30 PF
BMI (kg/m²)
BMI vs QLQ C-30 Role Functioning
QLQ C-30 RF
BMI (kg/m²)
BMI vs QLQ C-30 Social Functioning
QLQ C-30 SF
BMI (kg/m2)
Appendicular Lean Mass vs QLQ C-30 Fatigue
QLQ C-30 Fatigue
ALM (kg)
Appendicular Lean Mass vs QLQ C-30 Dyspnea
QLQ C-30 Dyspnea
ALM (kg)
Fat Mass vs VO2 Peak
VO2 Peak_(mL/kg/min)
Fat Mass (kg)
Fat Mass vs QLQ C-30 Role Functioning
QLQ C-30 RF
Fat Mass (kg)
VO₂ Peak vs 6-Minute Walk Test
6MWT (m)
VO₂ Peak (mL/kg/min)
VO₂ Peak vs Physical Activity (moderate to vigorous)
Physical Activity (minutes/day)
VO₂ Peak (mL/kg/min)
VO₂ Peak vs Stair Climb Power
SCP (W)
VO₂ Peak (mL/kg/min)
VO₂ Peak vs Hand Grip Strength
HGS (kg)
VO₂ Peak (mL/kg/min)
VO₂ Peak vs FACT-P Physical WB
FACT-P PWB
VO₂ Peak (mL/kg/min)

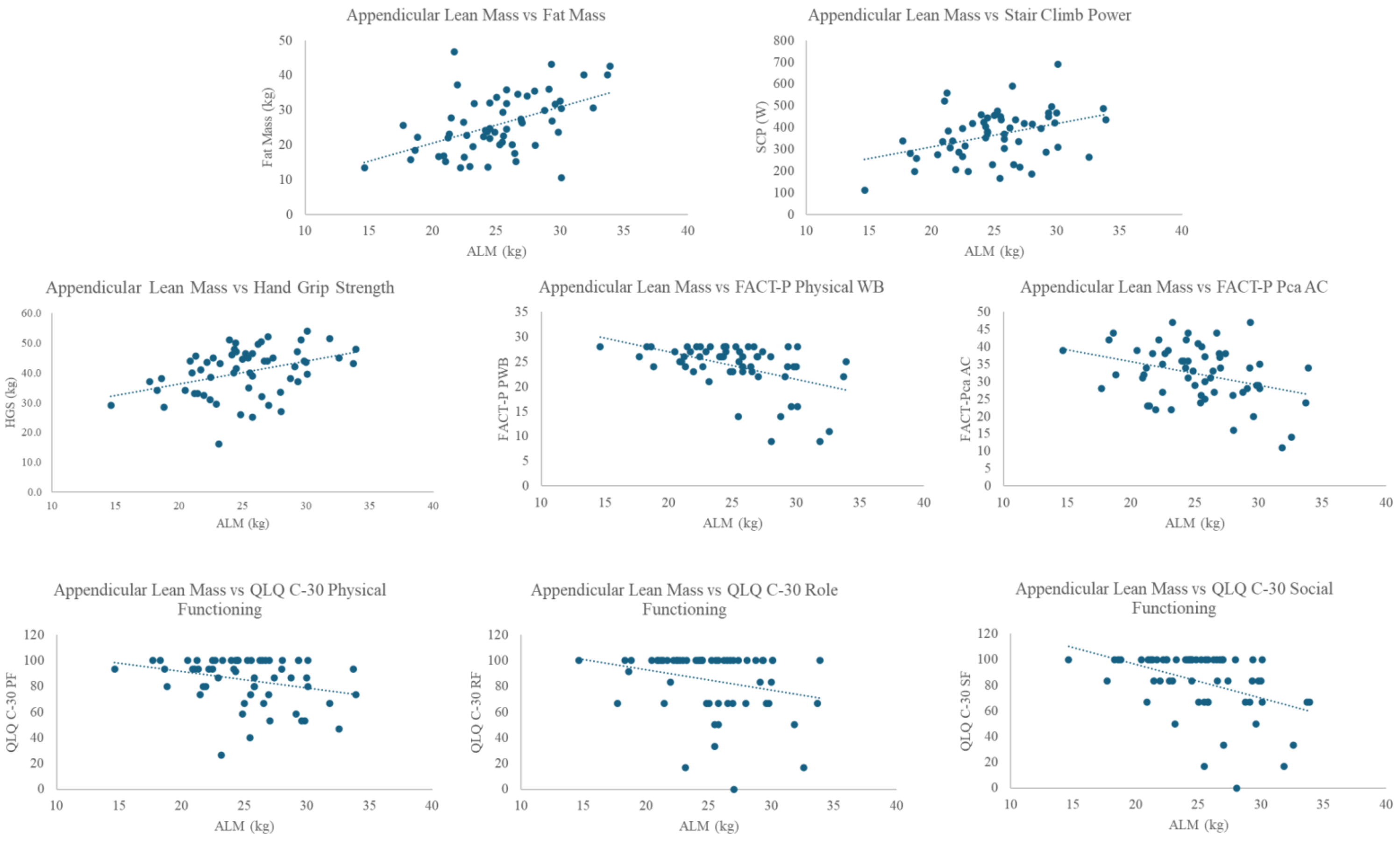
Appendicular Lean Mass vs Fat Mass
Fat Mass (kg)
ALM (kg)
Appendicular Lean Mass vs Stair Climb Power
SCP (W)
ALM (kg)
Appendicular Lean Mass vs Hand Grip Strength
HGS (kg)
ALM (kg)
Appendicular Lean Mass vs FACT-P Physical WB
FACT-P PWB
ALM (kg)
Appendicular Lean Mass vs FACT-P Pca AC
FACT-Pca AC
ALM (kg)
Appendicular Lean Mass vs QLQ C-30 Physical Functioning
QLQ C-30 PF
ALM (kg)
Appendicular Lean Mass vs QLQ C-30 Role Functioning
QLQ C-30 RF
ALM (kg)
Appendicular Lean Mass vs QLQ C-30 Social Functioning
QLQ C-30 SF
ALM (kg)

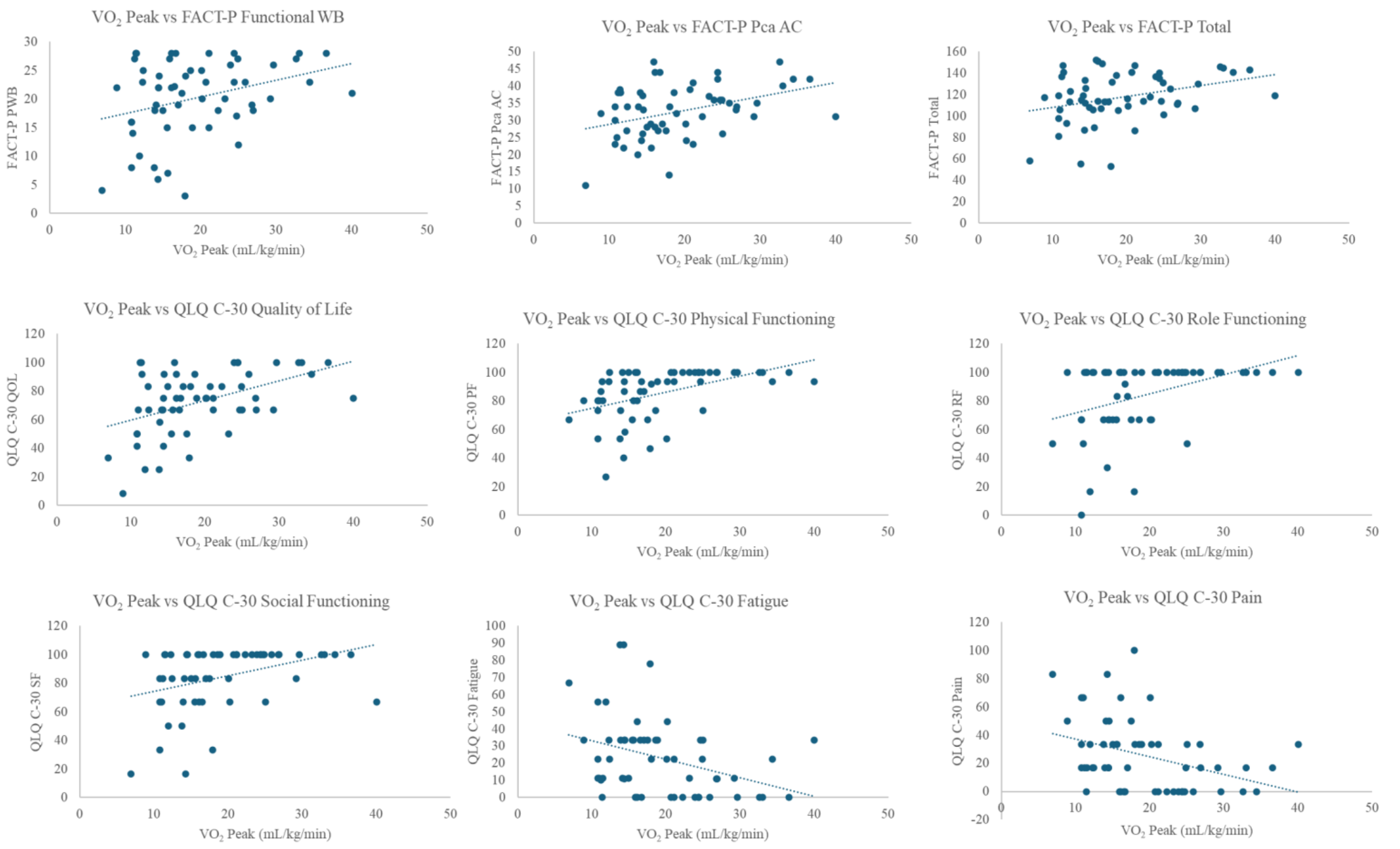
VO2 Peak vs FACT-P Functional WB
FACT-P PWB
VO2 Peak (mL/kg/min)
VO2 Peak vs FACT-P Pca AC
FACT-P Pca AC
VO2 Peak (mL/kg/min)
VO2 Peak vs FACT-P Total
FACT-P Total
VO2 Peak (mL/kg/min)
VO2 Peak vs QLQ C-30 Quality of Life
QLQ C-30 QOL
VO2 Peak (mL/kg/min)
VO2 Peak vs QLQ C-30 Physical Functioning
QLQ C-30 PF
VO2 Peak (mL/kg/min)
VO2 Peak vs QLQ C-30 Role Functioning
QLQ C-30 RF
VO2 Peak (mL/kg/min)
VO2 Peak vs QLQ C-30 Social Functioning
QLQ C-30 SF
VO2 Peak (mL/kg/min)
VO2 Peak vs QLQ C-30 Fatigue
QLQ C-30 Fatigue
VO2 Peak (mL/kg/min)
VO2 Peak vs QLQ C-30 Pain
QLQ C-30 Pain
VO2 Peak (mL/kg/min)

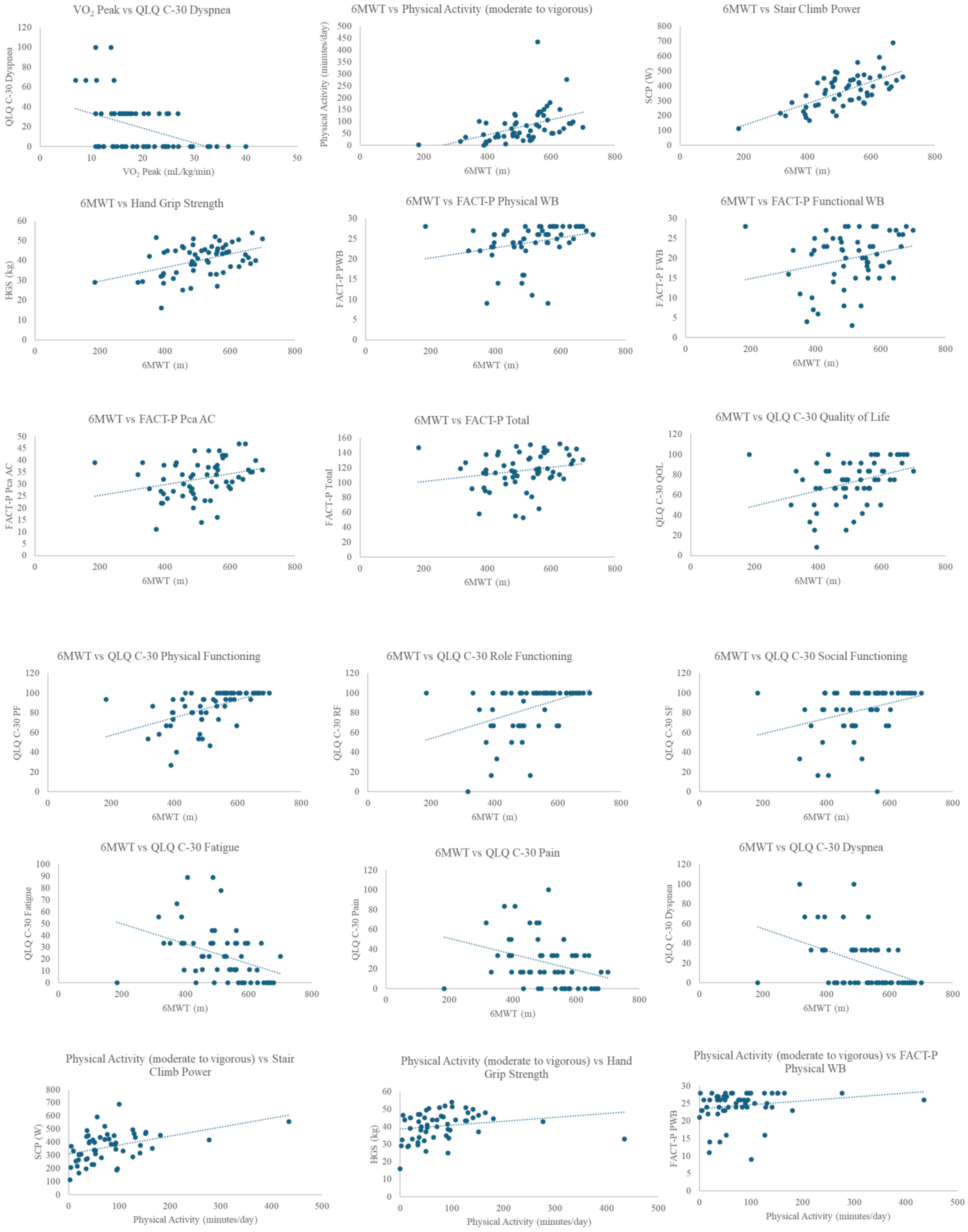

VO2 Peak vs QLQ C-30 Dyspnea
QLQ C-30 Dyspnea
VO2 Peak (mL/kg/min)
6MWT vs Physical Activity (moderate to vigorous)
Physical Activity (minutes/day)
6MWT (m)
6MWT vs Stair Climb Power
SCP (W)
6MWT (m)
6MWT vs Hand Grip Strength
HGS (kg)
6MWT (m)
6MWT vs FACT-P Physical WB
FACT-P PWB
6MWT (m)
6MWT vs FACT-P Functional WB
FACT-P FWB
6MWT (m)
6MWT vs FACT-P Pca AC
FACT-P Pca AC
6MWT (m)
6MWT vs FACT-P Total
FACT-P Total
6MWT (m)
6MWT vs QLQ C-30 Quality of Life
QLQ C-30 QOL
6MWT (m)
6MWT vs QLQ C-30 Physical Functioning
QLQ C-30 PF
6MWT (m)
6MWT vs QLQ C-30 Role Functioning
QLQ C-30 RF
6MWT (m)
6MWT vs QLQ C-30 Social Functioning
QLQ C-30 SF
6MWT (m)
6MWT vs QLQ C-30 Fatigue
QLQ C-30 Fatigue
6MWT (m)
6MWT vs QLQ C-30 Pain
QLQ C-30 Pain
6MWT (m)
6MWT vs QLQ C-30 Dyspnea
QLQ C-30 Dyspnea
6MWT (m)
Physical Activity (moderate to vigorous) vs Stair Climb Power
SCP (W)
Physical Activity (minutes/day)
Physical Activity (moderate to vigorous) vs Hand Grip Strength
HGS (kg)
Physical Activity (minutes/day)
Physical Activity (moderate to vigorous) vs FACT-P Physical WB
FACT-P PWB
Physical Activity (minutes/day)

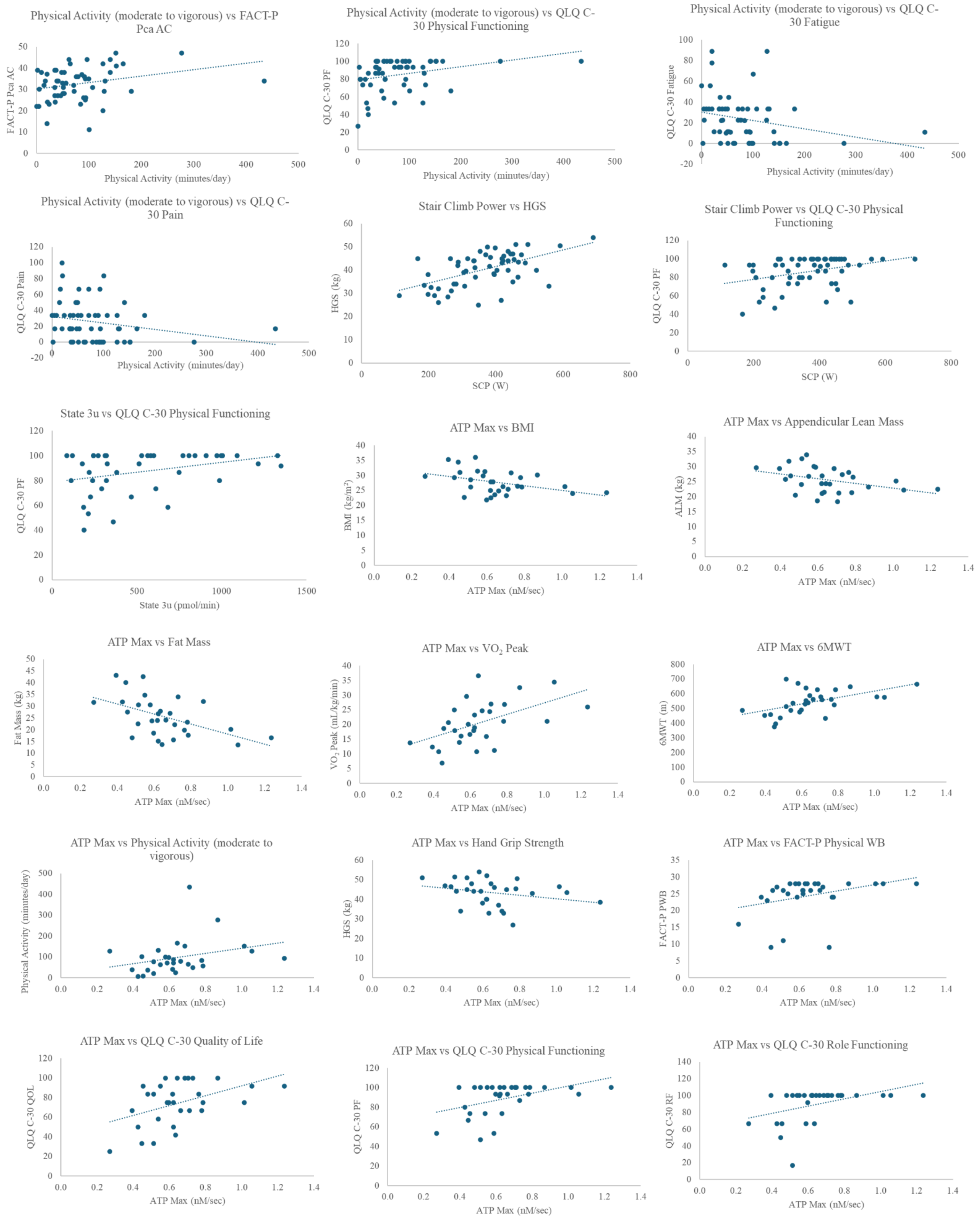
Physical Activity (moderate to vigorous) vs FACT-P Pca AC
FACT-P Pca AC
Physical Activity (minutes/day)
Physical Activity (moderate to vigorous) vs QLQ C-30 Physical Functioning
QLQ C-30 PF
Physical Activity (minutes/day)
Physical Activity (moderate to vigorous) vs QLQ C-30 Fatigue
QLQ C-30 Fatigue
Physical Activity (minutes/day)
Physical Activity (moderate to vigorous) vs QLQ C-30 Pain
QLQ C-30 Pain
Physical Activity (minutes/day)
Stair Climb Power vs HGS
HGS (kg)
SCP (W)
Stair Climb Power vs QLQ C-30 Physical Functioning
QLQ C-30 PF
SCP (W)
State 3u vs QLQ C-30 Physical Functioning
QLQ C-30 PF
State 3u (pmol/min)
ATP Max vs BMI
BMI (kg/m2)
ATP Max (nM/sec)
ATP Max vs Appendicular Lean Mass
ALM (kg)
ATP Max (nM/sec)
ATP Max vs Fat Mass
Fat Mass (kg)
ATP Max (nM/sec)
ATP Max vs VO2 Peak
VO2 Peak (mL/kg/min)
ATP Max (nM/sec)
ATP Max vs 6MWT
6MWT (m)
ATP Max (nM/sec)
ATP Max vs Physical Activity (moderate to vigorous)
Physical Activity (minutes/day)
ATP Max (nM/sec)
ATP Max vs Hand Grip Strength
HGS (kg)
ATP Max (nM/sec)
ATP Max vs FACT-P Physical WB
FACT-P PWB
ATP Max (nM/sec)
ATP Max vs QLQ C-30 Quality of Life
QLQ C-30 QOL
ATP Max (nM/sec)
ATP Max vs QLQ C-30 Physical Functioning
QLQ C-30 PF
ATP Max (nM/sec)
ATP Max vs QLQ C-30 Role Functioning
QLQ C-30 RF
ATP Max (nM/sec)

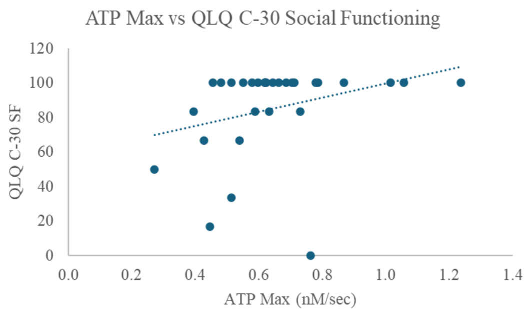

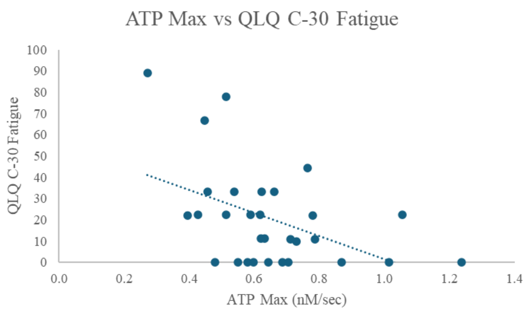

Supplemental Figure 3. Scatter plots illustrating significant baseline correlations between variables presented in Supplemental Figure 2.

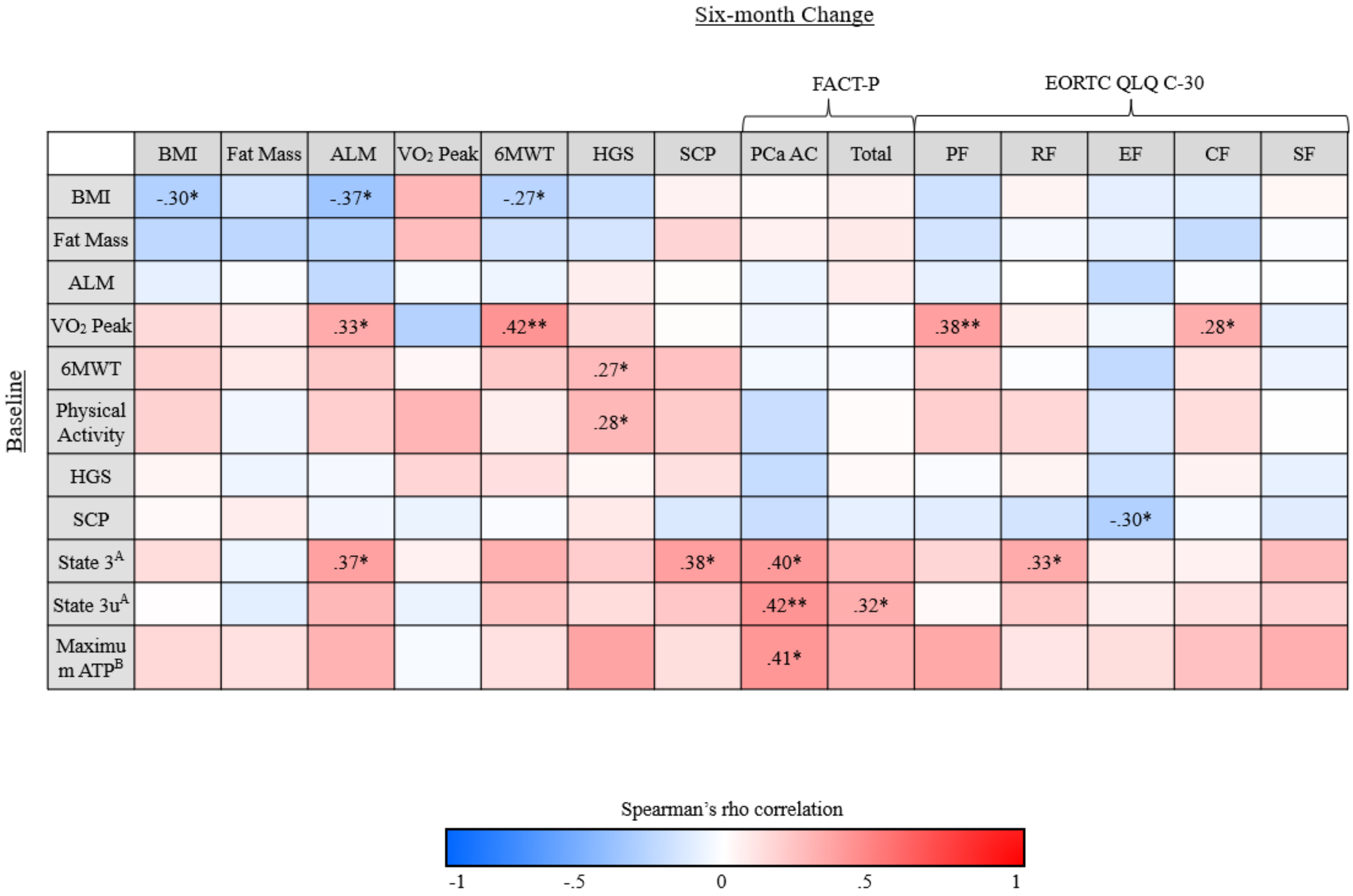

Supplemental Figure 4. Predictors of ADT induced side effects. Percentage change was used for correlations in body composition, physical function, and mitochondrial function while absolute values were used for patient-reported outcomes. Association between baseline values and 6-month changes were assessed using two-sided Spearman correlation analysis. For pairs showing significant associations, partial Spearman correlations were subsequently performed, adjusting for the corresponding baseline variable—provided those variables were also significantly correlated at baseline. No adjustments were made for multiple comparisons. [A] *Ex vivo* respirometry [B] Magnetic resonance spectroscopy measurements. Abbreviations: BMI, body mass index; ALM, appendicular lean mass; 6MWT, six-minute walk test; HGS, hand grip

strength; SCP, stair climb power; FACT-P, Functional Assessment of Cancer Therapy-Prostate; PCa AC, prostate cancer additional concerns; EORTC QLQ-C30, European Organization for Research and Treatment of Cancer Quality of Life Questionnaire Core 30; PF, physical functioning; RF, role functioning; EF, emotional functioning; CF, cognitive functioning; SF, social functioning. * $p < 0.05$, ** $p < 0.01$.

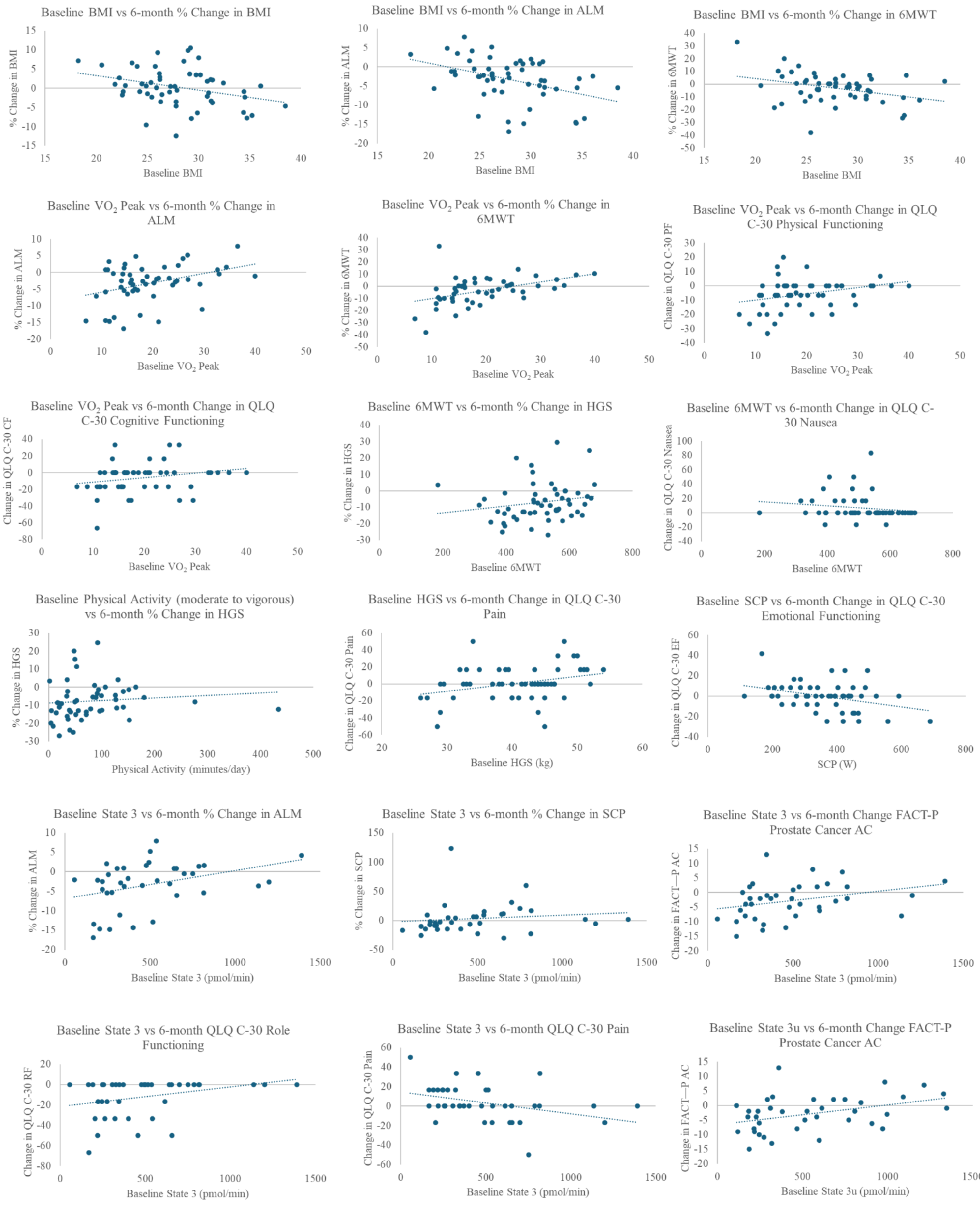
Baseline BMI vs 6-month % Change in BMI
% Change in BMI
Baseline BMI
Baseline BMI vs 6-month % Change in ALM
% Change in ALM
Baseline BMI
Baseline BMI vs 6-month % Change in 6MWT
% Change in 6MWT
Baseline BMI
Baseline VO2 Peak vs 6-month % Change in ALM
% Change in ALM
Baseline VO2 Peak
Baseline VO2 Peak vs 6-month % Change in 6MWT
% Change in 6MWT
Baseline VO2 Peak
Baseline VO2 Peak vs 6-month Change in QLQ C-30 Physical Functioning
Change in QLQ C-30 PF
Baseline VO2 Peak
Baseline VO2 Peak vs 6-month Change in QLQ C-30 Cognitive Functioning
Change in QLQ C-30 CF
Baseline VO2 Peak
Baseline 6MWT vs 6-month % Change in HGS
% Change in HGS
Baseline 6MWT
Baseline 6MWT vs 6-month Change in QLQ C-30 Nausea
Change in QLQ C-30 Nausea
Baseline 6MWT
Baseline Physical Activity (moderate to vigorous) vs 6-month % Change in HGS
% Change in HGS
Physical Activity (minutes/day)
Baseline HGS vs 6-month Change in QLQ C-30 Pain
Change in QLQ C-30 Pain
Baseline HGS (kg)
Baseline SCP vs 6-month Change in QLQ C-30 Emotional Functioning
Change in QLQ C-30 EF
SCP (W)
Baseline State 3 vs 6-month % Change in ALM
% Change in ALM
Baseline State 3 (pmol/min)
Baseline State 3 vs 6-month % Change in SCP
% Change in SCP
Baseline State 3 (pmol/min)
Baseline State 3 vs 6-month Change FACT-P Prostate Cancer AC
Change in FACT—P AC
Baseline State 3 (pmol/min)
Baseline State 3 vs 6-month QLQ C-30 Role Functioning
Change in QLQ C-30 RF
Baseline State 3 (pmol/min)
Baseline State 3 vs 6-month QLQ C-30 Pain
Change in QLQ C-30 Pain
Baseline State 3 (pmol/min)
Baseline State 3u vs 6-month Change FACT-P Prostate Cancer AC
Change in FACT—P AC
Baseline State 3u (pmol/min)

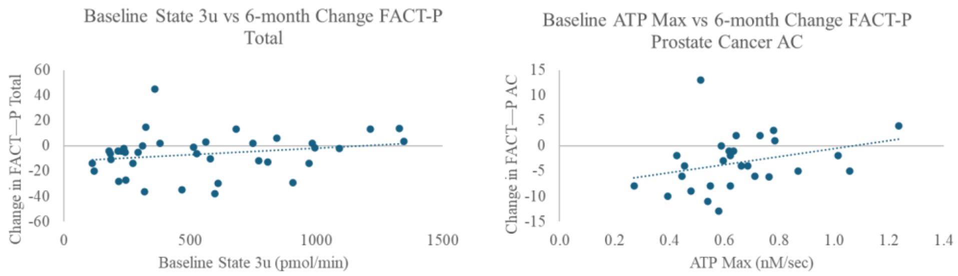

Supplemental Figure 5. Scatter plots depicting significant correlations between baseline values and 6-month changes in variables, as identified in Supplemental Figure 4.

Six-month Change

Six-month Change	Fat Mass	ALM	VO_2 Peak	6MWT	Physical activity	ATP Max[B]	HGS	SCP	FACT-P		EORTC QLQ C-30				
									PWB	Total	PF	RF	CF	SF	Appetite loss
BMI	.75**	.67**		.30*				.44**							-.35**
Fat Mass		.32*	-.38*												
ALM				.41**				.38**		.28*	.34*		.29*		-.60**
VO_2 Peak												.42**		.34*	
6MWT							.38**				.41**				-.36**
Physical activity															
State 3u[A]															
ATP Max[B]															
HGS															
SCP									.32*						-.31*

Spearman's rho correlation

-1 -.5 0 .5 1

Supplemental Figure 6. Six-month change associations between body composition, physical function, patient reported outcomes and mitochondrial function. Percentage change was used for correlations in body composition and physical function, while absolute values were used for patient-reported outcomes. Association between 6-month changes were assessed using two-sided Spearman correlation analysis. For pairs showing significant associations, partial Spearman correlations were subsequently performed, adjusting for the corresponding baseline variable—provided those variables were also significantly correlated at baseline. [A] *Ex vivo* respirometry [B] Magnetic resonance spectroscopy measurements. Abbreviations: BMI, body mass index; ALM,

appendicular lean mass; 6MWT, six-minute walk test; HGS, hand grip strength; SCP, stair climb power; FACT-P, Functional Assessment of Cancer Therapy-Prostate; PWB, physical well-being; EORTC QLQ-C30, European Organization for Research and Treatment of Cancer Quality of Life Questionnaire; PF, physical functioning; RF, role functioning; CF, cognitive functioning; SF, social functioning. * $p < 0.05$, ** $p < 0.01$.

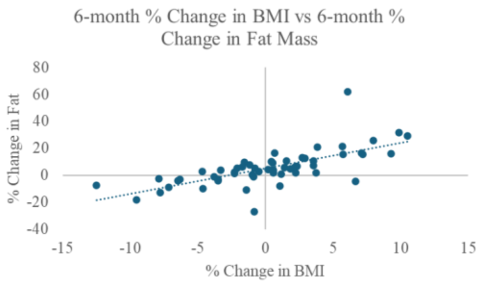
6-month % Change in BMI vs 6-month % Change in Fat Mass
% Change in Fat
% Change in BMI

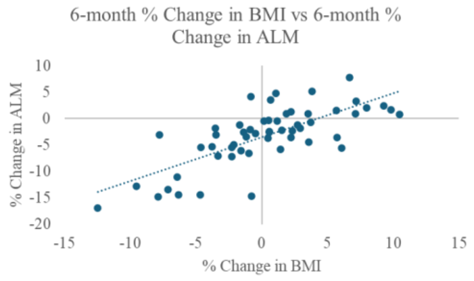
6-month % Change in BMI vs 6-month % Change in ALM
% Change in ALM
% Change in BMI

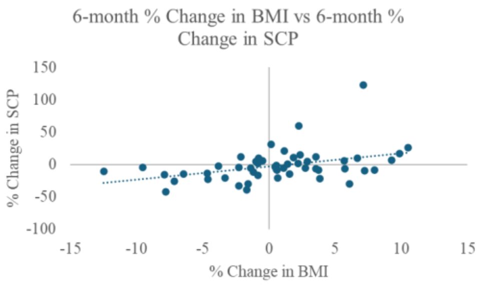
6-month % Change in BMI vs 6-month % Change in SCP
% Change in SCP
% Change in BMI

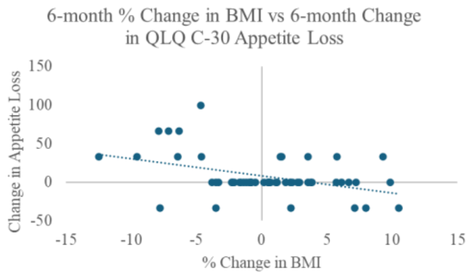
6-month % Change in BMI vs 6-month Change in QLQ C-30 Appetite Loss
Change in Appetite Loss
% Change in BMI

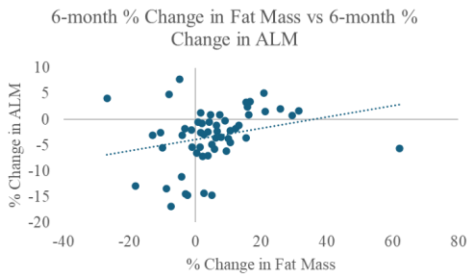
6-month % Change in Fat Mass vs 6-month % Change in ALM
% Change in ALM
% Change in Fat Mass

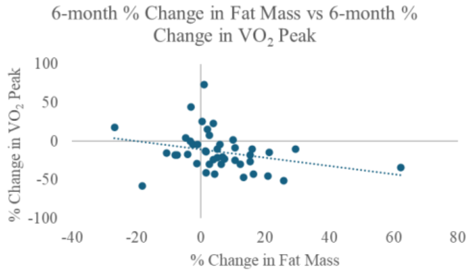
6-month % Change in Fat Mass vs 6-month % Change in VO_2 Peak
% Change in VO_2 Peak
% Change in Fat Mass

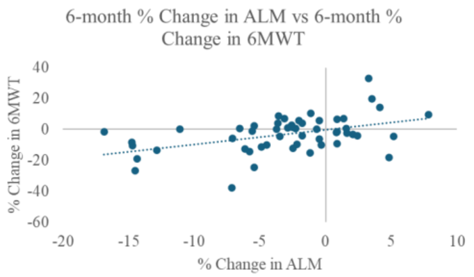
6-month % Change in ALM vs 6-month % Change in 6MWT
% Change in 6MWT
% Change in ALM

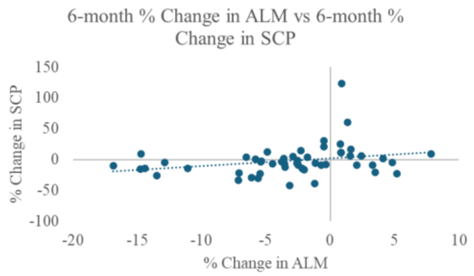
6-month % Change in ALM vs 6-month % Change in SCP
% Change in SCP
% Change in ALM

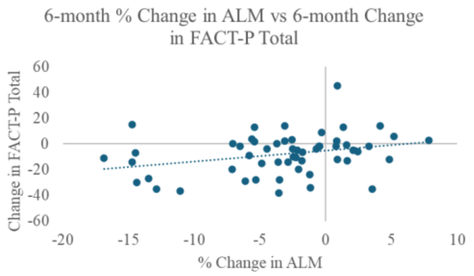
6-month % Change in ALM vs 6-month Change in FACT-P Total
Change in FACT-P Total
% Change in ALM

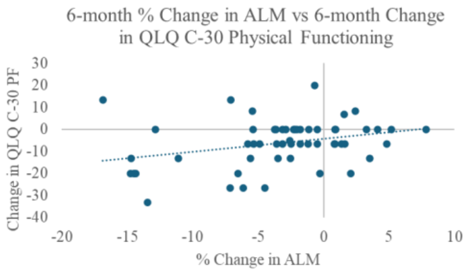
6-month % Change in ALM vs 6-month Change in QLQ C-30 Physical Functioning
Change in QLQ C-30 PF
% Change in ALM

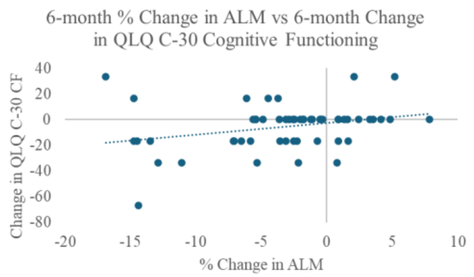
6-month % Change in ALM vs 6-month Change in QLQ C-30 Cognitive Functioning
Change in QLQ C-30 CF
% Change in ALM

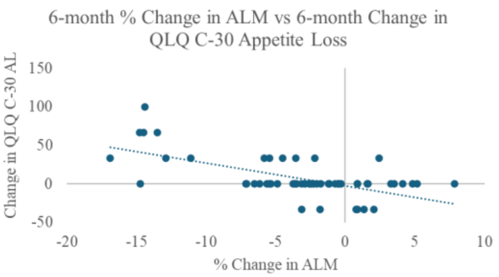
6-month % Change in ALM vs 6-month Change in QLQ C-30 Appetite Loss
Change in QLQ C-30 AL
% Change in ALM

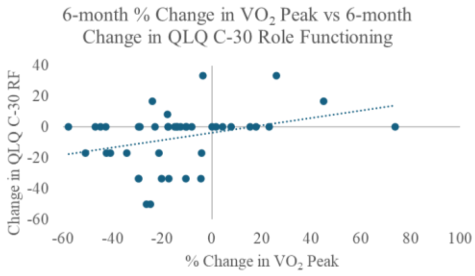
6-month % Change in VO_2 Peak vs 6-month Change in QLQ C-30 Role Functioning
Change in QLQ C-30 RF
% Change in VO_2 Peak

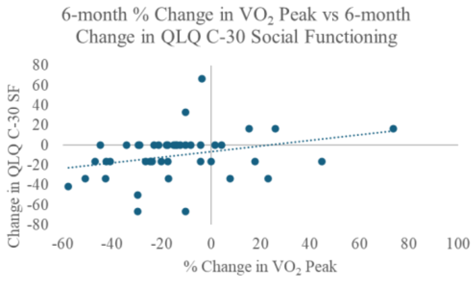
6-month % Change in VO_2 Peak vs 6-month Change in QLQ C-30 Social Functioning
Change in QLQ C-30 SF
% Change in VO_2 Peak

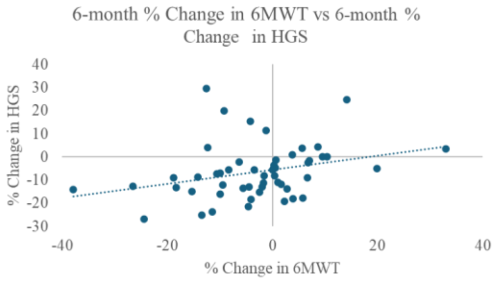
6-month % Change in 6MWT vs 6-month % Change in HGS
% Change in HGS
% Change in 6MWT

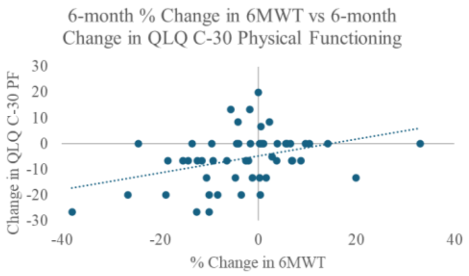
6-month % Change in 6MWT vs 6-month Change in QLQ C-30 Physical Functioning
Change in QLQ C-30 PF
% Change in 6MWT

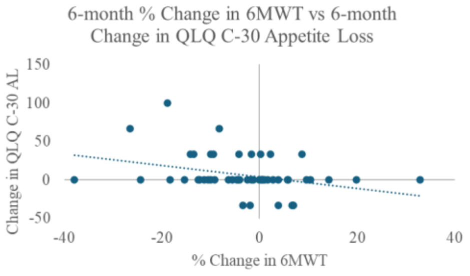
6-month % Change in 6MWT vs 6-month Change in QLQ C-30 Appetite Loss
Change in QLQ C-30 AL
% Change in 6MWT

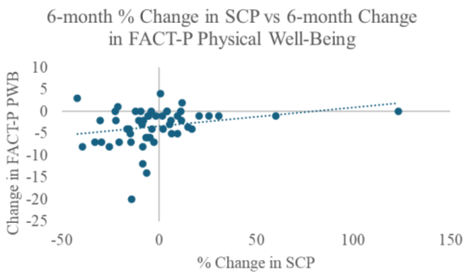
6-month % Change in SCP vs 6-month Change in FACT-P Physical Well-Being
Change in FACT-P PWB
% Change in SCP

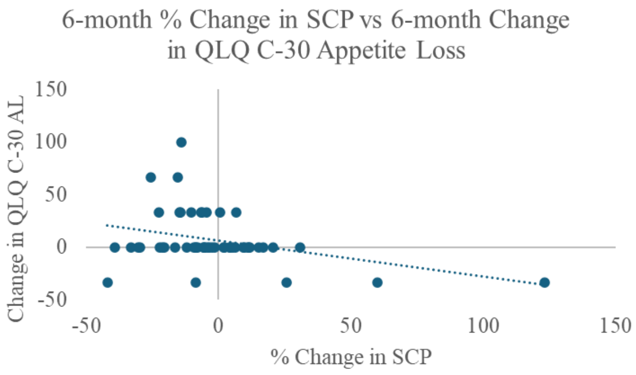

Supplemental Figure 7. Scatter plots showing significant correlations between 6-month changes in variables, as identified in Supplemental Figure 6

A

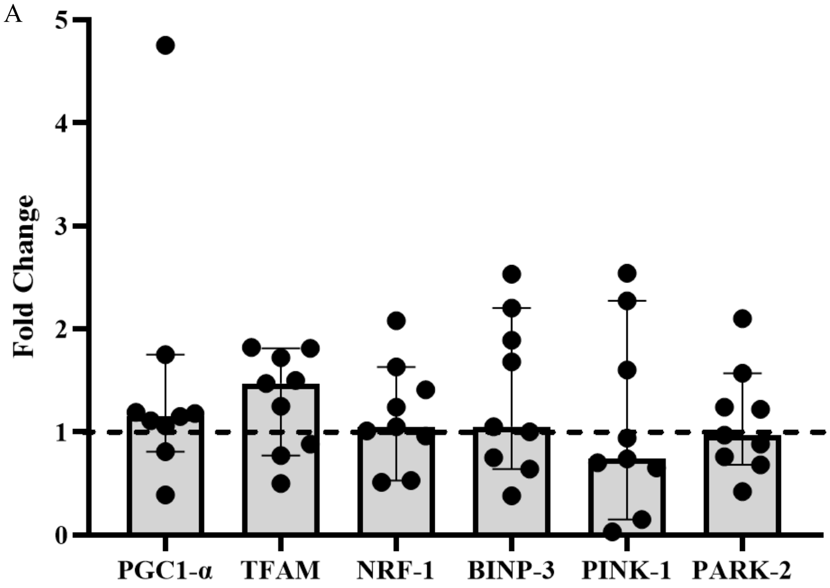

B

	Baseline							Six-month Change	
	State 3u[A]	Maximum ATP[B]	VO_2 Peak	6MWT	HGS	SCP	ALM	VO_2 Peak	6MWT
PGC1-α			-.75*	-.70*					-.72*
BNIP-3			-.75*						-.72*
NRF-1	-.67*								
PARK-2									
PINK-1			-.64*						-.80**
TFAM			-.71*						-.75*

(Row group label: Baseline)

Supplemental Figure 8. A) Fold change in skeletal muscle mRNA expression of genes involved in mitochondrial biogenesis (PGC1-α, TFAM, NRF-1) and mitophagy (BNIP-3, PINK-1, PARK-1) after six months of ADT. Gene expression is reported as ΔCT (target gene – GAPDH), where lower ΔCT values indicate greater expression. B) Heatmap illustrates correlations between baseline gene expression with baseline mitochondrial function (State 3u and Maximum ATP

max) and clinical outcomes of interest and 6-month percentage change in VO_2 peak and 6MWT (N = 9-10). *: P value < 0.05. Abbreviations: PGC1-α, peroxisome proliferator–activated receptor gamma coactivator 1-alpha; TFAM, mitochondrial transcription factor A; NRF-1, nuclear respiratory factor 1; BNIP-3, BCL2 interacting protein 3; PINK-1, PTEN-induced kinase 1; PARK-1, parkin; 6MWT, six-minute walk test; HGS, handgrip strength; SCP, stair climb power; ALM, appendicular lean mass.

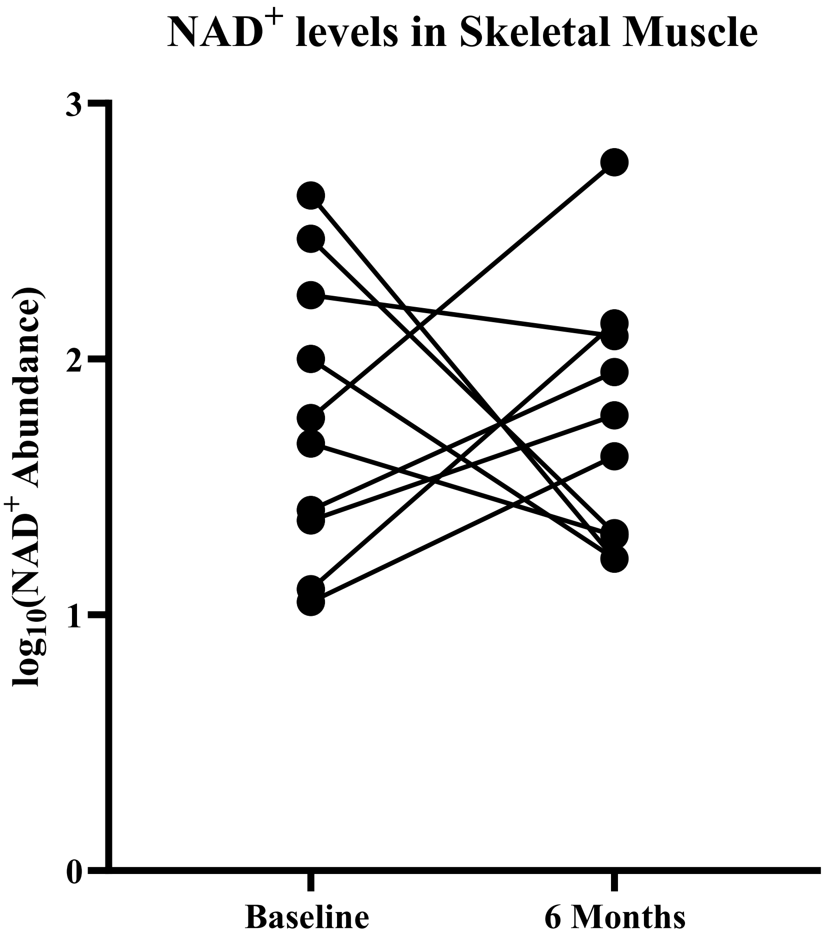

Supplemental Figure 9. Six-month change in nicotinamide adenine dinucleotide (NAD) levels assessed by mass spectrometry. Changes from baseline to six months were evaluated using a two-sided Wilcoxon signed-rank test. The exact P value was 0.91.

Median (IQR)	> Median VO_2 Peak (N=10-28)	< Median VO_2 Peak (10-28)	P-value[A]
Body Weight (kg)	81.1 (71.6, 90.6)	96.1 (78.5, 103)	**0.014**
BMI (kg/m²)	26.1 (24.4, 29.2)	29.1 (26.1, 31.3)	**0.042**
DEXA Fat (kg)	22.4 (16.7, 27.3)	28.8 (22.9, 35.3)	**0.004**
DEXA ALM (kg)	24.5 (22.3, 26.5)	25.8 (22.1, 29.2)	0.39
6MWT (m)	571.9 (515.1, 636.7)	480.4 (398.8, 534.3)	**0.021**
Non-sedentary Activity (minutes/day)	79.1 (46.3, 107)	49.9 (19.9, 99.5)	0.094
SCP (W)	403.1 (334.8, 459.3)	327.2 (226.2, 436)	**0.020**
HGS (kg)	44 (40, 47.8)	38.5 (31.4, 45)	**0.021**
State 3 (pmol/min)	413.5 (258, 665.4)	379.8 (233.4, 628.7)	0.70
State 3u (pmol/min)	490.8 (288.1, 816.3)	320.8 (216.2, 860.8)	0.44
ATP Max (nM/sec)	0.64 (0.58, 0.79)	0.54 (0.42, 0.65)	**0.027**
FACTP PWB	26 (25, 28)	24 (22.3, 26.8)	**0.013**
FACTP SFWB	23 (18, 24.4)	23.5 (17.3, 25.9)	0.63
FACTP EWB	20 (17, 21)	20 (17.3, 22)	0.63
FACTP FWB	23 (18.3, 26)	20.5 (14.3, 24.8)	0.19
FACTP Pca AC	35 (31, 39.8)	29.5 (25.3, 37.8)	0.076
FACTP Total	122 (111.3, 139.5)	113.3 (100, 131.3)	0.19
QLQ-C30 QOL	79.2 (66.7, 97.9)	66.7 (50, 83.3)	**0.026**
QLQ-C30 PF	100 (93.3, 100)	80 (68.3, 93.3)	**0.001**
QLQ-C30 RF	100 (100, 100)	87.5 (66.7, 100)	**0.022**
QLQ-C30 EF	83.3 (66.7, 100)	87.5 (66.7, 100)	0.97
QLQ-C30 CF	100 (83.3, 100)	100 (83.3, 100)	0.96
QLQ-C30 SF	100 (87.5, 100)	83.3 (66.7, 100)	**0.004**
QLQ-C30 Fatigue	11.1 (0, 33.3)	33.3 (11.1, 33.3)	**0.034**
QLQ-C30 Pain	16.7 (0, 33.3)	25 (16.7, 50)	0.077
QLQ-C30 Dyspnea	0 (0, 33.3)	33.3 (0, 33.3)	**0.021**
QLQ-C30 Insomnia	33.3 (0, 33.3)	33.3 (0, 66.7)	0.093
QLQ-C30 Appetite Loss	0 (0, 33.3)	0 (0, 33.3)	0.28

Supplemental Table 1: Comparisons of outcomes between participants with baseline VO_2 peak greater than the median (17.25 ml/kg/min) and those with VO_2 peak less than or equal to the median. [A]Group differences were assessed using a two-sided Mann-Whitney U test. Exact P values are reported where available. No adjustments were made for multiple comparisons. Abbreviations: IQR, Interquartile Range; BMI, body mass index; DEXA, Dual-Energy X-ray

Absorptiometry; ALM, appendicular lean mass; 6MWT, six minute walk test; SCP, stair climb power; W, watts; HGS, hand grip strength; FACT P, Functional assessment of cancer therapy prostate; PWB, physical well-being; SFWB, social family well-being; EWB, emotional well-being; FWB, functional well-being; Pca AC, prostate cancer additional concerns; QLQ C-30, European Organization for Research and Treatment of Cancer Quality of Life Questionnaire Core 30; QOL, quality of life; PF, physical functioning; RF, role functioning; EF, emotional functioning; CF, cognitive functioning; SF, social functioning.

Median (IQR)	> Median ATP Max (N=14-15)	< Median ATP Max (N=9-15)	P-value[A]
Body Weight (kg)	78.2 (69.5, 85.9)	97.7 (83.6, 102.3)	**0.008**
BMI (kg/m^2)	26.2 (24, 27.8)	29.7 (26, 31.3)	**0.026**
DEXA Fat (kg)	20.1 (15.7, 27)	30.5 (23.7, 34.7)	**0.009**
DEXA ALM (kg)	23.2 (21.3, 26.4)	27 (24.3, 30.1)	**0.016**
VO_2 Peak (mL/kg/min)	24.5 (18.2, 28.3)	17.9 (13.8, 20.7)	**0.026**
6MWT (m)	578.2 (557.8, 627.9)	488 (452.6, 534.6)	**0.004**
Non-sedentary Activity (minutes/day)	88.1 (62.3, 155.3)	71.3 (36.7, 99.7)	0.085
SCP (W)	418.3 (353.6, 475.5)	420.7 (304.1, 455.1)	0.72
HGS (kg)	43 (34, 46)	46.5 (44, 51)	**0.026**
State 3 (pmol/min)	489.2 (251.8, 644.9)	321.8 (193.4, 936.9)	0.60
State 3u (pmol/min)	572.8 (304.5, 789.8)	320.6 (226.6, 989)	0.64
FACTP PWB	28 (25, 28)	26 (23, 27)	0.081
FACTP SFWB	23 (18, 25)	21 (15, 26)	0.97
FACTP EWB	19 (14, 21)	20 (17, 23)	0.41
FACTP FWB	23 (15, 27)	24 (16, 26)	0.84
FACTP Pca AC	36 (32, 42)	34 (29, 38)	0.35
FACTP Total	125 (105.2, 143)	123 (98, 138)	0.68
QLQ-C30 QOL	83.3 (66.7, 100)	75 (50, 83.3)	0.074
QLQ-C30 PF	100 (93.3, 100)	91.7 (66.7, 100)	0.089
QLQ-C30 RF	100 (100, 100)	100 (66.7, 100)	0.061
QLQ-C30 EF	83.3 (66.7, 100)	91.7 (75, 100)	0.62
QLQ-C30 CF	100 (66.7, 100)	100 (79.2, 100)	0.56
QLQ-C30 SF	100 (100, 100)	100 (66.7, 100)	0.20
QLQ-C30 Fatigue	11 (0, 22.3)	22.3 (0, 33.3)	0.13
QLQ-C30 Pain	16.7 (0, 33.3)	16.7 (0, 33.3)	0.41
QLQ-C30 Dyspnea	0 (0, 33.3)	0 (0, 33.3)	0.62
QLQ-C30 Insomnia	0 (0, 33.3)	33.3 (0, 66.7)	0.23
QLQ-C30 Appetite Loss	0 (0, 0)	0 (0, 33.3)	0.37

Supplemental Table 2: Comparisons of outcomes between participants with baseline ATP Maximum greater than the median (0.62 nM/sec) and those with ATM Maximum less than or equal to the median. [A] Group differences were assessed using a two-side Mann-Whitney U-test. Exact P values are reported where available. No adjustments were made for multiple comparisons. Abbreviations: IQR, Interquartile Range; BMI, body mass index; DEXA, Dual-Energy X-ray Absorptiometry; ALM, appendicular lean mass; 6MWT, six minute walk test; SCP, stair climb power; W, watts; HGS, hand grip strength; FACT P, Functional assessment of cancer

therapy prostate; PWB, physical well-being; SFWB, social family well-being; EWB, emotional well-being; FWB, functional well-being; Pca AC, prostate cancer additional concerns; QLQ C-30, European Organization for Research and Treatment of Cancer Quality of Life Questionnaire Core 30; QOL, quality of life; PF, physical functioning; RF, role functioning; EF, emotional functioning; CF, cognitive functioning; SF, social functioning.

Median (IQR)	> Median State 3u (N=13-20)	< Median State 3u (N=10-21)	P-value[A]
Body Weight (kg)	83.2 (78.4, 97.1)	90.6 (79.8, 101.1)	0.37
BMI (kg/m^2)	26.2 (25.2, 29.9)	29 (25.7, 30)	0.24
DEXA Fat (kg)	22.6 (19.8, 33.5)	29.4 (22.6, 32.3)	0.26
DEXA ALM (kg)	24.9 (22.8, 27.2)	25.4 (22.9, 27.9)	0.84
VO_2 Peak (mL/kg/min)	21.7 (15.3, 30.1)	17.5 (14.1, 24.4)	0.15
6MWT (m)	569.1 (530.7, 627.5)	512.4 (455.7, 564.6)	0.090
Non-sedentary Activity (minutes/day)	76 (44.3, 146.1)	79.1 (37.6, 113.2)	0.58
SCP (W)	409.7 (308.9, 441.9)	374.9 (278.1, 452.8)	0.62
HGS (kg)	43.3 (37.4, 46.9)	43.5 (34, 46.3)	0.82
ATP Max (nM/sec)	0.73 (0.63, 0.94)	0.55 (0.42, 0.71)	**0.021**
FACTP PWB	27 (24.5, 28)	24 (23, 27)	**0.044**
FACTP SFWB	24 (18, 25.5)	21 (18, 25)	0.69
FACTP EWB	21 (14, 22.8)	18 (16.5, 20.5)	0.28
FACTP FWB	23 (18.3, 27)	19 (14.5, 24.5)	0.14
FACTP Pca AC	35.5 (28.8, 41.8)	33 (25.5, 36.5)	0.16
FACTP Total	128 (108, 144.5)	113 (99.5, 134)	0.14
QLQ-C30 QOL	83.3 (68.8, 97.9)	66.7 (58.3, 91.7)	0.14
QLQ-C30 PF	100 (92.1, 100)	86.7 (66.7, 100)	**0.018**
QLQ-C30 RF	100 (100, 100)	100 (66.7, 100)	**0.030**
QLQ-C30 EF	83.3 (68.8, 100)	91.7 (66.7, 100)	0.85
QLQ-C30 CF	100 (83.3, 100)	83.3 (75, 100)	0.25
QLQ-C30 SF	100 (83.3, 100)	100 (66.7, 100)	0.25
QLQ-C30 Fatigue	11 (0, 22.3)	22.3 (5.5, 33.3)	**0.047**
QLQ-C30 Pain	16.7 (0, 33.3)	16.7 (0, 41.7)	0.40
QLQ-C30 Dyspnea	0 (0, 33.3)	33.3 (0, 33.3)	0.094
QLQ-C30 Insomnia	0 (0, 33.3)	33.3 (33.3, 66.7)	**0.015**
QLQ-C30 Appetite Loss	0 (0, 0)	0 (0, 33.3)	0.55

Supplemental Table 3: Comparisons of outcomes between participants with baseline State 3u greater than the median (468 pmol/min) and those with State 3u less than or equal to the median. [A] Group differences were assessed using a two-side Mann-Whitney U-test. Exact P values are reported where available. No adjustments were made for multiple comparisons. Abbreviations: IQR, Interquartile Range; BMI, body mass index; DEXA, Dual-Energy X-ray Absorptiometry;

ALM, appendicular lean mass; 6MWT, six minute walk test; SCP, stair climb power; W, watts; HGS, hand grip strength; FACT P, Functional assessment of cancer therapy prostate; PWB, physical well-being; SFWB, social family well-being; EWB, emotional well-being; FWB, functional well-being; Pca AC, prostate cancer additional concerns; QLQ C-30, European Organization for Research and Treatment of Cancer Quality of Life Questionnaire Core 30; QOL, quality of life; PF, physical functioning; RF, role functioning; EF, emotional functioning; CF, cognitive functioning; SF, social functioning.

Variable	Beta	SE	95 % CI		Wald Chi-Square	P-value
			Lower	Upper		
**Body Mass Index** (N=55-57)						
T3-T1	0.00	0.19	-0.37	0.37	0.00	0.99
T2-T1	0.06	0.12	-0.17	0.29	0.24	0.63
Stage 4 – Stage 2	-2.01	1.24	-4.43	0.42	2.63	0.11
Stage 3 – Stage 2	-0.38	1.21	-2.76	2.00	0.10	0.76
Age	-0.06	0.06	-0.18	0.06	1.04	0.31
**Fat Mass** (N=55-57)						
T3-T1	1.12	0.37	0.39	1.85	9.10	**0.003**
T2-T1	0.71	0.27	0.19	1.23	7.18	**0.007**
Stage 4 – Stage 2	-5.57	2.66	-10.78	-0.36	4.39	0.04
Stage 3 – Stage 2	-0.22	2.61	-5.33	4.89	0.01	0.93
Age	-0.11	0.14	-0.38	0.15	0.68	0.41
**Appendicular Lean Mass** (N=55-57)						
T3-T1	-0.88	0.19	-1.26	-0.51	21.59	**<0.001**
T2-T1	-0.43	0.17	-0.76	-0.09	6.10	**0.014**
Stage 4 – Stage 2	-0.95	1.45	-3.79	1.89	0.43	0.51
Stage 3 – Stage 2	-0.85	1.23	-3.26	1.56	0.48	0.49
Age	-0.12	0.07	-0.25	0.01	3.30	0.07
**Hand Grip Strength** (N=55-57)						
T3-T1	-2.9	0.59	-4.1	-1.8	24.6	**<0.0001**
T2-T1	-2.26	0.55	-3.3	-1.2	17.1	**<0.0001**
Stage 4 – Stage 2	-2.2	2.5	-7.1	2.8	0.74	0.39
Stage 3 – Stage 2	-1.8	2.2	-6.1	2.5	0.68	0.41
Age	-0.41	0.14	-0.69	-0.13	8.13	**0.004**
**VO_2 Peak**[A] (N=43-55)						
T3-T1	-0.08	0.02	-0.12	-0.04	19.71	**<0.001**
T2-T1	-0.06	0.01	-0.08	-0.04	23.57	**<0.001**
Stage 4 – Stage 2	0.04	0.06	-0.08	0.15	0.40	0.53
Stage 3 – Stage 2	-0.03	0.05	-0.14	0.08	0.26	0.61
Age	-0.01	0.00	-0.01	0.00	2.40	0.12
**6-Minute Walk Test**[A] (N=52-57)						
T3-T1	-0.019	0.007	-0.033	-0.004	6.42	**0.011**
T2-T1	-0.005	0.007	-0.019	0.010	0.41	0.52
Stage 4 – Stage 2	0.018	0.037	-0.056	0.091	0.22	0.64
Stage 3 – Stage 2	0.015	0.030	-0.044	0.074	0.25	0.61
Age	-0.003	0.002	-0.007	0.001	2.15	0.14
**Stair Climb Power** (N=54-55)						
T3-T1	-13.5	11.2	-35.5	8.4	1.5	0.23
T2-T1	-13.1	8.2	-29.3	2.9	2.6	0.11
Stage 4 – Stage 2	-7.7	37.2	-80.7	65.2	0.043	0.84
Stage 3 – Stage 2	7.8	33.8	-58.4	73.9	0.54	0.82
Age	-5.5	2.0	-9.4	-1.5	7.5	**0.006**
**Non-sedentary Physical Activity**[A] (N=50-54)						
T3-T1	-0.098	0.05	-0.196	0.001	3.8	0.051
T2-T1	-0.020	0.05	-0.109	0.069	0.196	0.66
Stage 4 – Stage 2	-0.070	0.19	-0.44	0.29	0.139	0.71
Stage 3 – Stage 2	0.014	0.15	-0.28	0.31	0.009	0.92
Age	-0.023	0.01	-0.042	-0.004	5.86	**0.016**

Supplemental Table 4: Longitudinal analysis of body composition and physical function after six months of androgen deprivation therapy using generalized estimating equations. Normality was assessed using the Kolmogorov-Smirnov test. [A]Variables that were not normally distributed were log-transformed prior to analysis. Statistical tests were two sided. Model estimates are presented as β coefficients with standard errors (SE), 95% confidence intervals (CI), and exact P values where available. No adjustments were made for multiple comparisons. Abbreviations: T3-TI; Difference between timepoint 3 (6 months) and timepoint 1 (baseline); T2-T1, Difference between timepoint 2 (3 months) and timepoint 1 (baseline).

Variable[A]	Beta	SE	95 % CI		Wald Chi-Square	P-value
			Lower	Upper		
**Physical Well-Being**						
T3-T1	-0.08	0.02	-0.12	-0.05	23.81	**<0.001**
T2-T1	-0.07	0.02	-0.10	-0.03	12.89	**<0.001**
Stage 4 – Stage 2	0.02	0.04	-0.06	0.11	0.33	0.57
Stage 3 – Stage 2	0.02	0.05	-0.08	0.12	0.12	0.73
Age	0.01	0.00	0.00	0.01	8.21	**0.004**
**Social Family Well-Being**						
T3-T1	-0.04	0.03	-0.09	0.02	1.54	0.22
T2-T1	-0.01	0.02	-0.04	0.03	0.13	0.72
Stage 4 – Stage 2	0.03	0.04	-0.05	0.11	0.47	0.49
Stage 3 – Stage 2	-0.03	0.04	-0.11	0.06	0.38	0.54
Age	0.00	0.00	0.00	0.01	3.10	0.078
**Emotional Well-Being**						
T3-T1	0.01	0.01	-0.01	0.03	1.14	0.29
T2-T1	0.01	0.01	-0.01	0.04	1.09	0.30
Stage 4 – Stage 2	0.01	0.02	-0.03	0.04	0.18	0.67
Stage 3 – Stage 2	-0.02	0.03	-0.07	0.03	0.53	0.47
Age	0.004	0.0013	0.001	0.006	9.55	**0.002**
**Functional Well-Being**						
T3-T1	-0.04	0.02	-0.09	0.01	2.79	0.095
T2-T1	-0.04	0.02	-0.08	-0.01	5.44	**0.02**
Stage 4 – Stage 2	0.08	0.06	-0.04	0.20	1.76	0.19
Stage 3 – Stage 2	0.03	0.07	-0.11	0.17	0.18	0.67
Age	0.01	0.00	0.00	0.02	9.05	**0.003**
**Prostate Cancer Additional**						
T3-T1	-0.06	0.02	-0.09	-0.02	11.53	**<0.001**
T2-T1	-0.04	0.01	-0.06	-0.01	9.41	**0.002**
Stage 4 – Stage 2	0.03	0.04	-0.05	0.11	0.62	0.43
Stage 3 – Stage 2	0.02	0.04	-0.06	0.10	0.21	0.64
Age	0.01	0.00	0.00	0.01	5.78	**0.016**
**Total**						
T3-T1	-0.04	0.01	-0.06	-0.02	11.84	**<0.001**
T2-T1	-0.03	0.01	-0.05	-0.01	11.15	**<0.001**
Stage 4 – Stage 2	0.03	0.03	-0.02	0.08	1.15	0.28
Stage 3 – Stage 2	0.00	0.03	-0.07	0.07	0.00	0.99
Age	0.01	0.00	0.00	0.01	11.20	**<0.001**

Supplemental Table 5: Longitudinal analysis of FACT-P (Functional Assessment of Cancer Therapy – Prostate) after six months of androgen deprivation therapy using generalized estimating equations. Normality was assessed using the Kolmogorov-Smirnov test. [A]Variables that were not normally distributed were log-transformed prior to analysis. Statistical tests were two sided. Model estimates are presented as β coefficients with standard errors (SE), 95% confidence intervals (CI), and exact P values where available. No adjustments were made for

multiple comparisons. Abbreviations: T3-TI; Difference between timepoint 3 (6 months) and timepoint 1 (baseline); T2-T1, Difference between timepoint 2 (3 months) and timepoint 1 (baseline). N = 55-57.

Variable[A]	Beta	SE	95 % CI		Wald Chi-Square	P-value
			Lower	Upper		
**Quality of Life**						
T3-T1	-0.04	0.03	-0.10	0.02	1.85	0.17
T2-T1	-0.01	0.02	-0.06	0.04	0.27	0.61
Stage 4 – Stage 2	0.02	0.04	-0.06	0.10	0.28	0.60
Stage 3 – Stage 2	-0.01	0.05	-0.10	0.08	0.06	0.81
Age	0.01	0.00	0.00	0.01	8.18	**0.004**
**Physical Func.**						
T3-T1	-0.03	0.01	-0.05	-0.01	10.56	**0.001**
T2-T1	-0.02	0.01	-0.03	0.00	2.34	0.13
Stage 4 – Stage 2	0.01	0.04	-0.07	0.08	0.03	0.85
Stage 3 – Stage 2	0.03	0.03	-0.03	0.09	1.29	0.26
Age	0.00	0.00	0.00	0.01	0.57	0.45
**Role Func.**						
T3-T1	-0.05	0.04	-0.13	0.03	1.73	0.19
T2-T1	-0.05	0.05	-0.14	0.04	1.05	0.31
Stage 4 – Stage 2	-0.02	0.10	-0.21	0.17	0.06	0.81
Stage 3 – Stage 2	0.07	0.07	-0.06	0.20	1.00	0.32
Age	0.01	0.00	0.00	0.02	3.41	0.07
**Emotional Func.**						
T3-T1	-0.01	0.01	-0.04	0.02	0.70	0.40
T2-T1	-0.01	0.02	-0.05	0.03	0.35	0.55
Stage 4 – Stage 2	0.02	0.04	-0.06	0.11	0.28	0.60
Stage 3 – Stage 2	0.00	0.05	-0.10	0.11	0.00	0.96
Age	0.01	0.00	0.00	0.01	12.22	**<0.001**
**Cognitive Func.**						
T3-T1	-0.03	0.02	-0.06	0.01	2.37	0.12
T2-T1	-0.02	0.02	-0.05	0.02	1.17	0.28
Stage 4 – Stage 2	0.05	0.05	-0.05	0.15	0.95	0.33
Stage 3 – Stage 2	0.06	0.05	-0.03	0.15	1.61	0.20
Age	0.01	0.00	0.00	0.01	8.13	**0.004**
**Social Func.**						
T3-T1	-0.12	0.04	-0.19	-0.04	8.89	**0.003**
T2-T1	-0.06	0.04	-0.15	0.02	2.04	0.15
Stage 4 – Stage 2	0.08	0.10	-0.12	0.27	0.62	0.43
Stage 3 – Stage 2	0.02	0.12	-0.22	0.25	0.03	0.87
Age	0.01	0.01	0.00	0.03	4.46	**0.035**
**Fatigue**						
T3-T1	0.23	0.08	0.07	0.39	7.81	**0.005**
T2-T1	0.28	0.08	0.13	0.43	13.01	**<0.001**
Stage 4 – Stage 2	-0.07	0.19	-0.43	0.30	0.12	0.73
Stage 3 – Stage 2	-0.01	0.16	-0.32	0.31	0.00	0.96
Age	-0.01	0.01	-0.03	0.00	2.41	0.12

Variable[A]	Beta	SE	95 % CI		Wald Chi-Square	P-value
			Lower	Upper		
**Nausea**						
T3-T1	0.27	0.09	0.10	0.44	9.53	**0.002**
T2-T1	0.07	0.07	-0.07	0.21	0.90	0.34
Stage 4 – Stage 2	0.06	0.15	-0.24	0.36	0.14	0.71
Stage 3 – Stage 2	-0.06	0.15	-0.36	0.24	0.15	0.70
Age	-0.02	0.01	-0.04	-0.01	6.66	**0.01**
**Pain**						
T3-T1	-0.03	0.09	-0.21	0.15	0.12	0.73
T2-T1	-0.13	0.09	-0.31	0.06	1.81	0.18
Stage 4 – Stage 2	0.15	0.23	-0.29	0.60	0.45	0.50
Stage 3 – Stage 2	-0.02	0.20	-0.41	0.37	0.01	0.92
Age	-0.02	0.01	-0.04	0.00	3.05	0.08
**Dyspnea**						
T3-T1	0.11	0.11	-0.11	0.34	0.98	0.32
T2-T1	0.10	0.11	-0.11	0.32	0.88	0.35
Stage 4 – Stage 2	-0.07	0.23	-0.52	0.38	0.09	0.76
Stage 3 – Stage 2	0.19	0.22	-0.23	0.62	0.80	0.37
Age	-0.01	0.01	-0.04	0.01	0.73	0.39
**Insomnia**						
T3-T1	0.23	0.10	0.04	0.43	5.56	**0.018**
T2-T1	0.15	0.12	-0.09	0.38	1.50	0.22
Stage 4 – Stage 2	0.27	0.20	-0.13	0.67	1.70	0.19
Stage 3 – Stage 2	0.18	0.19	-0.18	0.55	0.95	0.33
Age	-0.05	0.01	-0.06	-0.03	23.30	**<0.001**
**Appetite Loss**						
T3-T1	0.11	0.10	-0.09	0.31	1.17	0.28
T2-T1	0.06	0.10	-0.14	0.25	0.34	0.56
Stage 4 – Stage 2	0.19	0.23	-0.27	0.64	0.64	0.42
Stage 3 – Stage 2	-0.15	0.21	-0.55	0.26	0.50	0.48
Age	-0.03	0.01	-0.05	-0.01	6.28	**0.012**
**Constipation**						
T3-T1	0.30	0.11	0.08	0.52	7.39	**0.007**
T2-T1	0.13	0.09	-0.05	0.31	2.01	0.16
Stage 4 – Stage 2	0.05	0.21	-0.36	0.45	0.05	0.82
Stage 3 – Stage 2	-0.12	0.19	-0.48	0.25	0.39	0.53
Age	-0.02	0.01	-0.04	0.00	5.13	**0.024**
**Diarrhea**						
T3-T1	0.19	0.10	-0.02	0.39	3.31	0.069
T2-T1	0.21	0.09	0.03	0.39	5.42	**0.02**
Stage 4 – Stage 2	-0.20	0.19	-0.57	0.17	1.10	0.29
Stage 3 – Stage 2	0.03	0.20	-0.36	0.42	0.03	0.86
Age	-0.01	0.01	-0.03	0.01	1.70	0.19

Supplemental Table 6: Longitudinal analysis of EORTC QLQ C-30 (European Organization for Research and Treatment of Cancer Quality of Life Questionnaire) after six months of androgen deprivation therapy using generalized estimating equations. Normality was assessed using the Kolmogorov-Smirnov test. [A]Variables that were not normally distributed were log-transformed

prior to analysis. Statistical tests were two sided. Model estimates are presented as β coefficients with standard errors (SE), 95% confidence intervals (CI), and exact P values where available. No adjustments were made for multiple comparisons. Abbreviations: T3-TI; Difference between timepoint 3 (6 months) and timepoint 1 (baseline); T2-T1, Difference between timepoint 2 (3 months) and timepoint 1 (baseline). N = 55-58.

N = 21-24	6-Month Change	
	Median Change (IQR)	P value
**Ex-vivo Mitochondrial Respiration**		
State 2 (pmol/min)	9.1 (-43, 67)	0.48
State 3 (pmol/min)	48.8 (-100, 390)	0.26
State 4 (pmol/min)	12.2 (-32, 50)	0.45
State 3u (pmol/min)	214 (-168, 545)	0.13
**In-vivo Mitochondrial Respiration**		
Maximum ATP Synthesis	0.001 (-0.5, 0.13)	0.90

Supplemental Table 7: Changed in ex vivo and in vivo mitochondrial respiration were assessed using the two-sided Wilcoxon Signed Ranks Test of ex-vivo. Abbreviations: ATP, Adenosine Triphosphate; IQR, Inter-quartile range.

Dependent Variable	N	R^2	Significant Predictors (6- Month Change Assessments)	Unstd. B (95%)	P-value
6-Month % Change in 6MWT	42	0.22	6-Month % Change in ALM	0.86 (0.34, 1.4)	0.002
6-Month % Change in HGS	43	0.10	6-Month % Change in ALM	0.54 (0.03, 1.1)	0.038
6-Month Change in FACT-P Total	43	0.11	6-Month % Change in ALM	0.86 (0.09, 1.6)	0.031
6-Month Change in QLQ C-30 PF	43	0.092	6-Month % Change in ALM	0.50 (0.01, 0.99)	0.048
6-Month Change in QLQ C-30 RF	43	0.21	6-Month % Change in VO_2 Peak	0.25 (0.04, 0.45)	0.020
			Age	0.83 (0.08, 1.6)	0.030
6-Month Change in QLQ C-30 CF	43	0.10	6-Month % Change in ALM	1.0 (0.04, 2.0)	0.042
6-Month Change in QLQ C-30 SF	43	0.21	6-Month % Change in ALM	1.4 (0.29, 2.6)	0.016
6-Month Change in QLQ C-30 Appetite Loss	43	0.40	6-Month % Change in ALM	-2.9 (-4.0, -1.8)	<0.001

Supplemental Table 8: Stepwise multivariate regression analysis examining predictors of 6-month percent change in physical function and absolute change in patient-reported outcomes as the dependent variable and with age, metastasis, and 6-month percent change in ALM, fat mass and VO_2 peak as predictors. VO_2 peak was not included as a predictor in the model where VO_2 peak was the dependent variable. All statistical tests were two-sided. Model results are presented unstandardized β coefficients with 95 % confidence intervals and exact P values where available. No adjustments were made for multiple comparisons. Abbreviations: Unstd, unstandardized; ALM, appendicular lean mass; 6MWT, six minute walk test; HGS, hand grip strength; FACT P,

Functional assessment of cancer therapy prostate; QLQ C-30, European Organization for Research and Treatment of Cancer Quality of Life Questionnaire Core 30; PF, physical functioning; RF, role functioning; CF, cognitive functioning; SF, social functioning.
