# Supplementary material for: Muscle mitochondria, function, mass, and quality of life in prostate cancer during androgen deprivation therapy
Source: Nat Commun. 2026 May 27;17:6884. doi: 10.1038/s41467-026-73542-x (PMC13389078; doi:10.1038/s41467-026-73542-x)
Supplement: Supplementary file 2 — Description of Additional Supplementary Files [file 41467_2026_73542_MOESM2_ESM.pdf]

Description of Additional Supplementary Files

File Name: Supplementary Data 1

Description: Excel file containing the results of the GSEA analysis.
