# Supplementary material for: Muscle mitochondria, function, mass, and quality of life in prostate cancer during androgen deprivation therapy
Source: Nat Commun. 2026 May 27;17:6884. doi: 10.1038/s41467-026-73542-x (PMC13389078; doi:10.1038/s41467-026-73542-x)
Supplement: Supplementary file 4 — Reporting Summary [file 41467_2026_73542_MOESM4_ESM.pdf]

nature portfolio

Corresponding author(s): Jose M. Garcia

Last updated by author(s): Dec 15, 2025

Reporting Summary

Nature Portfolio wishes to improve the reproducibility of the work that we publish. This form provides structure for consistency and transparency in reporting. For further information on Nature Portfolio policies, see our Editorial Policies and the Editorial Policy Checklist.

Statistics

For all statistical analyses, confirm that the following items are present in the figure legend, table legend, main text, or Methods section.

n/a	Confirmed	
☐	☒	The exact sample size (*n*) for each experimental group/condition, given as a discrete number and unit of measurement
☒	☐	A statement on whether measurements were taken from distinct samples or whether the same sample was measured repeatedly
☐	☒	The statistical test(s) used AND whether they are one- or two-sided<br>*Only common tests should be described solely by name; describe more complex techniques in the Methods section.*
☐	☒	A description of all covariates tested
☐	☒	A description of any assumptions or corrections, such as tests of normality and adjustment for multiple comparisons
☐	☒	A full description of the statistical parameters including central tendency (e.g. means) or other basic estimates (e.g. regression coefficient) AND variation (e.g. standard deviation) or associated estimates of uncertainty (e.g. confidence intervals)
☐	☒	For null hypothesis testing, the test statistic (e.g. *F*, *t*, *r*) with confidence intervals, effect sizes, degrees of freedom and *P* value noted<br>*Give P values as exact values whenever suitable.*
☒	☐	For Bayesian analysis, information on the choice of priors and Markov chain Monte Carlo settings
☒	☐	For hierarchical and complex designs, identification of the appropriate level for tests and full reporting of outcomes
☐	☒	Estimates of effect sizes (e.g. Cohen's *d*, Pearson's *r*), indicating how they were calculated

Our web collection on statistics for biologists contains articles on many of the points above.

Software and code

Policy information about availability of computer code

Data collection	Data were collected using the following tools and equipment: Dual-energy X-ray absorptiometry (DEXA) for body composition; Jamar Hydraulic Dynamometer (J.A. Preston Corp., Clifton, NJ) and cycle ergometer for muscle function; Actical activity monitor (Philips Respironics, Murrysville, PA) for accelerometry; FACT-P and EORTC QLQ-C30 questionnaires for patient-reported outcomes; Agilent Seahorse XFe24 Analyzer and XF24 Cell Culture Microplates (Agilent Technologies) with Pierce Rapid Gold BCA Protein Assay (Thermo Fisher Scientific) for ex vivo mitochondrial respiration; 4.7T Bruker magnet with surface coil for in vivo mitochondrial respiration; Qiagen RNeasy Fibrous Tissue Mini Kit, BioTek Cytation 5, QuantiTect Reverse Transcription Kit, ABI 7500 Real-Time PCR System (Applied Biosystems), and TaqMan Gene Expression Assays (Thermo Fisher Scientific) for gene expression analysis; PIXUL sonicator (Matchstick Technologies), BCA Protein Assay (Thermo Fisher Scientific), and Trypsin/Lys-C digestion (Promega) for proteomics sample preparation; neoVanquish UHPLC (Thermo Scientific) and Orbitrap Exploris 480 mass spectrometer (Thermo Fisher Scientific) with Data Independent Acquisition (DIA) LC-MS/MS for proteomics measurements.
Data analysis	Proteomics data were processed using Skyline, FragPipe v21.1 with MS-Fragger, DIA-NN v1.8.2 beta 27, and the Human UniProt Reference Database (downloaded December 12, 2022). Differential protein abundance was analyzed with DESeq2 in R, and pathway enrichment was performed with Gene Set Enrichment Analysis (GSEA) using MSigDB Hallmark and Canonical Pathways. Network visualization used Cytoscape v3.8.0, and functional mitochondrial protein mapping used Ingenuity Pathway Analysis (IPA, Qiagen). Statistical analyses were performed in SPSS Statistics v29.0.1.0 and R.

For manuscripts utilizing custom algorithms or software that are central to the research but not yet described in published literature, software must be made available to editors and reviewers. We strongly encourage code deposition in a community repository (e.g. GitHub). See the Nature Portfolio guidelines for submitting code & software for further information.

Data

Policy information about availability of data

All manuscripts must include a data availability statement. This statement should provide the following information, where applicable:

- Accession codes, unique identifiers, or web links for publicly available datasets
- A description of any restrictions on data availability
- For clinical datasets or third party data, please ensure that the statement adheres to our policy

The datasets generated and analyzed in this study on prostate cancer patients undergoing androgen deprivation therapy (ADT) are available as follows. Publicly Available Data: The raw proteomics data generated in this study will be deposited in the PRIDE,and will be available at [PXD061070]. Source data file will be provided with the raw data used for analysis.

Research involving human participants, their data, or biological material

Policy information about studies with human participants or human data. See also policy information about sex, gender (identity/presentation), and sexual orientation and race, ethnicity and racism.

Reporting on sex and gender	This study focused on biologically male patients with prostate cancer undergoing androgen deprivation therapy. Gender identity was not assessed, as the study focused on biological sex due to the inclusion criteria requiring a diagnosis of prostate cancer, a condition that occurs in individuals assigned male at birth. Sex was self-reported. No sex or gender based comparisons were performed, as all participants were biologically male and and gender analysis was not within the scope of this study.
Reporting on race, ethnicity, or other socially relevant groupings	Race data was collected through self-report and included in the demographic characteristics of the study participants. These data were summarized descriptively to provide context on the study population. Race was not controlled as a confounder in the analyses due to the need to adjust for other confounders more directly related to the study's primary objectives such as tumor stage and age.
Population characteristics	Participants in this study were patients diagnosed with prostate cancer about to under androgen deprivation therapy (ADT). Demographic and clinical characteristics were collected, including body composition, functional measures, and patient-reported outcomes. Co-variant related information included age, race, tumor stage, Gleason score, and grade group. After analyzing the data, age and tumor stage were identified as confounders and were adjusted for in the statistical analyses to evaluate how the different outcomes of interest changed after six months of ADT.
Recruitment	Patients were recruited from the VA Puget Sound Health Care System and the University of Washington Urology Clinics through chart review. To minimize selection bias, predefined enrollment criteria were applied consistently to all candidates, ensuring objective inclusion based on medical history. Additionally, recruitment from multiple institutions helped enhance the diversity of the study population.
Ethics oversight	This protocol was approved by the Veteran Affairs Puget Sound Health Care System and University of Washington Institutional Review Boards and their Research and Development Committees and was conducted in compliance with the Declarations of Helsinki and its amendments and the International Conference on Harmonization Guideline for Good Clinical Practices.

Note that full information on the approval of the study protocol must also be provided in the manuscript.

Field-specific reporting

Please select the one below that is the best fit for your research. If you are not sure, read the appropriate sections before making your selection.

[x] Life sciences [ ] Behavioural & social sciences [ ] Ecological, evolutionary & environmental sciences

For a reference copy of the document with all sections, see nature.com/documents/nr-reporting-summary-flat.pdf

Life sciences study design

All studies must disclose on these points even when the disclosure is negative.

Sample size	The sample size was determined based on prior literature assessing changes in lean body mass (LBM) in prostate cancer patients undergoing ADT. A detectable alternative was calculated using a standard deviation of 0.6% for LBM changes. To achieve 90% power at a Type I error probability of 0.0125, a minimum detectable difference of 0.35% in LBM was established. Considering an expected 25% attrition rate, the study aimed to enroll 60 participants, ensuring at least 45 retained at 6-month follow-up to maintain statistical power. The paired nature of repeated measures was leveraged through Generalized Estimating Equations regression models, reducing the impact of static confounders.
Data exclusions	No data was excluded from the study.
Replication	This study is observational and does not involve experimental replication. However, all assessments were conducted using standardized protocols to ensure reproducibility. Patient reported outcome were assessed using validated questionnaires validated in prostate cancer patients.

Randomization	This study was observational, and no randomization was performed. Participants were recruited based on clinical criteria and their initiation of ADT. To account for potential confounders, analyses controlled for key variables such as age and tumor stage.
Blinding	No blinding was performed in this study. Given its observational design, blinding was not applicable to participant allocation. However, standardized procedures were used to minimize bias in muscle function assessments, mitochondrial measurements, and patient reported outcome data collection.

Reporting for specific materials, systems and methods

We require information from authors about some types of materials, experimental systems and methods used in many studies. Here, indicate whether each material, system or method listed is relevant to your study. If you are not sure if a list item applies to your research, read the appropriate section before selecting a response.

Materials & experimental systems

n/a	Involved in the study
☒	☐ Antibodies
☒	☐ Eukaryotic cell lines
☒	☐ Palaeontology and archaeology
☒	☐ Animals and other organisms
☐	☒ Clinical data
☒	☐ Dual use research of concern
☒	☐ Plants

Methods

n/a	Involved in the study
☒	☐ ChIP-seq
☒	☐ Flow cytometry
☒	☐ MRI-based neuroimaging

Clinical data

Policy information about clinical studies

All manuscripts should comply with the ICMJE guidelines for publication of clinical research and a completed CONSORT checklist must be included with all submissions.

Clinical trial registration	This study was not registered on ClinicalTrials.gov or any equivalent registry because it was an observational study without an intervention, and therefore did not meet the criteria for clinical trial registration.
Study protocol	The full study protocol is not publicly available. However, details on study design, data collection, and analysis methods are provided within the manuscript.
Data collection	Recruitment and data collection took place over a 6-month period for each participant, with assessments conducted at baseline, 3 months, and 6 months after initiating ADT. Data collection included body composition (DEXA scans), muscle performance (handgrip strength, stair climbing power, actigraphy, 6-minute walk test, and aerobic capacity (VO2 peak), mitochondrial function (in vivo via 31P MRS/OS and ex vivo via muscle biopsies), and patient-reported outcomes (fatigue, quality of life and function using validated questionnaires). Muscle biopsies were also obtained for proteomic analysis.
Outcomes	The primary outcome measure was the change in lean body mass, measured by DEXA and muscle function as assessed by hand grip strength, stair climbing power, actigraphy, the 6-minute walk test, and VO2 peak. Secondary outcomes included mitochondrial function (measured in vivo by 31P MRS/OS and ex vivo in muscle biopsies), as well as patient-reported outcomes such as fatigue and health-related quality of life (FACT-P, EORTC QLQ-30, and EPIC). Assessments were conducted at baseline, 3 months, and 6 months to evaluate longitudinal changes in response to ADT.

Plants

Seed stocks	*Report on the source of all seed stocks or other plant material used. If applicable, state the seed stock centre and catalogue number. If plant specimens were collected from the field, describe the collection location, date and sampling procedures.*
Novel plant genotypes	*Describe the methods by which all novel plant genotypes were produced. This includes those generated by transgenic approaches, gene editing, chemical/radiation-based mutagenesis and hybridization. For transgenic lines, describe the transformation method, the number of independent lines analyzed and the generation upon which experiments were performed. For gene-edited lines, describe the editor used, the endogenous sequence targeted for editing, the targeting guide RNA sequence (if applicable) and how the editor was applied.*
Authentication	*Describe any authentication procedures for each seed stock used or novel genotype generated. Describe any experiments used to assess the effect of a mutation and, where applicable, how potential secondary effects (e.g. second site T-DNA insertions, mosiacism, off-target gene editing) were examined.*
