# Supplementary material for: Muscle mitochondria, function, mass, and quality of life in prostate cancer during androgen deprivation therapy
Source: Nat Commun. 2026 May 27;17:6884. doi: 10.1038/s41467-026-73542-x (PMC13389078; doi:10.1038/s41467-026-73542-x)
Supplement: Supplementary file 5 — Transparent Peer Review file [file 41467_2026_73542_MOESM5_ESM.pdf]

nature portfolio

Peer Review File

Muscle Mitochondria, Function, Mass, and Quality of Life in Prostate Cancer During Androgen Deprivation Therapy

Corresponding Author: Dr Jose Garcia

This file contains all reviewer reports in order by version, followed by all author rebuttals in order by version.

Version 0:

Reviewer comments:

Reviewer #1

(Remarks to the Author)
The manuscript by Caiero et al reports a prospective observational clinical study studying the effects of androgen deprivation in prostate cancer patients on the relationship between muscle mass, strength and performance and mitochondrial activity. This descriptive work is important as anabolic drugs tested in phase 3 clinical trials in this population have increased muscle mass but not function and quality of life. The manuscript uses an extensive clinical phenotyping of gold standard clinical variables, coupled to mitochondrial function analysis by 31P MRS in vivo in humans and ex vivo in muscle biopsies by respirometry and proteomics. The work is novel, very well controlled and the manuscript is clear and well discussed with important clinical conclusions. However, a limitation is that the study does not provide mechanistic insight in what could be actionable pathways for future interventions and the conclusion on the absence of association of mitochondrial function with ART outcomes should be revisited given some technical limitations and clear proteomic results. Detailed below

One main limitation of the work is that mitochondrial respiration was measured on isolated mitochondria which can detect structural changes in mitochondrial “quality” but is less sensitive than respiration in isolated myofibers. While performing respiration on isolated fibers is not feasible as it needs to be done fresh, the authors could build additional characterization of metabolic fluxes in mitochondria through metabolomics or extrapolation from the proteomics results.

Linked to the above, line 32/33 of the abstract seems an over-interpreted (Mitochondrial function remained unchanged after ADT despite downregulation of mitochondrial related pathways revealed by proteomics). Mitochondrial function is not just ATPmax and state 3 respiration in isolated mitochondria and I feel the results on proteomics do support a role for mitochondrial function in ART. It would be important to answer the following questions to strengthen the different aspects of mitochondrial function from existing results or samples:
- does mitochondrial biogenesis change in PCa and ART? This can be measured with mitochondrial DNA
-does mitophagy change in PCa and ART (eg PINK/Parkin)?

There are several studies in age-related sarcopenia that linked mitochondria and muscle mass and function to NAD levels (Migliavacca et al, Nature Comms 2019; Janssens et al, Nature Aging 2022). Can the authors discuss this parallel and test if NAD levels and NAD metabolism are deregulated in PCa and ART.

Reviewer #2

(Remarks to the Author)
This study aimed to characterize the relationships of muscle endurance, muscle strength, PRO, and EORTC QLQ C-30 with mitochodria function. This study is interesting and provides novel view, bearing potential to improve outcomes in prostate cancer. However, I have concerns regarding statistical analysis which may not optimally present the correlations of the items with mitochondria function. For this, my specific comments are provided below.

* Although the authors had used Spearmans correlations to test correlations between variables, the current Figure 1, 3, and 4 provide limited information. I would suggest the authors to provide scatter plots of several items to provide more

comprehensive view of the correlation of muscle endurance, muscle strength, PRO, and EORTC QLQ C-30 with mitochodria functon(e.g. Maximum ATP at baseline and change and Ex vivo Mitochondrial Respiration) and VO2. Additionally, I also have concerns regarding the Spearmans correlations coefficients of measured items with mitochondria function because most of these values are below 0.6 (Fig. 1, 3, and 4), suggesting weak or moderate correlation despite of statistically significant. Therefore, scatter plots are suggested.

* It would also be nice to compare the muscle endurance, muscle strength, PRO, and EORTC QLQ C-30 between groups stratifying based on mitochondria function and VO2 using median value in the Tables or Figures. This approach can help understand the differences in these items between patients with higher or lower mitochondria function and VO2.

* In the 2.3.4 and 2.3.5 sections: Please briefly explain the clinical meaning of the measures (e.g. Oxygen consumption rate and Maximum ATP synthesis) in these section to help easily interpret your results.

* It is known that skeletal muscle loss during ADT is associated an increased risk of adverse events or all-cause mortality. Preserving muscle mass and strength during ADT is clinically relevant to improve survival outcomes in prostate cancer. Please comment on the potential utility of evaluation of mitochondria function before or during ADT to guide interventions to enhance muscle mass and strength based on the latest references in the discussion section.

Reviewer #3

(Remarks to the Author)
For its strengths
Muscle mass and function are compromised in patients with cancer and these a a detriment to quality of life and associated with poor clinical outcomes. Research in this area is limited by a considerable lack of clinical studies so I was pleased to see an effort in this space. These clinical studies are daunting to conduct, and this research team has managed to do such a study with several aspects of merit compared with prior studies: a clearly defined population with a complete accounting of sampling from within the overall population, inclusion and exclusion criteria to limit extraneous influences, sex-restricted population (male), achievement of skeletal muscle biopsy in a subset of patients to gauge aspects of underlying biology. According to a systematic review on skeletal muscle biopsy in patients with cancer PMID: 31307124, this study meets all relevant quality criteria for this approach. This research team also has established credentials in the measurement of physical functioning. Repeated measures over time is a great strength of this study.

The design and main aims of the study are not clearly presented. The title and introduction go off in a variety of different directions, and based on reading the study aim of a descriptive and correlative study is stated. Many measurements are made – body composition, physical function tests and quality of life measures, biopsy with activity of mitochondrial energetics as well as proteomic analysis. It would be of value to discuss which are the primary and secondary analyses, and to defend they types of analysis planned. This lack of clarity goes through into the discussion, with for example a lot said about loss of appetite, something that is barely a focus of the results section. Various brief references to cachexia across the document don't seem to fit with the overall discourse as the patients are never defined as cachexic or not.

I do think that the analysis plan bears some inspection.
In the body composition domain, the BMI, fat mass and muscle mass are examined by simple univariable correlation with physical functions and questionnaires.

a) About the correlation, it is stated to be Pearson or Spearman according to whether the distribution was normal or not, but there should be some illustration of the plots so that the reader can understand if these are linear relationships or perhaps J-shaped or U-shaped? Why was the correlation not raised to a multivariable level, so that that for example a physical function such as stair climb power could be related simultaneously to age, muscle mass, fat mass, Vo2 and other features?

b) What strikes me as odd about the parade of simple correlations is that muscle mass and fat mass are not completely independent influences on physical functioning, yet there was no attempt to consider the simultaneous fat and muscle mass as composites. For example, sarcopenic obesity is a worse-case functional scenario because the low muscle mass is bearing an unusually high load and the relative muscle fat proportions become important for functions such as walking and climbing stairs, as well as activities of daily living (e.g. PMID: 30377678).

c) Also not clear is why muscle is indexed to height-squared but fat mass is not indexed to height-squared. Considering 2 features (overall weight and muscle mass) indexed to patient height but the fat mass unindexed, at some level does not make sense.

d) Because each individual person is a composite of specific fat and lean proportions, the simple correlation between BMI and physical functions and QOL probably doesn't mean much. A more coherent approach might be to map the muscle and fat proportions (picture an XY plot of muscle and fat) and identify in which quadrant(s) functional deficit and QOL impairment reside.

e) One further thing about BMI, persons who are obese often report impaired QOL, I would not expect overall BMI to have a clear correlation with QOL overall and QOL domain scores that is related to muscle mass or fat mass alone as many other factors play in.

f) An additional complexity to the interpretation of the BMI body composition results is that changes in fat mass and muscle mass go in opposite directions during the anti-androgen therapy, so down the road of sarcopenic obesity or further cementing sarcopenic obesity. However it is also evident that in this period the overall bodyweight of some patients is rising and is others it is falling. Here again the XY plot of muscle change vs fat change might lend some insight into the variation in body composition change.

The physical functioning tests are illuminating but as stated above I wished that the analysis had gone beyond bivariate simple correlations.

As for the questionnaires for QOL, while these are validated, they are not actually validated for the purpose of identifying functional deficits related to muscle mass. One thing that would be helpful to the reader is to discuss that many of the questions have no conceivable relationship with muscle mass and function such as pain, bowel functions, urination, anxiety so maybe it is more useful to focus on the domains and specific questions that are directly relevant to the research questions.

About the mitochondrial measures: These were highly variable (and too variable to detect any differences due to androgen treatment) and I wondered what the possible causes of such variability might be?
I also note some other work related to mitochondria and their functions that were not cited. Perhaps these can be discussed in relation to the findings here:
1: Mallard J, Hucteau E, Bender L, Moinard-Butot F, Rochelle E, Boutonnet L, Grandperrin A, Schott R, Pflumio C, Trensz P, Kalish-Weindling M, Charles AL, Gény B, Favret F, Pivot X, Hureau TJ, Pagano AF. A single chemotherapy administration induces muscle atrophy, mitochondrial alterations and apoptosis in breast cancer patients. J Cachexia Sarcopenia Muscle. 2024 Feb;15(1):292-305. doi: 10.1002/jcsm.13414. Epub 2024 Jan 6. PMID: 38183352; PMCID: PMC10834353.

2: Mallard J, Hucteau E, Charles AL, Bender L, Baeza C, Pélissie M, Trensz P, Pflumio C, Kalish-Weindling M, Gény B, Schott R, Favret F, Pivot X, Hureau TJ, Pagano AF. Chemotherapy impairs skeletal muscle mitochondrial homeostasis in early breast cancer patients. J Cachexia Sarcopenia Muscle. 2022 Jun;13(3):1896-1907. doi: 10.1002/jcsm.12991. Epub 2022 Apr 4. PMID: 35373507; PMCID: PMC9178151.

3: Toth MJ, Voigt TB, Tourville TW, Prior SM, Guigni BA, Schlosberg AV, Smith IB, Forest TJ, Kaufman PA, Wood ME, Rehman H, Dittus K. Effect of neuromuscular electrical stimulation on skeletal muscle size and function in patients with breast cancer receiving chemotherapy. J Appl Physiol (1985). 2020 Jun 1;128(6):1654-1665. doi: 10.1152/japplphysiol.00203.2020. Epub 2020 May 7. PMID: 32378975; PMCID: PMC7311687.

4: Guigni BA, Callahan DM, Tourville TW, Miller MS, Fiske B, Voigt T, Korwin-Mihavics B, Anathy V, Dittus K, Toth MJ. Skeletal muscle atrophy and dysfunction in breast cancer patients: role for chemotherapy-derived oxidant stress. Am J Physiol Cell Physiol. 2018 Nov 1;315(5):C744-C756. doi: 10.1152/ajpcell.00002.2018. Epub 2018 Sep 12. PMID: 30207784; PMCID: PMC6293050.

5: Mijwel S, Cardinale DA, Norrbom J, Chapman M, Ivarsson N, Wengström Y, Sundberg CJ, Rundqvist H. Exercise training during chemotherapy preserves skeletal muscle fiber area, capillarization, and mitochondrial content in patients with breast cancer. FASEB J. 2018 Oct;32(10):5495-5505. doi: 10.1096/fj.201700968R. Epub 2018 May 11. PMID: 29750574.

As to the proteomic analysis – bravo! For the sample collection and first time extensive proteomic analysis in cancer patients' skeletal muscle. This is really valuable information and I was surprised at the cursory presentation of the data. At least in this version authors should consider:
a) Looking more carefully at the “top 10” because many of these are reflections of the same thing. For example, in the different annotation platforms complement and coagulation come up 4-5 times, but this is a single biological domain (not 4 or 5 of them; different platforms, same message). Please relay the top 10 unique biological domains.
b) The proteome is examined overall and in various splits – according to ALM, fatigue, handgrip, VO2 etc, without indicating the overlap of patients in these classes and without making any comparisons across the splits.
Lots of other analyses could be applied to this data set, for example cluster analysis to identify any covarying patterns of protein expression. Information has been under-valued here.

Version 1:

Reviewer comments:

Reviewer #1

(Remarks to the Author)
The authors have addressed all my points with convincing new experiments or discussion of limitations.

Reviewer #2

(Remarks to the Author)
The authors have nicely adderessed the reviewers' concerns.

Reviewer #3

(Remarks to the Author)
I noted some missing information in this version.

*reference number for the data deposited in the Proteomics Identification Database (PRIDE) may be incorrect or embargoed for now- the reference number given in the manuscript does not exist in this data base.
*Supplemental data table with the proteomics results gives the pathway names and the NES and p-value but is missing the protein lists that go along with the pathway results. i.e. which specific proteins are differentially expressed and therefore resulting in the pathway being recognized? Differentially expressed protein lists are missing.

I also found this version difficult to read and follow.
*there is a terminology "adverse effects" "adverse outcomes" "adverse changes" in the text that I am not certain to understand the meaning of. Figure 3 is entitled Predictors of ADT induced adverse effects, however in the context of a correlation matrix its not clear how adverse is defined.
* there is also a term "poor" performers and "non-poor performers". This strikes as odd in the sense that all of the criteria were split at the median, which does not define "poor". For example, the median change in VO2 is -17% (a substantial loss) whereas the median change in 6 minute walk is 1.7% (no change).
*I am also not clear why we are looking at both ALM and fat mass in kg rather than BOTH adjusted to height-square. This would put all of the weight components in comparable units - body mass index, appendicular lean index and fat index. Muscle function and definitions of sarcopenia are most often reported in indexed values. In the current presentation we can't see which patients meet thresholds for sarcopenia because height is not provided.

More Comments and questions

1. Univariable vs multivariable analyses.
I found the thread of unadjusted and adjusted analysis very difficult to follow as the data presented in the main text and in the supplemental section is a mixture of both types of analysis. First, we have univariable correlation at baseline (Figure 1), accompanied by a new Supplemental figure with over 80 plots of those univariable correlations. So the results section launches with an overwhelming mass of univariable correlation. This may or may not be helpful. The multivariable analyses are much more interesting but are relegated to the Supplemental material. Supplemental Table 2 is important as it explains why the approach of univariable correlation is not particularly meaningful: the physical function outcomes are INDEPENDENTLY RELATED TO MULTIPLE PREDICTORS. This appears to be on the right track, however I wonder if you did consider additional items that could relate to physical functioning in the analysis in Suppl Table 2 for example was metastasis tested in all models? Was pain tested for association? Dyspnea?

It will make sense to put the multivariable analysis in the main text and the univariable in the supplemental. I do not understand why this was not done already as in the rebuttal it is stated that the multivariable/GEE is the PRIMARY outcome, so it does make little sense that the primary outcome is reported in the Supplemental materials while secondary analyses should be moved to supplemental.

Figures 3 and 4 are particularly hard to follow. They are adjusted in some way that I did not understand. The titles and legends do not clearly articulate what is different between them.

2. Appended data in excell files included the individual values for all of the physical functioning tests. In order to better understand the correlations I spent some time looking over this data in detail.

A) What is the test-retest validity of all of these measures that would be helpful to know in light of the variability in both of the baseline values and the responses over the six months of androgen deprivation in this population? How much of the variability is measurement error?
B) another aspect of the variability deserving comment is in the 6 month change data. For example, the mean change in peak VO2 was -42%, however a subset of 9 patients increased VO2 up to a maximum value of +73%. What might explain such a large increase in VO2? Likewise, some patients increased their handgrip up to 29%, their 6 minute walk up to 33% and their stair climb power by up to 123%. I wondered if these distributions encompassing a wide spectrum of positive and negative change were taken into account when defining good and poor performers. That categorization was split at the median, but another plausible definition of poor performer could be set at the boundary of >5% loss of functon(for example).
C) furthermore some 6-month change values seem incongruent for example, ID 28 lost fat, lost ALM (14%), lost handgrip (-12%), lost 6 min walk (-26%) but gained VO2 (+45%).

3. One of the things this manuscript hasn't done up to now is address any confounding effects of the underlying disease, stage and rate of progression. This is in part brought to mind by a very recent publication in Nature. Bhatt BJ, et al. Molecular subtypes of human skeletal muscle in cancer cachexia. Nature. 2025 Sep 10. doi: 10.1038/s41586-025-09502-0. PMID: 40931079. This was not published at the time of the first round of revision of the [resent submission but it might be important to consider.
I do think that there might be cancer cachexia going on in some of these patients and that it would be helpful to try and assess its potential impact on the patients' physical function and QOL. First, some of the patients in this sample are losing both weight and muscle mass and have reduced food intake (all diagnostic criterion values for cachexia); some of them may have had sarcopenia at baseline and that should be added. Bhatt et al show that that clinical cachexia is accompanied with some distinctive gene expression signatures. Notably in the current submission, the differential protein expression over six months in the whole cohort (and in its subsets) harmonizes quite strongly with the cachexia signatures in the article by Bhatt & coworkers i.e cellular immune response and leukocytes, complement activation extracellular matrix, hemostasis and coagulation. I guess the question this raises is whether underlying cachexia driven by disease is in part responsible for the changes in protein expression, and in the functional changes. So far the authors have said that cachexia is out of scope of this analysis even though the differential protein expression for these signals is at least as strong as the effects on mitochondrial pathways. It might equally well be suggested that the presence of cachexia in some patients is not surprising because some have metastatic disease, that a subset could be identified as having cachexia by clinical and by biological (protein expression) criteria and that this is a confounder to the present analysis that could easily be controlled for. There is perhaps a hint of this in Suppl Table 2, where the presence of metastasis is independently negatively associated with handgrip strength.

One way in which it might be possible to achieve more clarity in the etiology of the protein signatures would be to look at those that are differentially expressed according to the presence and absence of androgen response elements. If we were to overlay the differential protein expression with a picture of which of those proteins expression is androgen sensitive, we might be able to deconvolute the androgen -specific effects in someway shape or form.

Version 2:

Reviewer comments:

Reviewer #3

(Remarks to the Author)
Thank you for the detailed revisions. Please add information as to the method by which disease response over 6 months was quantified.

Open Access This Peer Review File is licensed under a Creative Commons Attribution 4.0 International License, which permits use, sharing, adaptation, distribution and reproduction in any medium or format, as long as you give appropriate credit to the original author(s) and the source, provide a link to the Creative Commons license, and indicate if changes were made.
In cases where reviewers are anonymous, credit should be given to 'Anonymous Referee' and the source.

The images or other third party material in this Peer Review File are included in the article's Creative Commons license, unless indicated otherwise in a credit line to the material. If material is not included in the article's Creative Commons license and your intended use is not permitted by statutory regulation or exceeds the permitted use, you will need to obtain permission directly from the copyright holder.
To view a copy of this license, visit https://creativecommons.org/licenses/by/4.0/

Point-by-point response to the reviewers' comments

Reviewer #1 (Remarks to the Author); expert in muscle and mitochondria:

The manuscript by Caeiro et al reports a prospective observational clinical study studying the effects of androgen deprivation in prostate cancer patients on the relationship between muscle mass, strength and performance and mitochondrial activity. This descriptive work is important as anabolic drugs tested in phase 3 clinical trials in this population have increased muscle mass but not function and quality of life. The manuscript uses an extensive clinical phenotyping of gold standard clinical variables, coupled to mitochondrial function analysis by 31P MRS in vivo in humans and ex vivo in muscle biopsies by respirometry and proteomics. The work is novel, very well controlled and the manuscript is clear and well discussed with important clinical conclusions. However, a limitation is that the study does not provide mechanistic insight in what could be actionable pathways for future interventions and the conclusion on the absence of association of mitochondrial function with ART outcomes should be revisited given some technical limitations and clear proteomic results. Detailed below

One main limitation of the work is that mitochondrial respiration was measured on isolated mitochondria which can detect structural changes in mitochondrial "quality" but is less sensitive than respiration in isolated myofibers. While performing respiration on isolated fibers is not feasible as it needs to be done fresh, the authors could build additional characterization of metabolic fluxes in mitochondria through metabolomics or extrapolation from the proteomics results.

<u>Response:</u> We appreciate the reviewer's comments and suggestions. We acknowledge the limitations highlighted and have incorporated additional analyses, experiments, and clarifications.

We agree that mitochondrial respiration measured in isolated mitochondria has limitations and is not as sensitive as measuring respiration in isolated myofibers. This is now included as a limitation in the discussion (page 18). As suggested, we have now expanded our proteomics data analysis to extrapolate metabolic changes related to mitochondrial function. Specifically:

- We performed more focused analysis of the proteomics data. Using Gene Set Enrichment Analysis we identified the leading edge proteins that map to mitochondrial pathways, specifically, mitochondrial electron transport, TCA cycle, and mitochondrial biogenesis. The leading edge identifies differentially regulated proteins that are primarily responsible for the enrichment of a given pathway. The average expression of proteins related to these pathways significantly decreased after six months of ADT. This has been added as a new Figure 6A and in the Results on pages 9 and 10, and Discussion page 16.
- Ingenuity Pathway Analysis (IPA) was performed to extrapolate protein data to assess metabolic fluxes in mitochondria. New Figure 6B displays proteins that are downregulated and predicted to be downregulated in the context of their functional roles in mitochondrial metabolism. Proteins from all complexes were downregulated after six months of ADT. This is now depicted in Figure 6B and in the Results on pages 10 and 11 and in the Discussion on page 16. Potential targeting of some of these protein candidates is now included in the Discussion (pages 16 and 17).

Linked to the above, line 32/33 of the abstract seems an over-interpreted (Mitochondrial function remained unchanged after ADT despite downregulation of mitochondrial related pathways revealed by proteomics). Mitochondrial function is not just ATP Max and state 3 respiration in isolated mitochondria and I feel the results on proteomics do support a role for mitochondrial function in ART.

<u>Response:</u> We agree with the reviewer's interpretation of the results and have revised this statement in the abstract which now emphasizes that while direct mitochondrial respiration measures did not change, proteomics data indicate mitochondrial pathway and protein alterations, suggesting mitochondrial dysfunction after ADT.

It would be important to answer the following questions to strengthen the different aspects of mitochondrial function from existing results or samples:
- does mitochondrial biogenesis change in PCa and ART? This can be measured with mitochondrial DNA
-does mitophagy change in PCa and ART (eg PINK/Parkin)?

<u>Response:</u> To assess mitochondrial biogenesis and mitophagy, we extracted RNA from our ten remaining pairs of samples obtained from the skeletal muscle biopsies and performed PCR. We measured PGC1α, TFAM, and NRF1 as markers of mitochondrial biogenesis, while BNIP3, PINK1, and PARK2 were evaluated as markers of mitophagy. These findings have been incorporated into Results section (pages 9 and 10) and into the Discussion (page 15).

There are several studies in age-related sarcopenia that linked mitochondria and muscle mass and function to NAD levels (Migliavacca et al, Nature Comms 2019; Janssens et al, Nature Aging 2022). Can the authors discuss this parallel and test if NAD levels and NAD metabolism are deregulated in PCa and ART.

<u>Response:</u> We appreciate the suggestion and have now measured NAD levels through LC/MS in muscle before and after ADT and found that NAD levels do not change after six months of ADT [Supplemental Figure 6]. We have added this point to the Results (page 11) and Discussion (page 16), noting that despite previous studies linking NAD metabolism to muscle mass and function in age-related sarcopenia (Migliavacca et al., Nature Comms 2019; Janssens et al., Nature Aging 2022), our findings do not support a downregulation of NAD+ levels in the context of ADT over six months.

Reviewer #2 (Remarks to the Author); expert in prostate cancer:

This study aimed to characterize the relationships of muscle endurance, muscle strength, PRO, and EORTC QLQ C-30 with mitochondria function. This study is interesting and provides novel view, bearing potential to improve outcomes in prostate cancer. However, I have concerns regarding statistical analysis which may not optimally present the correlations of the items with mitochondria function. For this, my specific comments are provided below.

We appreciate the reviewers' comments, which have helped us refine our analysis and improve the clarity of our findings. Below, we provide detailed responses.

** Although the authors had used Spearmans correlations to test correlations between variables, the current Figures 1, 3, and 4 provide limited information. I would suggest the authors to provide scatter plots of several items to provide more comprehensive view of the correlation of muscle endurance, muscle strength, PRO, and EORTC QLQ C-30 with mitochondria function (e.g. Maximum ATP at baseline and change and Ex vivo Mitochondrial Respiration) and VO2. Additionally, I also have concerns regarding the Spearmans correlations coefficients of measured items with mitochondria function because most of these values are below 0.6 (Fig. 1, 3, and 4), suggesting weak or moderate correlation despite of statistically significant. Therefore, scatter plots are suggested.*

Response: We appreciate the reviewer's suggestion and have incorporated scatter plots in Supplementary Figures 2-4 to better visualize the relationships between muscle endurance, strength, PRO, EORTC QLQ C-30, mitochondrial function (Maximum ATP at baseline/change, Ex vivo Mitochondrial Respiration), and VO_2 peak.

We acknowledge the reviewer's concerns regarding correlation coefficients below 0.6, indicating weak-to-moderate associations. This limitation is now included in the discussion (page 18).

** It would also be nice to compare the muscle endurance, muscle strength, PRO, and EORTC QLQ C-30 between groups stratifying based on mitochondria function and VO2 using median value in the Tables or Figures. This approach can help understand the differences in these items between patients with higher or lower mitochondria function and VO2.*

Response: We have divided participants into high and low based on the median values of ex vivo mitochondrial respiration, ATP Max, and VO_2 peak comparing muscle endurance, muscle strength, body composition, and patient reported outcomes. We added new tables (Supplementary Tables 3-5) reporting the differences after patient stratification. The findings are now incorporated into the Results (page 7 and 8) and Discussion (page 14).

** In the 2.3.4 and 2.3.5 sections: Please briefly explain the clinical meaning of the measures (e.g. Oxygen consumption rate and Maximum ATP synthesis) in these section to help easily interpret your results.*

Response: We have revised the methods section (page 22 and 23) to include brief explanations of the clinical relevance of each measure and to improve clarity.

Oxygen Consumption Rate (OCR) reflects mitochondrial oxidative phosphorylation capacity of isolated mitochondria, providing insight into mitochondrial quality rather than whole-muscle oxidative capacity. A decline in OCR indicates impaired mitochondrial respiration, which is associated with diminished muscle energy production and is linked to lower exercise capacity and

muscle function. This has been demonstrated in aging populations where reduced mitochondrial function is associated with poor gait stability, reduced muscle strength, and diminished physical performance[1].

Maximum ATP Synthesis Rate, assessed via 31P magnetic resonance spectroscopy (31P-MRS), provides a non-invasive estimate of whole muscle mitochondrial oxidative capacity by measuring the recovery rate of phosphocreatine (PCr) and inorganic phosphate (Pi) following a short exercise. This recovery reflects the efficiency of ATP production through oxidative phosphorylation. Clinically, a reduced ATP Max indicates mitochondrial dysfunction and has been observed in age-related conditions such as sarcopenia[1] and frailty[2].

** It is known that skeletal muscle loss during ADT is associated an increased risk of adverse events or all-cause mortality. Preserving muscle mass and strength during ADT is clinically relevant to improve survival outcomes in prostate cancer. Please comment on the potential utility of evaluation of mitochondria function before or during ADT to guide interventions to enhance muscle mass and strength based on the latest references in the discussion section.*

<u>Response</u>: We have added a comment to page 17 on the use of measuring mitochondrial function to guide future interventions to enhance muscle mass and strength in the Discussion. "Our findings suggest that higher mitochondrial function at baseline is predictive of better maintenance of muscle mass, strength, and patient-reported outcomes during ADT. Assessing mitochondrial function before ADT initiation may help identify individuals at higher risk for muscle mass and function decline, and could guide personalized interventions, such as targeted exercise programs, to preserve muscle health and improve outcomes as those used to counteract functional decline during aging[1]."

Reviewer #3 (Remarks to the Author); expert in cancer-associated cachexia:

For its strengths
Muscle mass and function are compromised in patients with cancer and these a a detriment to quality of life and associated with poor clinical outcomes. Research in this area is limited by a considerable lack of clinical studies so I was pleased to see an effort in this space. These clinical studies are daunting to conduct, and this research team has managed to do such a study with several aspects of merit compared with prior studies: a clearly defined population with a complete accounting of sampling from within the overall population, inclusion and exclusion criteria to limit extraneous influences, sex-restricted population (male), achievement of skeletal muscle biopsy in a subset of patients to gauge aspects of underlying biology. According to a systematic review on skeletal muscle biopsy in patients with cancer PMID: 31307124, this study meets all relevant quality criteria for this approach. This research team also has established credentials in the measurement of physical functioning. Repeated measures over time is a great strength of this study.

The design and main aims of the study are not clearly presented. The title and introduction go off in a variety of different directions, and based on reading the study aim of a descriptive and correlative

study is stated. Many measurements are made – body composition, physical function tests and quality of life measures, biopsy with activity of mitochondrial energetics as well as proteomic analysis. It would be of value to discuss which are the primary and secondary analyses, and to defend they types of analysis planned. This lack of clarity goes through into the discussion, with for example a lot said about loss of appetite, something that is barely a focus of the results section. Various brief references to cachexia across the document don't seem to fit with the overall discourse as the patients are never defined as cachexic or not.

Response: We appreciate the reviewer's comments and agree that clearer presentation is needed and have revised the methods section to clearly distinguish our primary and secondary statistical analyses.

Our study is a prospective, observational longitudinal study evaluating changes in body composition, physical function, PROs, and mitochondrial measures in patients with PCa initiating ADT.

We have clarified in the revised Methods (page 27 and 28) that:

"The primary analysis set a priori assessed changes over time in body composition, physical function, PROs, and mitochondrial function using generalized estimating equations (GEE) with an exchangeable correlation matrix. Models adjusted for baseline values (as reference), tumor stage, and age. Regression coefficients (β) and 95% confidence intervals reflect within-subject changes across timepoints (baseline, 3 months, and 6 months). This method accounts for the repeated measures structure of the data and accommodates partially missing data without requiring imputation.

Secondary analyses, also set a priori, included Spearman or Pearson correlations to assess baseline associations, as well as partial correlations to examine relationships between six-month changes in variables of interest, adjusting for baseline values when significant associations were present at baseline. Statistical significance was defined as 2-sided $\alpha \leq 0.05$."

We selected GEE as our primary analysis method because it appropriately models within-subject change over time in a repeated measures setting accounting for missingness. Exploratory secondary analyses were performed to evaluate predictive relationships and associations among our outcomes of interest, complementing our primary longitudinal approach.

Additionally, we have refocused the Discussion, deemphasizing appetite and cachexia as these were not within the scope of this manuscript.

I do think that the analysis plan bears some inspection.
In the body composition domain, the BMI, fat mass and muscle mass are examined by simple univariable correlation with physical functions and questionnaires.

a) About the correlation, it is stated to be Pearson or Spearman according to whether the distribution was normal or not, but there should be some illustration of the plots so that the reader can understand if these are linear relationships or perhaps J-shaped or U-shaped? Why was the correlation not raised to a multivariable level, so that that for example a physical function such as

stair climb power could be related simultaneously to age, muscle mass, fat mass, Vo2 and other features?

Response: We have now included scatter plots in Supplementary Figures 2-4, depicting relationships between muscle mass, fat mass, BMI, and physical function outcomes (e.g., stair climb power, VO_2, QOL metrics) to better visualize the correlation trends.

Additionally, we expanded our analysis to a multivariable level by performing multiple regression models assessing how age, muscle mass, fat mass, and metastasis simultaneously influence physical function outcomes. These results are now detailed in the Supplementary Table 2, Results (page 7), and Discussion (page 12).

b) What strikes me as odd about the parade of simple correlations is that muscle mass and fat mass are not completely independent influences on physical functioning, yet there was no attempt to consider the simultaneous fat and muscle mass as composites. For example, sarcopenic obesity is a worse-case functional scenario because the low muscle mass is bearing an unusually high load and the relative muscle fat proportions become important for functions such as walking and climbing stairs, as well as activities of daily living (e.g. PMID: 30377678).

Response: We acknowledge the importance of assessing sarcopenic obesity as a factor influencing physical function. To address this, we performed multivariable regression analyses including both muscle and fat mass as predictors to evaluate their independent and combined associations with physical function and quality of life. The results are presented in Supplementary Tables 1-2 and pages 6 and 7.

c) Also not clear is why muscle is indexed to height-squared but fat mass is not indexed to height-squared. Considering 2 features (overall weight and muscle mass) indexed to patient height but the fat mass unindexed, at some level does not make sense.

Response: Based on the reviewer's suggestion, we have now removed the appendicular skeletal muscle index (ASMI), which normalizes appendicular lean mass (ALM) to height squared. We now report only absolute ALM, which aligns with how we report fat mass.

d) Because each individual person is a composite of specific fat and lean proportions, the simple correlation between BMI and physical functions and QOL probably doesn't mean much. A more coherent approach might be to map the muscle and fat proportions (picture an XY plot of muscle and fat) and identify in which quadrant(s) functional deficit and QOL impairment reside.

Response: We agree with the reviewer and to better illustrate individual variability in fat and muscle mass, we created XY scatter plots mapping fat mass against appendicular lean mass (ALM) (figure included for reviewer only). To further understand how these components relate to patient-reported outcomes, we conducted stepwise multivariable linear regression analyses evaluating the combined effects of ALM, fat mass, metastasis, and age on baseline QOL domains. These results, now included in the main text (page 6) and shown in Supplementary Table 1, demonstrate that ALM was the most consistent and significant negative predictor of QOL outcomes.

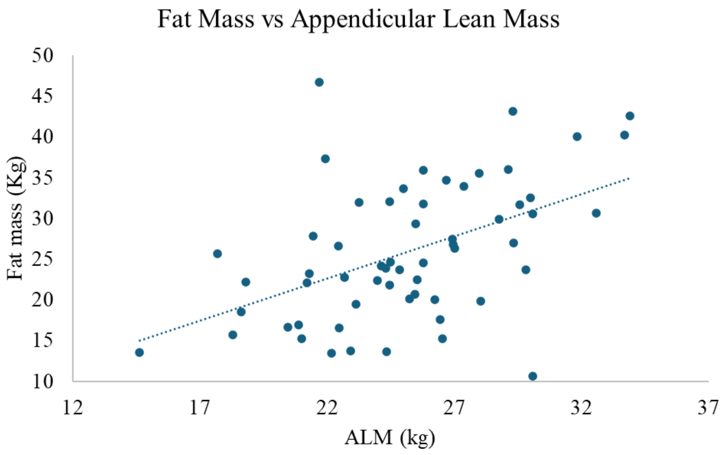

e) One further thing about BMI, persons who are obese often report impaired QOL, I would not expect overall BMI to have a clear correlation with QOL overall and QOL domain scores that is related to muscle mass or fat mass alone as many other factors play in.

Response: We agree that BMI alone may not fully capture the relationship between body composition and QOL. As described above, we addressed this point by evaluating the relationship between body composition and QOL using stepwise multivariable regression. These results are now included in the main text (page 6) and Supplementary Table 1.

f) An additional complexity to the interpretation of the BMI body composition results is that changes in fat mass and muscle mass go in opposite directions during the anti-androgen therapy, so down the road of sarcopenic obesity or further cementing sarcopenic obesity. However it is also evident that in this period the overall bodyweight of some patients is rising and is others it is falling. Here again the XY plot of muscle change vs fat change might lend some insight into the variation in body composition change.

Response: We generated an XY scatter plot of 6-month change in fat mass versus ALM (see below, only for reviewers). While fat and muscle mass changed in opposite directions during ADT, the plot also reveals notable variability. Several individuals gained fat while losing muscle, consistent with a sarcopenic obesity phenotype. A smaller subset showed losses in both fat mass and ALM, while others presented gains in both. These patterns highlight the heterogeneity of body composition responses during treatment.

To further evaluate how changes in body composition relate to outcomes, we conducted stepwise multivariable regression analyses using 6-month change in QOL and functional measures as dependent variables, with age, metastasis and change in ALM, fat mass and VO_2 peak as

independent predictors. These results are now included in the supplementary document (Supplementary Table 10) and page 9.

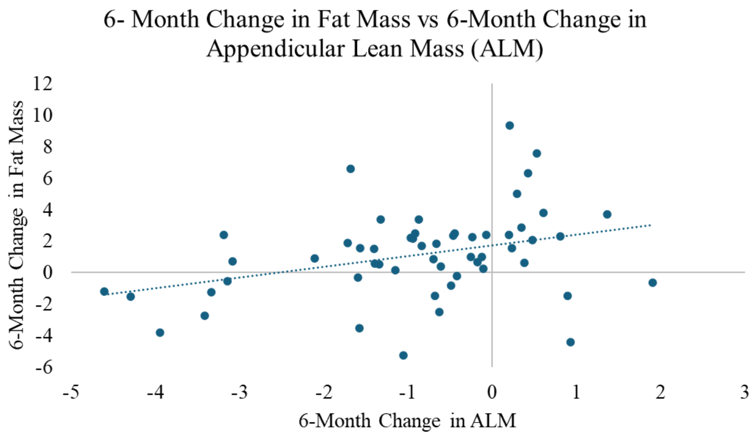

The physical functioning tests are illuminating but as stated above I wished that the analysis had gone beyond bivariate simple correlations.

Response: We appreciate this feedback and have expanded our analysis to move beyond bivariate correlations:

We have incorporated multivariable regression models to assess the combined influence of muscle mass, fat mass, age, and other covariates on physical function outcomes (VO_2 peak, stair climb power, handgrip strength, 6MWT). This can be found in Supplementary Table 1-2.

Subgroup analysis has been added, stratifying patients by high vs. low mitochondrial function and VO_2 to assess how these interact with physical performance. This can be found in Supplementary Table 3-5 and discussed on page 14.

As for the questionnaires for QOL, while these are validated, they are not actually validated for the purpose of identifying functional deficits related to muscle mass. One thing that would be helpful to the reader is to discuss that many of the questions have no conceivable relationship with muscle mass and function such as pain, bowel functions, urination, anxiety so maybe it is more useful to focus on the domains and specific questions that are directly relevant to the research questions.

Response: We agree that not all QOL questionnaire components are directly relevant to muscle function and physical performance. To refine this aspect of our analysis:

We have focused our interpretation on QOL domains most relevant to muscle mass and function, including Physical Functioning, Fatigue, Role Functioning, and Global Health Status, removing pain, bowel functions, and other non-relevant questions. These changes are reflected in the updated figures and are acknowledged as a limitation on page 18.

About the mitochondrial measures: These were highly variable (and too variable to detect any differences due to androgen treatment) and I wondered what the possible causes of such variability might be?
I also note some other work related to mitochondria and their functions that were not cited. Perhaps these can be discussed in relation to the findings here:

1: Mallard J, Hucteau E, Bender L, Moinard-Butot F, Rochelle E, Boutonnet L, Grandperrin A, Schott R, Pflumio C, Trensz P, Kalish-Weindling M, Charles AL, Gény B, Favret F, Pivot X, Hureau TJ, Pagano AF. A single chemotherapy administration induces muscle atrophy, mitochondrial alterations and apoptosis in breast cancer patients. J Cachexia Sarcopenia Muscle. 2024 Feb;15(1):292-305. doi: 10.1002/jcsm.13414. Epub 2024 Jan 6. PMID: 38183352; PMCID: PMC10834353.

2: Mallard J, Hucteau E, Charles AL, Bender L, Baeza C, Pélissie M, Trensz P, Pflumio C, Kalish-Weindling M, Gény B, Schott R, Favret F, Pivot X, Hureau TJ, Pagano AF. Chemotherapy impairs skeletal muscle mitochondrial homeostasis in early breast cancer patients. J Cachexia Sarcopenia Muscle. 2022 Jun;13(3):1896-1907. doi: 10.1002/jcsm.12991. Epub 2022 Apr 4. PMID: 35373507; PMCID: PMC9178151.

3: Toth MJ, Voigt TB, Tourville TW, Prior SM, Guigni BA, Schlosberg AV, Smith IB, Forest TJ, Kaufman PA, Wood ME, Rehman H, Dittus K. Effect of neuromuscular electrical stimulation on skeletal muscle size and function in patients with breast cancer receiving chemotherapy. J Appl Physiol (1985). 2020 Jun 1;128(6):1654-1665. doi: 10.1152/japplphysiol.00203.2020. Epub 2020 May 7. PMID: 32378975; PMCID: PMC7311687.

4: Guigni BA, Callahan DM, Tourville TW, Miller MS, Fiske B, Voigt T, Korwin-Mihavics B, Anathy V, Dittus K, Toth MJ. Skeletal muscle atrophy and dysfunction in breast cancer patients: role for chemotherapy-derived oxidant stress. Am J Physiol Cell Physiol. 2018 Nov 1;315(5):C744-C756. doi: 10.1152/ajpcell.00002.2018. Epub 2018 Sep 12. PMID: 30207784; PMCID: PMC6293050.

5: Mijwel S, Cardinale DA, Norrbom J, Chapman M, Ivarsson N, Wengström Y, Sundberg CJ, Rundqvist H. Exercise training during chemotherapy preserves skeletal muscle fiber area, capillarization, and mitochondrial content in patients with breast cancer. FASEB J. 2018 Oct;32(10):5495-5505. doi: 10.1096/fj.201700968R. Epub 2018 May 11. PMID: 29750574.

Response: We acknowledge the high variability of mitochondria measurements. Studies have reported variability in both in vivo[3] and ex-vivo[4] mitochondrial respiration measurements. We have added the following points to the limitation on page 18.

Larson-Meyer et al. reported coefficients of variation as high as 19% and noted greater reliability at higher workloads, likely due to complete muscle fiber recruitment with MRS measurements. Given our population of older prostate cancer patients undergoing ADT, lower exercise tolerance may have contributed to reduced recruitment of muscle fibers and signal variability[4]. We acknowledge this as a limitation of our study.

Ex vivo respiration measures are known to exhibit variability, even under standardized conditions, due to factors such as oxygen or substrate depletion at high respiratory rates, differences in mitochondrial yield, and inter-assay fluctuations[5]. We have also added the suggested references to the manuscript to further discussion of our results.

As to the proteomic analysis – bravo! For the sample collection and first time extensive proteomic analysis in cancer patients' skeletal muscle. This is really valuable information and I was surprised at the cursory presentation of the data. At least in this version authors should consider:
a) Looking more carefully at the "top 10" because many of these are reflections of the same thing. For example, in the different annotation platforms complement and coagulation come up 4-5 times, but this is a single biological domain (not 4 or 5 of them; different platforms, same message). Please relay the top 10 unique biological domains.
b) The proteome is examined overall and in various splits – according to ALM, fatigue, handgrip, VO2 etc, without indicating the overlap of patients in these classes and without making any comparisons across the splits.
Lots of other analyses could be applied to this data set, for example cluster analysis to identify any covarying patterns of protein expression. Information has been under-valued here.

Response: We thank the referee for appreciating our proteomics analyses and have followed the recommendation for deeper investigation of this dataset. To strengthen the proteomics analysis, we conducted a network-based analysis of the differentially enriched pathways in participants treated with ADT. The new Figure 5A illustrates the diverse processes modulated by ADT in the skeletal muscle proteome in these patients. In response to the excellent suggestion by the reviewer, we have redefined the top up and downregulated pathways by combining redundant annotations (e.g., coagulation and complement pathways) to better capture the diversity of unique biological domains dysregulated by ADT (Figure 5B). Furthermore, we have performed more granular analyses using IPA to link our proteomic results with functional mitochondrial perturbations associated with ADT exposure (new Figure 6). In response to the reviewer, we also clarified the overlap of patients in different subgroup analyses (e.g., stratified by ALM, fatigue, handgrip, VO_2), now detailed in Figure 7A, and clustered enriched proteomic-derived pathways in poor performers and non-poor performers (Figure 7B).

1 Grevendonk, L. *et al*. Impact of aging and exercise on skeletal muscle mitochondrial capacity, energy metabolism, and physical function. *Nat Commun* **12**, 4773 (2021). https://doi.org/10.1038/s41467-021-24956-2
2 Coen, P. M. *et al*. Skeletal muscle mitochondrial energetics are associated with maximal aerobic capacity and walking speed in older adults. *J Gerontol A Biol Sci Med Sci* **68**, 447-455 (2013). https://doi.org/10.1093/gerona/gls196

3 Jacques, M. *et al.* Mitochondrial respiration variability and simulations in human skeletal muscle: The Gene SMART study. *FASEB J* **34**, 2978-2986 (2020). https://doi.org/10.1096/fj.201901997RR

4 Larson-Meyer, D. E., Newcomer, B. R., Hunter, G. R., Hetherington, H. P. & Weinsier, R. L. 31P MRS measurement of mitochondrial function in skeletal muscle: reliability, force-level sensitivity and relation to whole body maximal oxygen uptake. *NMR Biomed* **13**, 14-27 (2000). https://doi.org/10.1002/(sici)1099-1492(200002)13:1<14::aid-nbm605>3.0.co;2-0

5 Divakaruni, A. S. & Jastroch, M. A practical guide for the analysis, standardization and interpretation of oxygen consumption measurements. *Nat Metab* **4**, 978-994 (2022). https://doi.org/10.1038/s42255-022-00619-4

Point-by-point response to the reviewer 3 comments.

We thank the reviewer for the detailed comments and critiques. We have now carefully addressed them and revised the manuscript accordingly. Our responses are provided point by point below.

**reference number for the data deposited in the Proteomics Identification Database (PRIDE) may be incorrect or embargoed for now- the reference number given in the manuscript does not exist in this database.*

Response: Please find the account details to the Proteomics Identification Database (PRIDE):

Username: reviewer_pxd061070@ebi.ac.uk

Password: VDeXi30Nnuv4

The link to access the data is https://www.ebi.ac.uk/pride/login. Please use the standard Log In tab (not the Login using Reviewer Token). The data will become publicly accessible at time of publication as recommended by PRIDE.

**Supplemental data table with the proteomics results gives the pathway names and the NES and p-value but is missing the protein lists that go along with the pathway results. i.e. which specific proteins are differentially expressed and therefore resulting in the pathway being recognized? Differentially expressed protein lists are missing.*

Response: We have expanded the proteomics supplemental data to include the leading-edge protein lists contributing to each enriched pathway (Provided in Supplemental Document 2). This enhancement allows readers to directly link differentially expressed proteins to the corresponding pathway enrichments.

**there is a terminology "adverse effects" "adverse outcomes" "adverse changes" in the text that I am not certain to understand the meaning of. Figure 3 is entitled Predictors of ADT induced adverse effects, however in the context of a correlation matrix its not clear how adverse is defined.*

** there is also a term "poor" performers and "non-poor performers". This strikes as odd in the sense that all of the criteria were split at the median, which does not define "poor". For example, the median change in VO2 is -17% (a substantial loss) whereas the median change in 6 minute walk is 1.7% (no change).*

Response: We agree with the reviewer and have revised the terminology throughout the manuscript for clarity and now replaced “adverse effects/outcomes with “side effects”. Moreover, we replaced “poor-performers/non-poor performers” with “above median change” and “below median change”, to better elucidate the cut-offs used. We utilized the median to divide participants into two equal groups, as there is no validated cutoff value for defining performance or muscle mass decline in this population. Using the median ensures balanced sample sizes and avoids introducing arbitrary thresholds.

**I am also not clear why we are looking at both ALM and fat mass in kg rather than BOTH adjusted to height-square. This would put all of the weight components in comparable units - body mass index, appendicular lean index and fat index. Muscle function and definitions of*

sarcopenia are most often reported in indexed values. In the current presentation we can't see which patients meet thresholds for sarcopenia because height is not provided.

Response: We appreciate the reviewer for raising this point. We have performed both analyses and the results obtained for ASMI were very similar to those for absolute ALM, indicating that normalization by height did not affect the findings (please see the image below, only for reviewer). Moreover, ASMI, evaluated using GEE and adjusted for tumor stage and age, showed significant changes at three and six months (B = -0.13, p = 0.021 at three months; B = -0.30, p < 0.001 at six months), similar to the changes observed in ALM. We reported ALM and fat mass in Kg in the main text as it facilitates interpretation given that height-adjusted ALM and fat mass are not used in clinical settings. Moreover, in cohorts with limited variability in height, as in ours (mean: 176.5 cm, SD: 7.3 cm), and when evaluating changes over 6 months when height is a constant in adults, normalization provides little additional information to the analysis and interpretation of the data.

Baseline

Baseline						FACT-P		EORTC QLQ C-30				
	BMI	Fat Mass	SCP	HGS	Maximum ATP[B]	PWB	PCa AC	PF	RF	SF	Fatigue	Dyspnea
ALM	.64**	.48**	.34*	.36**	-.41*	-.42**	-.28*	-.26*	-.27*	-.44**	.33*	.36**
ASMI	.69**	.38**	.29*	.37**	-.31	-.34**	-.29*	-.20	-.27*	-.35**	0.26	.36**

Six-month Change

Six-month Change				FACT-P	EORTC QLQ C-30		
	Fat Mass	6MWT	SCP	Total	PF	CF	Appetite loss
ASMI	.32**	.41**	.36**	.28*	.34*	.29*	-.53**
ALM	.32**	.41**	.38**	.28*	.34*	.29*	-.60**

1. Univariable vs multivariable analyses.
I found the thread of unadjusted and adjusted analysis very difficult to follow as the data presented in the main text and in the supplemental section is a mixture of both types of analysis. First, we have univariable correlation at baseline (Figure 1), accompanied by a new Supplemental figure with over 80 plots of those univariable correlations. So the results section launches with an overwhelming mass of univariable correlation. This may or may not be helpful. The multivariable analyses are much more interesting but are relegated to the Supplemental material. Supplemental Table 2 is important as it explains why the approach of univariable correlation is not particularly meaningful: the physical function outcomes are

INDEPENDENTLY RELATED TO MULTIPLE PREDICTORS. This appears to be on the right track, however I wonder if you did consider additional items that could relate to physical functioning in the analysis in Suppl Table 2 for example was metastasis tested in all models? Was pain tested for association? Dyspnea?

Response: All multivariable regression models were adjusted for metastasis, as specified in the figure legend. However, dependent variables with significant associations are shown in the tables for more clarity. Due to sample size constraints, pain and dyspnea were excluded from the model to focus on predictors with the greatest anticipated impact. In our models (N = 56-59), we included five predictors (age, ALM, fat mass, metastasis, and either VO_2 peak or HGS), which aligns with current recommendations to maintain at least ten participants for every predictor in the model [1].

The new supplemental figure with over 80 plots of those univariable correlations was requested by other reviewers. We opted to include it as we think some readers will find it useful.

It will make sense to put the multivariable analysis in the main text and the univariable in the supplemental. I do not understand why this was not done already as in the rebuttal it is stated that the multivariable/GEE is the PRIMARY outcome, so it does make little sense that the primary outcome is reported in the Supplemental materials while secondary analyses should be moved to supplemental.

Response: We agree with the reviewer's comment and have revised the Results section accordingly. Supplemental table 1 and 2 have now been moved to the in the main text as Table 2 and 3 respectively. In turn, the univariable analyses (Figure 1, 3, and 4) have been relocated to the Supplemental Material to improve clarity and focus. Figure titles, legends, and corresponding text have also been updated to clearly distinguish between univariable and multivariable analyses.

Figures 3 and 4 are particularly hard to follow. They are adjusted in some way that I did not understand. The titles and legends do not clearly articulate what is different between them.

Response: We appreciate the reviewer's comment and agree that the figure legends are not sufficiently clear. The original Figures 3 and 4 (now supplemental Figure 3 and 4) present partial Spearman correlations that were performed only for variable pairs that showed significant associations, specifically between baseline values and 6-month change in outcomes (supplemental Figure 3) and between 6 months changes in outcomes (supplemental Figure. 4). In addition, these correlations were only adjusted when the same variables were significantly correlated at baseline. For example, if baseline or change in variable X was significantly associated with change in variable Y, and X and Y were also correlated at baseline, we conducted a partial Spearman correlation adjusting for baseline Y. The figure legends have been revised to clarify this and now read:

Figure 3: "Spearman correlations were calculated between baseline values and 6-month changes for all variable pairs. For pairs showing significant associations, partial Spearman correlations were subsequently performed, adjusting for the corresponding baseline variable—provided those variables were also significantly correlated at baseline."
Figure 4:"Spearman correlations were calculated between 6-month changes for all variable

pairs. For pairs showing significant associations, partial Spearman correlations were subsequently performed, adjusting for the corresponding baseline variable—provided those variables were also significantly correlated at baseline”

What is the test–retest validity of the functional measures? Some changes appear inconsistent (e.g., gains in VO_2 despite losses in other metrics). Also, why define “poor performers” by median split rather than a clinically meaningful threshold such as >5% loss?

B) another aspect of the variability deserving comment is in the 6 month change data. For example, the mean change in peak VO2 was -42%, however a subset of 9 patients increased VO2 up to a maximum value of +73%. What might explain such a large increase in VO2? Likewise, some patients increased their handgrip up to 29%, their 6 minute walk up to 33% and their stair climb power by up to 123%. I wondered if these distributions encompassing a wide spectrum of positive and negative change were taken into account when defining good and poor performers. That categorization was split at the median, but another plausible definition of poor performer could be set at the boundary of >5% loss of function (for example).

C) furthermore some 6-month change values seem incongruent for example, ID 28 lost fat, lost ALM (14%), lost handgrip (-12%), lost 6 min walk (-26%) but gained VO2 (+45%).

Response: VO_2 peak has shown to have excellent test-retest variability in patients with cardiac or respiratory disease [2], with coronary artery disease [3], and in men with prostate cancer with moderate with-in subject variability [4]. Similarly, 6MWT [5], HGS [6], and SCP [7] have also shown high test re-test reliability, supporting the reliability of these functional measures.

We re-checked our data and confirmed that the observed variability is accurate. Moreover, our statistical approach, which involved testing all variables for normality and applying log-transformation when appropriate, helped minimize the influence of outliers in our results. We acknowledge that some participants presented large improvements while others worsened. The design of our study does not allow us to establish the reasons for this variability and this limitation is now included in the discussion and future directions. As mentioned above, we used the median to divide participants into two equal groups, as there is no validated cutoff value for defining performance or muscle mass decline in this population. Using the median ensures balanced sample sizes and avoids introducing arbitrary thresholds that lack clinical validation in this population.

3. One of the things this manuscript hasn’t done up to now is address any confounding effects of the underlying disease, stage and rate of progression. This is in part brought to mind by a very recent publication in Nature. Bhatt BJ, et al. Molecular subtypes of human skeletal muscle in cancer cachexia. Nature. 2025 Sep 10. doi: 10.1038/s41586-025-09502-0. PMID: 40931079. This was not published at the time of the first round of revision of the [resent submission but it might be important to consider.

I do think that there might be cancer cachexia going on in some of these patients and that it would be helpful to try and assess its potential impact on the patients' physical function and QOL. First, some of the patients in this sample are losing both weight and muscle mass and have reduced food intake (all diagnostic criterion values for cachexia); some of them may have had sarcopenia at baseline and that should be added. Bhatt et al show that that clinical

cachexia is accompanied with some distinctive gene expression signatures. Notably in the current submission, the differential protein expression over six months in the whole cohort (and in its subsets) harmonizes quite strongly with the cachexia signatures in the article by Bhatt & coworkers i.e cellular immune response and leukocytes, complement activation extracellular matrix, hemostasis and coagulation. I guess the question this raises is whether underlying cachexia driven by disease is in part responsible for the changes in protein expression, and in the functional changes. So far the authors have said that cachexia is out of scope of this analysis even though the differential protein expression for these signals is at least as strong as the effects on mitochondrial pathways. It might equally well be suggested that the presence of cachexia in some patients is not surprising because some have metastatic disease, that a subset could be identified as having cachexia by clinical and by biological (protein expression) criteria and that this is a confounder to the present analysis that could easily be controlled for. There is perhaps a hint of this in Suppl Table 2, where the presence of metastasis is independently negatively associated with handgrip strength.

Response: We thank the reviewer for the insightful comment and for highlighting this very relevant recent publication. All participants in our study had advanced adenocarcinoma of the prostate requiring ADT, providing a homogenous sample population. The presence of metastasis (Stage IV disease) was included in our models of regression as an independent variable to account for potential confounding effects related to disease severity. Furthermore, there was no significant tumor progression during the six months that the patients were evaluated.

Cancer cachexia, as defined by Fearon, et al. (https://pubmed.ncbi.nlm.nih.gov/21296615/), is "defined by an ongoing loss of skeletal muscle mass that leads to progressive functional impairment". Bhatt at al recently reported on the association between weight and muscle mass loss and the muscle RNAome but do not provide insight into its association with "functional impairment", being muscle function or patient-reported outcomes such as anorexia or fatigue. We have added to the discussion the following paragraph to address this point (page 16):

"GSEA also highlighted upregulation of ECM remodeling potentially indicating increased muscle fibrosis and activation of inflammation-related pathways. This is consistent with prior findings in hypogonadal men where testosterone administration reduced serum inflammatory cytokines (49) and suggest that ADT may promote a pro-inflammatory state. Moreover, the increased cardiovascular risk associated with ADT (50) aligns with our observation of upregulated coagulation pathways. Similar molecular signatures have also been reported recently in a mix group of pancreatic and advanced colon cancer patients with muscle and weight loss, including activation of immune, extracellular matrix, and coagulation pathways (*Bhatt et al.*, *Nature*, 2025). However, the functional consequences of these molecular changes in muscle were not assessed in that study, and thus, a direct link between these pathways and muscle function or PROs remains to be established in those settings.

One way in which it might be possible to achieve more clarity in the etiology of the protein signatures would be to look at those that are differentially expressed according to the presence and absence of androgen response elements. If we were to overlay the differential protein expression with a picture of which of those proteins expression is androgen sensitive, we might be able to deconvolute the androgen -specific effects in someway shape or form.

Response: We thank the referee for raising this issue. We agree that it is challenging to reliably distinguish proteomic changes due to direct ADT effect from those due to subsequent consequences of treatment and our study was not designed to discriminate between the two possibilities. However, following the suggestion provided, we performed exploratory analyses to determine whether there was an over-representation of proteins influenced by ADT in our proteomic data. To do this, we first searched published resources to identify genes whose expression is influenced by the presence of an androgen response element (ARE). Combining ChIP-seq and transcriptomics, Wilson et al [8] defined a set of genes differentially regulated through ARE fullsite motifs. A limitation of this dataset is that it is derived from prostate cancer cell lines and may therefore not be as relevant to muscle. We complemented this list by leveraging the Harmonize resource (https://maayanlab.cloud/Harmonizome/) to identify physically interacting proteins with the androgen receptor (Pathway Commons and Hub protein-protein databases). Next we overlaid these candidates with our muscle proteomics data to determine whether proteins mapped to the leading edge of our Gene Set Enrichment Analysis (GSEA) were over-represented for presence of ARE or interaction with the androgen receptor using the hypergeometric test. We did not observe a statistically significant enrichment of proteins (P-value = 0.84) with ARE sites, however there was a significant enrichment in our GSEA-associated proteins for those directly interacting with the androgen receptor (P = 0.002). This is consistent with our overall finding that ADT elicits significant proteomics alterations in muscle and suggests that proteins known to interact with the androgen receptor are over-represented in driving this signal. Given the exploratory nature of this analysis, we have not included it in the revision. In future work, we plan to design our studies to more robustly assess the direct effects of ADT on gene and protein expression.

References

1) Seabrook JA. Powering Nutrition Research: Practical Strategies for Sample Size in Multiple Regression. Nutrients. 2025;17(16):2668.

2) Barron A, Dhutia N, Mayet J, Hughes AD, Francis DP, Wensel R. Test-retest repeatability of cardiopulmonary exercise test variables in patients with cardiac or respiratory disease. Eur J Prev Cardiol. 2014;21(4):445-53.

3) Coeckelberghs E, Buys R, Goetschalckx K, Pattyn N, Vanhees L, Cornelissen V. Test-Retest Reliability of Maximal and Submaximal Gas Exchange Variables in Patients With Coronary Artery Disease. J Cardiopulm Rehabil Prev. 2016;36(4):263-9.

4) Scott JM, Hornsby WE, Lane A, Kenjale AA, Eves ND, Jones LW. Reliability of maximal cardiopulmonary exercise testing in men with prostate cancer. Med Sci Sports Exerc. 2015;47(1):27-32.

5) Schmidt K, Vogt L, Thiel C, Jäger E, Banzer W. Validity of the six-minute walk test in cancer patients. Int J Sports Med. 2013;34(7):631-6

6) de Almeida Marques R, de Souza VF, do Rosario TC, da Silva Garcia MRP, Pereira TSS, Marques-Rocha JL, Guandalini VR. Agreement between maximum and mean handgrip strength measurements in cancer patients. PLoS One. 2022;17(7):e0270631.

7) Gagliano-Jucá T, Li Z, Pencina KM, Traustadóttir T, Travison TG, Woodhouse L, Basaria S, Tsitouras PD, Harman SM, Bhasin S, Storer TW. The stair climb power test as an efficacy outcome in randomized trials of function promoting therapies in older men. J Gerontol A Biol Sci Med Sci. 2020;75(6):1167-75

8) Wilson S, Qi J, Filipp FV. Refinement of the androgen response element based on ChIP-Seq in androgen-insensitive and androgen-responsive prostate cancer cell lines. Sci Rep. 2016;6:32611

Response to reviewer:

Thank you for the detailed revisions. Please add information as to the method by which disease response over 6 months was quantified.

Response: We appreciate the reviewer's comment. We have added the following statement to the Methods section on page 21 to clarify how disease response over 6 months was assessed: "Disease response was evaluated through review of clinical records, including urology and oncology notes, imaging and laboratory values, subject interviews, and, when clinically indicated, a physical exam at the three- and six-month follow-up visit."

Response to Reviewer 3:

One of the things this manuscript hasn't done up to now is address any confounding effects of the underlying PCa, stage and rate of progression.

Response: We thank the reviewer for the insightful comment. All participants in our study had advanced adenocarcinoma of the prostate requiring ADT, providing a homogenous sample population. The presence of metastasis (Stage IV disease) was included in our models of regression as an independent variable to account for potential confounding effects related to disease severity. Furthermore, there was no significant tumor progression during the six months that the patients were evaluated. We have now expanded the Methods section to provide a more explicit and detailed description of how tumor response was assessed (Page 21).

"Tumor response was evaluated through review of clinical records, including but not limited to urology and oncology notes, imaging and laboratory values, subject interviews, and, when clinically indicated, a physical exam at the three- and six-month follow-up visit. Specifically, when available, specific markers such as prostate-specific antigen or alkaline phosphatase were extracted from the medical record to evaluate tumor progression, lack of response to therapy, or metastatic disease. Other laboratory values such as hepatic and renal function were collected as well. Radiological reports (including CT, MRI) were reviewed for documentation of new or progressing metastatic disease, such as to the bones or lymph nodes, interval growth of known lesions, or radiographic progression. Moreover, clinical notes from oncology and/or urology visits were reviewed for documentation of disease-related symptom progression, changes in treatment strategy due to suspected progression, or physician assessed treatment response."

A very recent publication in Nature. Bhatt BJ, et al. Molecular subtypes of human skeletal muscle in cancer cachexia. Nature. 2025 Sep 10. doi: 10.1038/s41586-025-09502-0. PMID: 40931079. This was not published at the time of the first round of revision of the [resent submission but it might be important to consider. I do think that there might be cancer cachexia going on in some of these patients and that it would be helpful to try and assess its potential impact on the patients' physical function and QOL. First, some of the patients in this sample are losing both weight and muscle mass and have reduced food intake (all diagnostic criterion values for cachexia); some of them may have had sarcopenia at baseline and that should be added. Bhatt et al show that that clinical cachexia is accompanied with some distinctive gene expression signatures. Notably in the current submission, the differential protein expression over six months in the whole cohort (and in its subsets) harmonizes quite strongly with the cachexia signatures in the article by Bhatt & coworkers i.e cellular immune response and leukocytes, complement activation extracellular matrix, hemostasis and coagulation. I guess the question this raises is whether underlying cachexia driven by disease is in part responsible for the changes in protein expression, and in the functional changes. So far the authors have said that cachexia is out of scope of this analysis even though the differential protein expression for these

signals is at least as strong as the effects on mitochondrial pathways. It might equally well be suggested that the presence of cachexia in some patients is not surprising because some have metastatic disease, that a subset could be identified as having cachexia by clinical and by biological (protein expression) criteria and that this is a confounder to the present analysis that could easily be controlled for. There is perhaps a hint of this in Suppl Table 2, where the presence of metastasis is independently negatively associated with handgrip strength.

Response: Although not the focus of our study, we appreciate the opportunity to discuss the construct of cancer cachexia in this setting. Cancer cachexia, as defined by Fearon, et al. (1), is "an ongoing loss of skeletal muscle mass that leads to progressive functional impairment". For its diagnosis, this definition relies primarily on weight loss, typically defined as >5% weight loss over six months or >2% in individuals with low BMI. The study by Bhatt BJ, et al. did not measure functional impairment and, as such, it is not known if these patients met the Fearon definition of cachexia despite their weight or muscle mass loss.

Most clinical trials only rely on BMI and BMI changes to enroll cancer patients with cachexia because muscle mass is not assessed outside of research studies. This is problematic because neither muscle mass nor body weight are considered clinically meaningful outcomes, defined as measures of how patients feel, function or survive (2). It is critical that studies include these clinically meaningful outcomes as muscle mass and muscle function follow distinct and divergent trajectories in cancer patients, with accumulating evidence demonstrating that functional decline can occur independently of, and often precedes, muscle mass loss (3, 4). To date, cancer cachexia phase III clinical trials have failed to gain regulatory approval despite showing increases in muscle mass because they did not show corresponding improvements in physical function or patient-reported outcomes (5, 6). Regulatory agencies including the U.S. Food and Drug Administration (FDA) and the European Medicines Agency (EMA) emphasize the need for improvements in clinically meaningful outcomes that reflect how patients feel or function rather than changes in muscle mass alone as the latter one is not considered a clinically meaningful and approvable endpoint (7).

Androgen deprivation therapy (ADT) in prostate cancer patients leads to increases in body weight and to severe fatigue and decreases in muscle function that is out of proportion to the loss in muscle mass they experience (8, 9). Hence, framing the changes observed in this setting as "cachexia" will not accurately reflect the phenotype and symptom burden in these patients and will lead to misinterpretation of the underlying biology of muscle alterations in this population. Consistent with this, exploratory follow-up analysis in our study showed that only seven participants became cachectic based on the above criteria after six months of ADT, while four individuals who met these cachexia criteria at baseline, no longer met those criteria at follow-up.

In summary, applying cachexia criteria in this population will misclassify patients and confound treatment-induced metabolic changes with tumor-driven cachexia. Additional exploratory analyses and complex interpretation and discussion that extend beyond the scope of the current

manuscript, whose predefined goals were to characterize changes in body composition, muscle function, patient-reported outcomes, and mitochondrial biology during ADT, would be needed.

Other methodological concerns also arise with analyzing the data based on weight or muscle mass loss. First, the study already captures the core components of cachexia including body weight, ALM, fat mass, physical function, appetite loss, and fatigue as continuous variables. Hence, there would be a complete overlap between cachexia criteria and the existing outcomes reported and included in the *a priori* study design. In other words, including ALM and body weight both as continuous predictors and as components of a categorical cachexia variable within the same analytical framework will introduce multicollinearity. When a variable appears simultaneously as an independent predict. In this "double dipping", the same variables would be used twice, making it impossible to determine whether any observed association is driven by the individual measure or by its contribution to the categorical definition of cachexia. For this reason, we believe the continuous variables as specified in the *a priori* study design provide a more statistically valid and interpretable approach. Second, given the low prevalence of weight loss in this setting, the study will be grossly underpowered to establish meaningful comparisons between groups.

We expanded our discussion to further highlight the importance of functional outcomes and clinically meaningful endpoints when interpreting the findings of our study as follows:

"This suggests that muscle endurance may be more clinically relevant than ALM in advanced PCa, as it is associated with better physical function and lower symptom burden. Moreover, this is important to consider when developing therapeutic interventions and selecting primary outcomes in patients with PCa. As in cancer cachexia, a condition defined by Fearon, et al. "an ongoing loss of skeletal muscle mass that leads to progressive functional impairment" (1), phase III clinical trials (5, 6) have reported increases in muscle mass without corresponding improvements in physical function or patient-reported outcomes, which prevented regulatory approval. Regulatory agencies including the U.S. Food and Drug Administration emphasize the need for improvements in clinically meaningful outcomes that reflect how patients feel or function rather than changes in muscle mass alone as the latter one is not considered a clinically meaningful and approvable endpoint (7)." (Page 13)

"Our results highlight the differential impact of ADT on a range of physical function outcomes, emphasizing the need for selecting appropriate tools in future clinical trials involving PCa patients undergoing ADT. This is also important in conditions such as cancer cachexia, where diagnostic criteria rely largely on weight loss (50) and therefore underestimate the burden experienced by PCa patients undergoing ADT, as illustrated by our findings and a body of literature showing disproportionate worsening in PROs and physical function despite modest decreases in muscle mass and no weight loss (8, 9)." (Page 15)

Regarding the Bhatt et al. study, we acknowledge the similarities between molecular signatures in both studies as follows:

"Similar molecular signatures have also been reported recently in a mix group of pancreatic and advanced colon cancer patients with muscle and weight loss, including activation of immune, extracellular matrix, and coagulation pathways (10)." (page 16)

And acknowledge the limitations of not being a study designed to ascertain cachexia as follows: "Because the most common phenotype of PCa patients undergoing ADT is different from cancer cachexia, our study was neither designed nor powered to ascertain this condition. This will be the focus of future studies." (Page 19)

This Reviewer is also mentioning to us that the reasons to focus on down-regulated genes and not exploring much those up-regulated are not clear. While we understand it would not be fair to ask for further experiments/analyses at this stage, however, we would expect you to please include further Discussion in the manuscript about this point.

Response: We thank the reviewer for the request for further clarification. Bhatt at al recently reported on the association between weight and muscle mass loss and the muscle RNAome but do not provide insight into its association with "functional impairment", being muscle function or patient-reported outcomes such as anorexia or fatigue. We have added to the discussion the following to address this point. Moreover, we focused mainly on these down-regulated genes, as these were directly related to the study's *a priori* main goal: "to characterize the relationship between muscle mass, strength, endurance, PROs, and mitochondrial function". We have addressed this issue and now include an expanded interpretation of this in the revised manuscript as follows:

"We focused mainly on these down-regulated genes, as these were directly related to the study's *a priori* main goal to characterize the relationship between muscle mass, strength, endurance, PROs, and mitochondrial function. However, GSEA also highlighted upregulation of ECM remodelling potentially indicating increased muscle fibrosis and activation of inflammation-related pathways. This is consistent with prior findings in hypogonadal men where testosterone administration reduced serum inflammatory cytokines (11), and suggest that ADT may promote a pro-inflammatory state. Similar molecular signatures have also been reported recently in a mix group of pancreatic and advanced colon cancer patients with muscle and weight loss, including activation of immune, extracellular matrix, and coagulation pathways (10). This pro-inflammatory state and extracellular matrix remodelling phenotype has been further studied in skeletal muscle biopsies of female patients with pancreatic cancer cachexia, where complement activation, immune cell infiltration, and pathological collagen remodelling were observed compared to non-cancer controls (51). This suggests that ADT may potentiate cancer-related inflammation and fibrosis in skeletal muscle."

References:

1. Fearon K, Strasser F, Anker SD, Bosaeus I, Bruera E, Fainsinger RL, et al. Definition and classification of cancer cachexia: an international consensus. Lancet Oncol. 2011;12(5):489-95.
2. Liu ITT, Kesselheim AS, Cliff ERS. Clinical Benefit and Regulatory Outcomes of Cancer Drugs Receiving Accelerated Approval. Jama. 2024;331(17):1471-9.
3. Delfinis LJ, Bellissimo CA, Gandhi S, DiBenedetto SN, Garibotti MC, Thuhan AK, et al. Muscle weakness precedes atrophy during cancer cachexia and is linked to muscle-specific mitochondrial stress. JCI Insight. 2022;7(24).
4. Williams GR, Chen Y, Kenzik KM, McDonald A, Shachar SS, Klepin HD, et al. Assessment of Sarcopenia Measures, Survival, and Disability in Older Adults Before and After Diagnosis With Cancer. JAMA Netw Open. 2020;3(5):e204783.
5. Crawford J, Johnston M, Hancock M, Small S, Taylor R, Dalton J, et al. Enobosarm, a selective androgen receptor modulator (SARM) increases lean body mass (LBM) in advanced NSCLC patients: Updated results of two pivotal, international phase 3 trials. In: Cancer SCi, editor.2014. p. S30.
6. Temel JS, Abernethy AP, Currow DC, Friend J, Duus EM, Yan Y, et al. Anamorelin in patients with non-small-cell lung cancer and cachexia (ROMANA 1 and ROMANA 2): results from two randomised, double-blind, phase 3 trials. Lancet Oncol. 2016;17(4):519-31.
7. Fioretti F, von Haehling S, Coats AJS, Butler J, Del Fabbro E, Skipworth RJE, et al. Cachexia and Wasting in Chronic Illness: Regulatory and Clinical Trial Update. J Cachexia Sarcopenia Muscle. 2025;16(6):e70128.
8. Haseen F, Murray LJ, Cardwell CR, O'Sullivan JM, Cantwell MM. The effect of androgen deprivation therapy on body composition in men with prostate cancer: systematic review and meta-analysis. J Cancer Surviv. 2010;4(2):128-39.
9. Leong DP, Fradet V, Niazi T, Selvanayagam JB, Sabbagh R, Higano CS, et al. Adiposity and Muscle Strength in Men With Prostate Cancer and Cardiovascular Outcomes. JACC CardioOncol. 2024;6(5):761-71.
10. Bhatt BJ, Ghosh S, Mazurak V, Brun AQ, Bathe O, Baracos VE, et al. Molecular subtypes of human skeletal muscle in cancer cachexia. Nature. 2025;646(8086):973-82.
11. Malkin CJ, Pugh PJ, Jones RD, Kapoor D, Channer KS, Jones TH. The effect of testosterone replacement on endogenous inflammatory cytokines and lipid profiles in hypogonadal men. J Clin Endocrinol Metab. 2004;89(7):3313-8.

Response to Reviewer 3:

Suggested changes and rationale:

>Starting at line 249: "As in cancer cachexia, a condition defined by Fearon, et al. "an ongoing loss of skeletal muscle mass that leads to progressive functional impairment" (28), phase III clinical trials (14, 29) have reported increases in muscle mass without corresponding improvements in physical function or patient-reported outcomes, which has prevented regulatory approval. Regulatory agencies such as the U.S. Food and Drug Administration emphasize the need for improvements in clinically meaningful outcomes that reflect how patients feel or function rather than changes in muscle mass alone as the latter one is not considered a clinically meaningful and approvable endpoint (30).

>>This text is unclear and potentially misleading. Quoting Fearon et al (28) in this paragraph seems to be for the purpose of emphasizing that cachexia entails loss of muscle mass and function, however in that work there is no statement that loss of muscle function is a diagnostic criterion of cachexia, which taking this statement in context, the authors possibly believe. Not at all clear as to what "As in cancer cachexia" refers to in this sentence.

In this paragraph authors are framing the outcome of muscle function is the only pathway to regulatory approval, however this is not correct. The cited information about regulatory approval includes ref 14 (an appetite stimulating molecule) and ref 29 (a selective androgen receptor modulator) both studies completed prior to 2016, at a time when FDA was looking at physical functon(handgrip in ref 14, stair climb test in ref 29 [a study never published in full the citation is an abstract]) as a co-primary endpoint. Ref 30 is a literature review with no authorship or specific input from FDA or other regulatory agencies. The authors should note that in current trials of investigational cachexia therapeutics the FDA has dropped physical functioning as a co-primary endpoint in the Phase II/III e.g. NCT06989437 (Ponsegromab). In any case this discussion about regulatory approval is far outside of the scope of the present study.

>Starting on line 301 "This is also important in conditions such as cancer cachexia, where diagnostic criteria rely largely on weight loss (28) and therefore underestimate the burden experienced by PCa patients undergoing ADT, as illustrated by our findings and a body of literature showing disproportionate worsening in PROs and physical function despite modest decreases in muscle mass and no weight loss (45, 51).

>>This is a statement in the discussion concerning cachexia, which sits at the end of a paragraph concerning ADT, which seems unrelated to that paragraph and its purpose is unclear as the relationship between diagnostic criteria for cachexia and the content of refs 45, and 51 are obscure. This should also be removed.

>At line 332 "However, the functional consequences of these molecular changes in muscle were not assessed in that study, and thus, a direct link between these pathways and muscle function or PROs remains to be established in those settings.

>>Unclear reference to earlier part of the paragraph which "study" (or "studies as implied by the plural) is being referred to?

>Line 399 "Because the most common phenotype of PCa patients undergoing ADT is different from cancer cachexia, our study was neither designed nor powered to ascertain this condition."

>>This single sentence sits in the limitations section of the paper. I suggest that it be removed. It is unclear in the sense that the "phenotype" isn't stated and it does seem to be based in the fervent belief that none of the patients in this cohort could have cachexia, which is not true but also not necessary or essential to the discussion of the present work.

Response: We thank the reviewer for the insightful comments and in-depth response. The following lines and references were removed:

-lines 248-255: From "As in cancer cachexia...", to "...approvable endpoint (30)"

-lines 300-304: From "This is also important...", to "...no weight loss (45, 51)"

-lines 331-333: From "However, the functional consequences...", to "... to be established in those settings."

-line 398-400: From "Because the most common...", to "...to ascertain this condition."
